# Four New Highly Oxygenated Eremophilane Sesquiterpenes from an Endophytic Fungus *Boeremia exigua* Isolated from *Fritillaria hupehensis*

**DOI:** 10.3390/jof8050492

**Published:** 2022-05-08

**Authors:** Hong-Lian Ai, Xiao Lv, Ke Ye, Meng-Xi Wang, Rong Huang, Bao-Bao Shi, Zheng-Hui Li

**Affiliations:** School of Pharmaceutical Sciences, South-Central MinZu University, Wuhan 430074, China; aihonglian05@163.com (H.-L.A.); 2019110403@mail.scuec.edu.cn (X.L.); 2019110410@mail.scuec.edu.cn (K.Y.); 2021110499@mail.scuec.edu.cn (M.-X.W.); konglingniao1988@163.com (R.H.)

**Keywords:** *Boeremia exigua*, *Fritillaria hupehensis*, eremophilanes, boeremialanes, anti-inflammatory, NO production inhibition

## Abstract

Four new eremophilane-type sesquiterpenes, boeremialanes A–D (**1**–**4**) were obtained from solid substrate cultures of *Boeremia exigua* (Didymellaceae), an endophytic fungus isolated from *Fritillaria hupehensis* (Liliaceae). Boeremialanes A–C (**1**–**3**) are highly oxygenated eremophilanes with a benzoate unit attached at the C-13 position and are rarely found in nature. Their structures and absolute configurations were determined by extensive spectroscopic methods, electronic circular dichroism (ECD), and nuclear magnetic resonance (NMR) calculations with DP4+ analysis. Boeremialane D (**4**) potently inhibited nitric oxide production in lipopolysaccharide-treated RAW264.7 macrophages with an IC_50_ of 8.62 μM and was more potent than the positive control, pyrrolidinedithiocarbamate (IC_50_ = 23.1 μM).

## 1. Introduction

Eremophilane-type derivatives are structurally irregular and bicyclic natural products belonging to a small sesquiterpene family [1,2]. These eremophilane sesquiterpenes are biogenetically derived from farnesyl diphosphate in association with a methyl migration [3] and consist of three isoprene subunits [4]. The structural diversity of eremophilane analogs is due to oxidation occurring at different sites along the isopropyl side chain and bicyclic backbone to generate alcohol [5], acid [6], ester [7,8,9], furan [10,11], and lactone functionalities, with some of the alcohols further glycosylated [12]. Since the first eremophilane-type sesquiterpene was isolated from the wood oil of *Eremophila mitchellii* in 1932 [13], more than 650 biologically active eremophilane derivatives have been obtained [2,14]. In addition to the related analogs obtained from terrestrial plants [15,16] and marine fungi [17,18], plant endophytic fungi are recognized as a new source of derivatives eremophilane [19,20]. Due to their special structural features and various functional groups, eremophilane-type sesquiterpenes possess a lot of biological activities such as anti-inflammatory [21], antitumor [10], and antibacterial [22,23] activities, which have received increasing interest in the recent years. As part of our ongoing efforts to discover bioactive terpenoids derived from endophytic fungi [24,25,26,27], a chemical investigation on the cultural broth of *B. exigua* in rice medium was carried out. As a result, four new highly oxygenated eremophilane-type sesquiterpenes, boeremialanes A–D (**1**–**4**), were isolated from cultures of the fungus *B. exigua*. The new structures were established by extensive spectroscopic methods, ECD and NMR calculations, as well as DP4+ analysis. All compounds were tested for their anti-inflammatory activities on nitric oxide production in LPS-induced RAW264.7 macrophages. Herein, details of the isolation, structural elucidation and bioactivities of the compounds are reported.

## 2. Materials and Methods

### 2.1. General Experimental Procedures

Optical rotations were measured with an Autopol IV polarimeter (Rudolph, Hackettstown, NJ, USA). UV spectra were measured on a UV-2450 spectrometer (Hitachi High-Technologies, Tokyo, Japan). CD spectra were recorded with an Applied Photophysics spectrometer (Chirascan, New Haven, CT, USA). One-dimensional and 2D spectra were recorded on a Bruker AV-600 spectrometer (Bruker, Karlsruhe, Germany) with TMS as an internal standard. HRESIMS spectra were recorded on Q Exactive Obitrap mass spectrometer (ThermoFisher Scientific, Waltham, MA, USA). Medium pressure liquid chromatography (MPLC) was performed on a Biotage SP1 System and column packed with RP-18 gel (Biotage, Uppsala, Sweden). Silica gel (Qingdao Marine Chemical Factory, Qingdao, China), RP-18 gel (Fuji Silysia Chemical Factory, Kasugai, Japan), and Sephadex LH-20 (Pharmacia Fine Chemical Factory, Uppsala, Sweden) were used for column chromatography (CC). Semi-preparative HPLC experiments were carried on Agilent 1260 HPLC with Zorbax SB-C_18_ column (Agilent, Palo Alto, CA, USA, 5 μm, 9.4 mm × 150 mm). Fractions were monitored by TLC (GF 254, Qingdao Haiyang Chemical Factory, Qingdao, China), and spots were visualized by heating silica gel plates sprayed with vanillin and 10% H_2_SO_4_ in EtOH.

### 2.2. Culture and Fermentation of Fungal Material

The strain *B. exigua* was isolated from the healthy leaf tissue of *Fritillaria hupehensis* Hsiao. It was identified by Dr. Hong-Lian Ai (South-Central MinZu University). The ITS sequence of this strain is almost identical to the strain deposited in Genbank with accession number MT154621.1 (max identity: 100%, query cover: 100%). The fungal specimen is deposited at South-Central MinZu University, China. The strain was cultured on PDA medium for 8 days, and then was cut into small pieces to incubate solid rice medium to culture for further 30 days at 25 °C (50 g rice, 50 mL water, in each 500 mL Erlenmeyer flask, the total weight of rice was 17 kg).

### 2.3. Extraction and Isolation

The rice fermentation product of *B. exigua* (17 kg) was extracted five times with methanol to yield a crude extract after evaporation under vacuum. The crude extract was partitioned between water and EtOAc to give an EtOAc layer. The extract (800 g) of the organic layer was subjected to column chromatography over silica gel (200–300 mesh, CH_2_Cl_2_-MeOH, step gradient elution 1:0, 20:1, 10:1, 5:1, 2:1, 1:1, and 0:1) to obtain six fractions (A–F). Fr. C (35.8 g) was fractionated by MPLC over an RP-18 silica gel column and eluted with MeOH-H_2_O (from 20:80 to 90:10, *v*/*v*) to yield five subfractions (C_1_–C_5_). Fraction C_1_ (5.8 g) was separated on a silica gel column (200–300 mesh, CH_2_Cl_2_-MeOH, step gradient elution 10:1, 4:1, 2:1, and 1:1) to give four subfractions (Fr. C_1-1_–Fr. C_1-4_). Then, Fr. C_1-1_ was purified by semi-preparative HPLC (CH_3_CN/H_2_O from 25:75 to 35:65 over 30 min) to obtain compound **4** (2.0 mg, retention time (*t*_R_) = 12.0 min). Fr. C_1-2_ was purified by semi-preparative HPLC (CH_3_CN/H_2_O from 28:72 to 36:64 over 28 min) to obtain compound **1** (5.1 mg, *t*_R_ = 15.6 min), compound **2** (7.2 mg, *t*_R_ = 17.8 min), and compound **3** (8.1 mg, *t*_R_ = 19.7 min).

*Boeremialane A* (**1**): Yellowish oil; ${\left[\alpha \right]}_{D}^{27}$ 102.5 (*c* 0.1, MeOH); UV (MeOH) *λ*_max_ (log *ε*) 205 (3.61), 230 (3.46) nm; ^1^H NMR (600 MHz) and ^13^C NMR (150 MHz, methanol-*d*_4_), see Table 1; HRESIMS (positive) *m/z* 483.16220 [M + Na]^+^ (calcd for C_24_H_28_O_9_Na^+^, 483.16255).

*Boeremialane B* (**2**): Yellowish oil; ${\left[\alpha \right]}_{D}^{27}$ 50.0 (*c* 0.1, MeOH); UV (MeOH) *λ*_max_ (log *ε*) 210 (3.44), 235 (3.16) nm; ^1^H NMR (600 MHz) and ^13^C NMR (150 MHz, methanol-*d*_4_), see Table 1; HRESIMS (positive) *m/z* 441.15195 [M + Na]^+^ (calcd for C_22_H_26_O_8_Na^+^, 441.15199).

*Boeremialane C* (**3**): Yellowish oil; ${\left[\alpha \right]}_{D}^{27}$ 216.0 (*c* 0.1, MeOH); UV (MeOH) *λ*_max_ (log *ε*) 205 (3.75), 250 (3.80) nm; ^1^H NMR (600 MHz) and ^13^C NMR (150 MHz, methanol-*d*_4_), see Table 1; HRESIMS (positive) *m/z* 441.15182 [M + Na]^+^ (calcd for C_22_H_26_O_8_Na^+^, 441.15199).

*Boeremialane D* (**4**): Yellow amorphous powder; ${\left[\alpha \right]}_{D}^{27}$ 136.0 (*c* 0.1, MeOH); UV (MeOH) *λ*_max_ (log *ε*) 240 (3.47) nm; ^1^H NMR (600 MHz) and ^13^C NMR (150 MHz, methanol-*d*_4_), see Table 1; HRESIMS (positive) *m/z* 345.13064 [M + Na]^+^ (calcd for C_17_H_22_O_6_Na^+^, 345.13086).

### 2.4. Quantum Chemical Calculations

The initial conformational analysis of compounds **1**–**4** was performed using the Monte Carlo search algorithm via the MMFF94 molecular mechanics force field [28], with the aid of the Spartan 16 program package that resulted in some relatively favorable conformations with an energy range of 3 kcal/mol above the global minimum. The minimum energy conformers of the resulting force field were optimized in vacuum with the M06-2X/def2-SVP level, and implemented in the Gaussian 09 software package by the Density functional theory [29]. At the same time, harmonic vibrational frequencies were also measured to confirm the lack of imaginary frequencies of the finally optimized conformers. These primary conformations were subjected to theoretical calculations of ECD utilizing time-dependent density functional theory (TDDFT) calculations at the M06-2X/def2-SVP level in MeOH using the polarizable continuum model (PCM) solvent model. The energies, oscillator strengths, and rotational strengths of each conformation were determined with the Gaussian 09 software package. Theoretical calculations of ECD spectra for each part were then approximated by the Gaussian distribution. The final ECD spectrum of the individual conformers was summed up on the basis of the Boltzmann-weighed population contribution by the SpecDisv1.71 [30]. DFT GIAO ^13^C NMR calculations were performed on the mPW1PW91/6-31 + G(d,p)//M06-2X/def2-SVP level of theory [31]. The solvent effect was accounted for by using methanol in the calculations to mimic the experimental conditions. The ^13^C NMR chemical shifts in compound **1** were considered the average values of the same atoms in the different conformers. We took the relative Gibbs free energy as the weighting factor and used the Boltzmann distribution to find the average values. The overall theoretical NMR data were analyzed using DP4+ probability [32].

### 2.5. Nitric Oxide Production Inhibitory Assay 

The anti-inflammatory effect of Raw264.7 macrophages was studied and cultured in Dulbecco’s modified eagle medium (DMEM, HyClone, Logan, UT, USA) with 10% fetal bovine serum (FBS, PAN, Aidenbach, Germany) in a humidified incubator (5% CO_2_, 37 °C). RAW264.7 cells (5 × 10^4^ cells/well) were seeded into a 96-well multiplate for 12 h. After 12 h of incubation, the cells were treated with LPS (1 μg/mL) and different concentrations of the tested compounds (**1**–**4**, 20 μM) for 18 h. A Griess reagent kit (Promega, Madison, WI, USA) was used to measure the amount of nitrite, a stable metabolite of Nitric Oxide (NO), in the supernatants. Briefly, 50 μL of each culture medium was added to a 96-well plate, and then the same volume of sulfanilamide solution was added. After incubation at room temperature for 5 min, 50 μL of N-1-naphthylethylenediamine dihydrochloride solution was added to all wells. The absorption at 540 nm was measured by a microplate reader after 10 min incubation at room temperature [33]. The IC_50_ values were calculated by GraphPad Prism 6 software. Cell viability was determined with the MTT (3-[4,5-dimethylthiazol-2-yl]-2,5 diphenyl tetrazolium bromide) assay. Pyrrolidine dithiocarbamate (PDTC, Sigma−Aldrich, St Louis, MO, USA) was used as a positive control.

## 3. Results and Discussion

**Figure 1:**
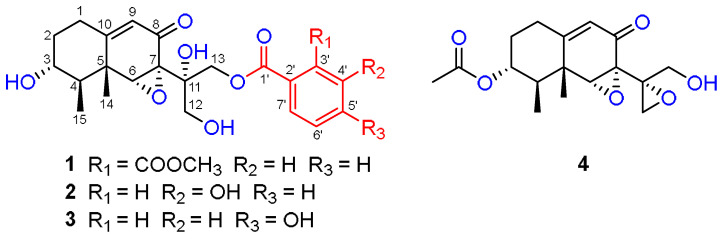
Chemical structures of compounds **1**–**4**.

**Figure 2:**
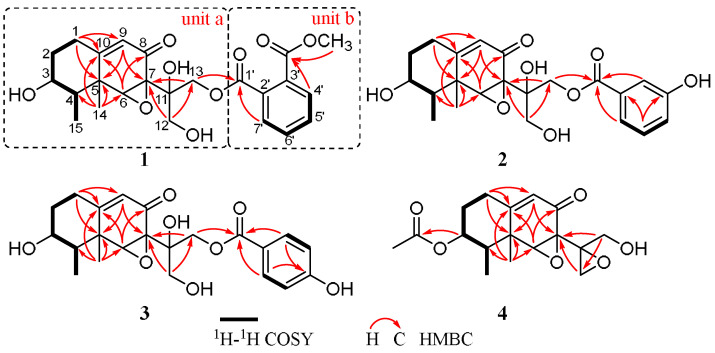
Key HMBC and ^1^H-^1^H COSY correlations of compounds **1**–**4**.

Boeremialane A (**1**) was obtained as a yellowish oil, and the molecular formula of compound **1** was determined to be C_24_H_28_O_9_ from the HRESI mass spectrum ([M + Na]^+^ data, found 483.16220, calcd. 483.16255). The ^1^H and ^13^C NMR data of compound **1** indicated the presence of two methyl groups (*δ*_C_ 11.6 and 18.3), four methylene groups (*δ*_C_ 31.6, 36.2, 65.6, and 69.3), eight methine groups (*δ*_C_ 71.1, 45.9, 63.9, 121.8, 130.3, 129.7, 132.9, and 132.3), one carbonyl (*δ*_C_ 195.5), two ester carbonyls (*δ*_C_ 169.6 and 168.9), three sp3 quaternary carbons (*δ*_C_ 42.2, 62.9, and 73.9), and three sp2 quaternary carbons (*δ*_C_ 166.5, 134.3, and 132.8) (Table 1, Figures S5–S10). In the HMBC spectrum (Figure 1 and Figure 2), a singlet for the Me-14 at *δ*_H_ 0.64 (3H, s, H-14) showed correlations to C-4 (*δ*_C_ 45.9), C-6 (*δ*_C_ 63.9), C-10 (*δ*_C_ 166.5), and a sp^3^ quaternary carbon at *δ*_C_ 42.2 (C-5). This was very important for the establishment of the three C-C bonds of C-4, C-10, and C-6 with C-5. In addition, the HMBC spectrum showed correlations from H-1 (*δ*_H_ 2.41 and 2.24) to C-5 and C-10, and the ^1^H-^1^H COSY spectrum analysis (H-1/H-2/H-3/H-4/H-15) together with a characteristic oxygenated methine carbon (*δ*_C_ 71.1, C-3) determined a 1,2,3,3,4-pentasubstituted cyclohexane ring of compound **1**. A 2-cyclohexen-1-one ring was inferred by the HMBC correlations from H-6 (*δ*_H_ 3.80) to C-5, C-7, C-8, and C-10 and from H-9 (*δ*_H_ 5.61) to C-5 and C-7, with the connection to the cyclohexane ring by the C-5/C-10 position on the basis of the HMBC correlations of H-1/C-9 and H-4/C-6 (Figure 2). The HMBC correlations from H-12 (*δ*_H_ 4.19 and 3.76) to C-11 and C-13 and from H-13 (*δ*_H_ 4.64 and 4.59) to C-11 and C-12 together with the downfield shifts of C-11 (*δ*_C_ 73.9), C-12 (*δ*_C_ 65.6), and C-13 (*δ*_C_ 69.3) indicated the existence of a highly oxidized propane group, which was linked to the position of C-7, as evidenced by the HMBC correlations from H-12 to C-7 and from H-13 to C-7. These data, as well as other HMBC correlations, suggested that unit A was a tetrol phaseolinone [34], which had been previously isolated from *Macrophomina phaseolina.*

For unit B, the ^1^H NMR spectrum of compound **1** revealed the signals for four aromatic protons (*δ*_H_ 7.77, 7.62, 7.61, and 7.59). In the ^1^H-^1^H COSY spectrum, a disubstituted benzene ring was identified by four continuous aromatic protons at *δ*_H_ 7.77 (1H, d, H-4’), 7.62 (1H, t, H-5’), 7.61 (1H, t, H-6’), and 7.59 (1H, d, H-7’), and two aromatic doublets and two aromatic triplets with the same coupling constant (*J* = 6.4 Hz) indicated an ortho-disubstituted benzene group. A carbomethoxy substituent in the benzene ring was identified by the HMBC correlations from H-4’ to the carbomethoxy substituent (*δ*_C_ 168.9). Similarly, an ester carbonyl carbon (*δ*_C_ 169.6) was positioned at C-2’ based on observed cross-peaks at H-7’/C-1’. The HMBC correlations from H-13 to the ester carbonyl carbon (C-1’) confirmed the 13,1’-ester linkage of the two substructures. Thus, the planar structure of compound **1** was elucidated as shown in Figure 1. 

**Figure 3:**
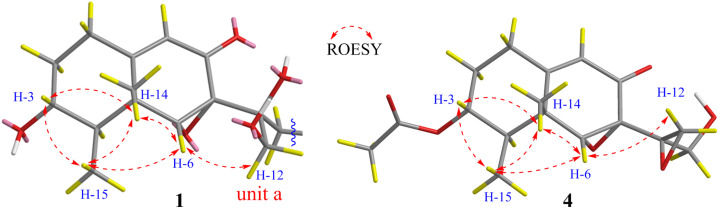
Key ROESY correlations of compounds **1** and **4**.

**Figure 4:**
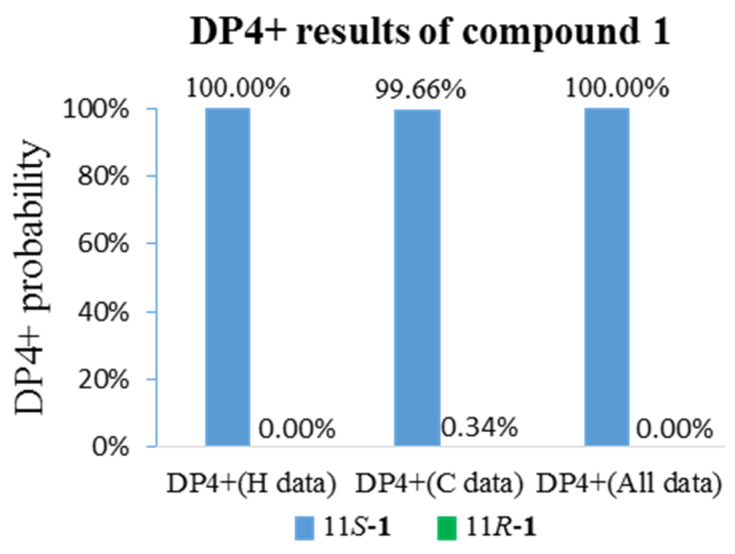
qccNMR coupled with DP4+ probability analysis of compound **1**.

**Figure 5:**
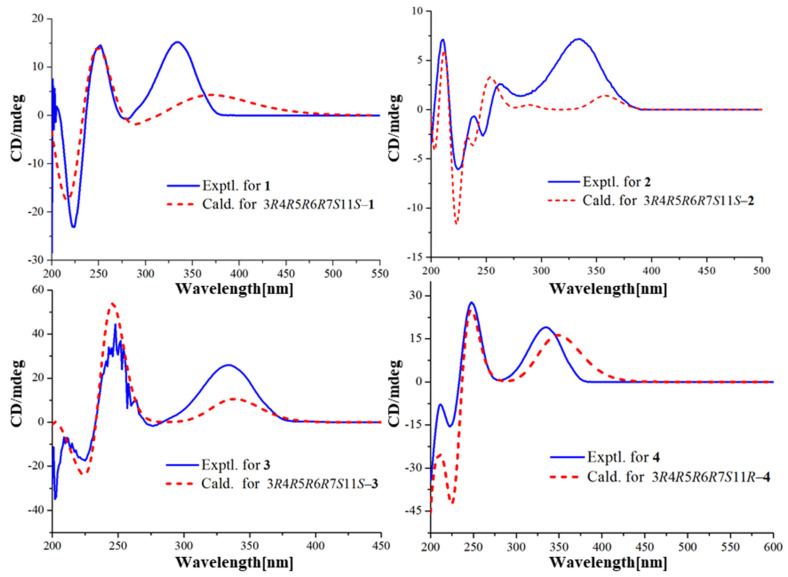
Experimental and calculated ECD spectra of compounds **1**–**4** at the M06-2X/def2-SVP level in methanol.

The configuration of boeremialane A (**1**) was established by ROESY experiments and quantum chemistry calculations. The ROESY correlations of H-3/H_3_-15, H-3/H_3_-14, H-6/H_3_-14, and H-6/H_3_-15 suggested that they were *β*-oriented (Figure 3). In addition, to determine the configuration of C-11 in the flexible bond, nuclear magnetic resonance (NMR) calculations of two epimers, 11*S*-**1** and 11*R*-**1**, were carried out. The two epimers were subjected to a strict conformational screening procedure; then, the NMR chemical shifts were calculated at the mPW1PW91/6-31 + G(d,p)//M06-2X/def2-SVP level of theory with the PCM solvent in methanol. The DP4+ analysis identified 11*S*-**1** as the most likely structure of compound **1** with 100.00% DP4+ probability (all data) (Figure 4 and Table S1). Finally, the absolute configuration of compound **1** was resolved by comparing the calculated and experimental ECD data using time-dependent density-functional theory (TDDFT). The theoretical spectrum of compound **1** showed an excellent fit with the experimental plot recorded in MeOH (Figure 5 and Figure S1), which supported an absolute configuration of 3*R*, 4*R*, 5*R*, 6*R*, 7*S*, and 11*S.* Thus, the structure of compound **1** was determined, and it was named boeremialane A.

Boeremialane B (**2**) was obtained as a yellowish oil, and the molecular formula was determined to be C_22_H_26_O_8_ from the HRESI mass spectrum data ([M + Na]^+^, found 441.15195, calcd. 441.15199). The ^1^H and ^13^C NMR data of compound **2** indicated the presence of two methyl groups (*δ*_C_ 11.6 and 19.0), four methylene groups (*δ*_C_ 31.6, 36.3, 65.6, and 67.5), eight methine groups (*δ*_C_ 71.1, 46.0, 64.1, 121.8, 117.3, 121.3, 130.5, and 121.8), one carbonyl (*δ*_C_ 195.4), one ester carbonyl (*δ*_C_ 168.1), three sp3 quaternary carbons (*δ*_C_ 42.3, 62.8, and 74.2), and three sp2 quaternary carbons (*δ*_C_ 166.5, 132.4, and 158.8) (Table 1 and Figures S12–S17). The ^1^H and ^13^C NMR data of compound **2** were structurally similar to those of compound **1**, except for the absence of a carbomethoxy group at *δ*_C_ 168.9 and 53.5 in compound **1** and the presence of an additional hydroxy group in compound **2**. The hydroxyl group at C-4’ was evident from the downfield shift of C-4’ (*δ*_C_ 158.8) as well as the HMBC correlations from H-13 to the sp2 quaternary carbon (C-4’) (Figure 2). The relative configuration of compound **2** was the same as that found in compound **1** based on the ROESY correlations of H-3/H_3_-15, H-3/H_3_-14, H-6/H_3_-14, and H-6/H_3_-15 (Figure 3). Finally, the absolute configuration of **2** was determined by ECD calculations on the M06-2X/def2-SVP (IEFPCM, MeOH) level of theory. The experimental ECD spectrum of compound **2** fits well with the calculated spectrum of 3*R*, 4*R*, 5*R*, 6*R*, 7*S*, and 11*S*-**2** (Figure 5 and Figure S2). Therefore, the structure of compound **2** was determined, and it was given the name boeremialane B.

Boeremialane C (**3**) has a molecular formula of C_35_H_40_O_8_ according to its HRESIMS ion at *m/z* 441.15182 [M + Na]^+^ (calcd for C_22_H_26_O_8_Na, 441.15199). The ^1^H and ^13^C NMR data of **3** (Table 2 and Figures S19–S24) were structurally similar to those of compound **2**, except for the presence of a para-substituted benzene ring of the benzoate unit. This difference was supported by the HMBC correlations from H-3’ (7’) (*δ*_H_ 7.81) to C-1’ (*δ*_C_ 168.4) and C-5’ (*δ*_C_ 165.9) along with the COSY correlations between H-3’ (7’)/H-4’ (6’) (*δ*_H_ 6.74) (Figure 2). The ECD spectrum of compound **3** was similar to that of compound **1** with negative exciton coupling at 211 nm and positive exciton coupling at 241 nm (Figure S33), which indicated that they share the identical absolute configuration. Therefore, the absolute configuration of **3** was defined as 3*R*, 4*R*, 5*R*, 6*R*, 7*S*, and 11*S*. This presumption was confirmed by comparative analysis of calculated and experimental ECD spectra. The experimental ECD spectrum of **3** fits well with the calculated spectrum of 3*R*, 4*R*, 5*R*, 6*R*, 7*S*, and 11*S*-**3** (Figure 5 and Figure S3). Thus, the structure of **3** was determined and named boeremialane C. 

Boeremialane D (**4**) was obtained as a yellow amorphous powder, and the molecular formula, C_17_H_22_O_6_, was determined by (+)-HRESIMS, which showed an [M + Na]^+^ ion at *m*/*z* 345.13064 (calcd for C_17_H_22_O_6_Na: 345.13086). The ^1^H and ^13^C NMR data of compound **4** indicated the presence of three methyl groups (*δ*_C_ 18.6, 11.4, and 21.0), four methylene groups (*δ*_C_ 31.2, 32.5, 48.3, and 62.0), four methine groups (*δ*_C_ 74.2, 43.1, 65.5, and 121.7), one carbonyl (*δ*_C_ 194.2), one ester carbonyl (*δ*_C_ 172.4), three sp3 quaternary carbons (*δ*_C_ 42.4, 62.1, and 59.0), and one sp2 quaternary carbon (*δ*_C_ 165.4) (Table 2 and Figures S26–S31). The ^1^H and ^13^C NMR data of compound **4** were structurally similar to those of phaseolinone [35], except for the appearance of an additional acetyl group in compound **4**. The attachment of this acetyl group at C-3 was supported by the HMBC correlation from the H-3 to the ester carbonyl carbons (*δ*_C_ 172.4). The relative configuration of compound **4** was the same as that found in compound **1** based on the ROESY correlations of H-3/H_3_-15, H-3/H_3_-14, H-6/H_3_-14, and H-6/H_3_-15 (Figure 3). Similar to compound **3**, the tendencies of the ECD curves of compounds **4** and **1** with negative exciton coupling at 225 nm and positive exciton coupling at 250 and 337 nm were relatively consistent (Figure S33, Supporting Information), which indicated that they have an the identical absolute configuration. In addition, the identity of the measured ECD and calculated ECD spectrum of compound **4** further confirmed this conclusion (Figure 5 and Figure S4). Therefore, the structure of compound **4** was determined, and it was given the name boeremialane D.

**Figure 6:**
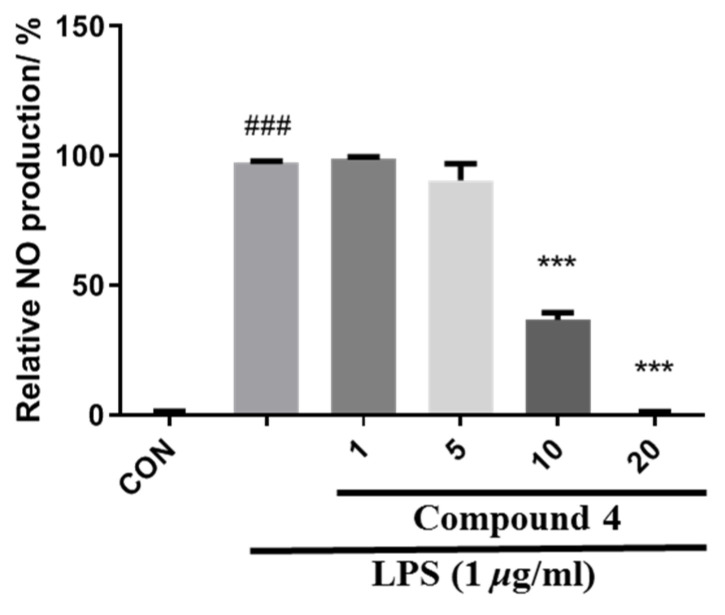
Effects of compound **4** isolated from *B. exigua* on NO production in LPS-stimulated RAW 264.7 macrophages. Cells were pretreated with the indicated concentrations of the isolates for 1 h and then stimulated with LPS (1 μg/mL) for 24 h. The NO levels in the culture medium were measured by the MTT assay. ### *p* < 0.0001 vs. control. *** *p* < 0.0001 vs. LPS-stimulated group.

All compounds were evaluated for their inhibition of NO production in LPS-treated RAW264.7 macrophages. As a result, compound **4** showed certain inhibitory activity with IC_50_ values of 8.62 μM, which was more potent than the positive control, pyrrolidinedithiocarbamate (IC_50_ = 23.1 μM) (Figure 6).

## 4. Conclusions

In summary, the structures of four new eremophilane-type sesquiterpenes (**1**–**4**) were unambiguously determined by analyses of their HRESI and NMR spectroscopic data, with the absolute configuration being determined by quantum chemistry calculations. Boeremialanes A–C (**1**–**3**) are highly oxygenated eremophilanes with the benzoate unit attached at the C-13 position, and only one such natural compound has been discovered to date [35]. Compound **4** exhibited potent inhibition against NO production in LPS-activated RAW 264.7 macrophages, suggesting that it is a new chemical entity for anti-inflammatory effects. The present research provides new insights into understanding the structural diversity and interesting biological activities of eremophilane sesquiterpenes.

## Figures and Tables

**Table 1:** ^1^H and ^13^C NMR Spectroscopic Data for **1** and **2** in Methanol-*d*_4_ (*δ* in ppm, *J* in Hz).

No.	*δ*_H_ (1) *^a^*	*δ*_C_ (1) *^b^*	*δ*_H_ (2) *^a^*	*δ*_C_ (2) *^b^*
1	2.41 (tdd, 14.4, 5.0, 1.9)	31.6, CH_2_	2.51 (tdd, 14.4, 5.0, 1.9)	31.6, CH_2_
	2.24 (dt, 14.4, 4.1)		2.28 (dt, 14.4, 3.5)	
2	2.03 (dd, 12.3, 4.4)	36.2, CH_2_	2.07 (dd, 12.2, 4.4)	36.3, CH_2_
	1.27 (ddd, 12.3, 5.0, 4.1)		1.29 (ddd, 12.2, 5.0, 3.5)	
3	3.44 (td, 10.5, 4.4)	71.1, CH	3.53 (td, 10.5, 4.4)	71.1, CH
4	1.63 (dq, 10.5, 6.7)	45.9, CH	1.70 (dq, 10.5, 6.7)	46.0, CH
5		42.2, C		42.3, C
6	3.80 (s)	63.9, CH	3.91 (s)	64.1, CH
7		62.9, C		62.8, C
8		195.5, C		195.4, C
9	5.61 (d, 1.9)	121.8, CH	5.66 (d, 1.9)	121.8, CH
10		166.5, C		166.5, C
11		73.9, C		74.2, C
12	4.19 (d, 11.6)	65.6, CH_2_	4.17 (d, 11.6)	65.5, CH_2_
	3.76 (d, 11.6)		3.81 (d, 11.6)	
13	4.64 (d, 11.5)	69.3, CH_2_	4.83 (d, 11.7)	67.5, CH_2_
	4.59 (d, 11.5)		4.44 (d, 11.7)	
14	0.64 (s)	18.3, CH_3_	1.03 (s)	19.0, CH_3_
15	1.16 (d, 6.7)	11.6, CH_3_	1.23 (d, 6.7)	11.6, CH_3_
1’		169.6, C		168.1, C
2’		134.3, C		132.4, C
3’		132.8, C	7.37 (dd, 2.6, 1.3)	117.3, CH
4’	7.77 (dd, 6.4, 2.1)	130.3, CH		158.8, C
5’	7.62 (td, 6.4, 2.7)	129.7, CH	7.00 (dd, 7.9, 2.6)	121.3, CH
6’	7.61 (td, 6.4, 2.1)	132.9, CH	7.25 (t, 7.9)	130.5, CH
7’	7.59 (dd, 6.4, 2.7)	132.3, CH	7.44 (dd, 7.9, 1.3)	121.8, CH
COOCH_3_	3.85 (s)	53.5, CH_3_		
COOCH_3_		168.9, C		

*^a^* Recorded at 600 MHz, *^b^* Recorded at 150 MHz.

**Table 2:** ^1^H and ^13^C NMR Spectroscopic Data for **3** and **4** in Methanol-*d*_4_ (*δ* in ppm, *J* in Hz).

No.	*δ*_H_ (3) *^a^*	*δ*_C_ (3) *^b^*	*δ*_H_ (4) *^a^*	*δ*_C_ (4) *^b^*
1	2.50 (tdd, 14.4, 4.8, 1.8)	31.6, CH_2_	2.41 (tdd, 14.6, 5.0, 1.8)	31.2, CH_2_
	2.28 (dt, 14.4, 3.5)		2.39 (dt, 14.6, 4.0)	
2	2.07 (dd, 12.5, 4.4)	36.3, CH_2_	2.15 (dd, 12.3, 4.4)	32.5, CH_2_
	1.29 ddd, 12.5, 4.8,3.5		1.40 (ddd,12.3, 5.0, 4.0)	
3	3.53 (td, 10.6, 4.4)	71.1, CH	4.91 (td, 10.5, 4.4)	74.2, CH
4	1.69 (dq, 10.6, 6.8)	45.9, CH	1.95 (dq, 10.5, 6.8)	43.1, CH
5		42.2, C		42.4, C
6	3.89 (s)	64.1, CH	3.63 (s)	65.5, CH
7		62.8, C		62.1, C
8		195.3, C		194.2, C
9	5.65 (d, 1.8)	121.8, CH	5.75 (d, 1.8)	121.7, CH
10		166.4, C		165.4, C
11		74.3, C		59.0, C
12	4.17 (d, 11.6)	65.5, CH_2_	2.87 (d, 5.1)	48.3, CH_2_
	3.81 (d, 11.6)		2.66 (d, 5.1)	
13	4.82 (d, 11.7)	67.2, CH_2_	4.07 (d, 12.3)	62.0, CH_2_
	4.39 (d, 11.7)		3.72 (d, 12.3)	
14	1.02 (s)	19.1, CH_3_	1.26 (s)	18.6, CH_3_
15	1.22 (d, 6.8)	11.6, CH_3_	1.14 (d, 6.8)	11.4, CH_3_
1’		168.4, C		
2’		120.5, C		
3’	7.81 (d, 8.8)	133.1, CH		
4’	6.74 (d, 8.8)	116.8, CH		
5’		165.9, C		
6’	6.74 (d, 8.8)	116.8, CH		
7’	7.81 (d, 8.8)	133.1, CH		
CH_3_CO			2.06 (s)	21.0, CH_3_
CH_3_CO				172.4, C

*^a^* Recorded at 600 MHz, *^b^* Recorded at 150 MHz.

## Data Availability

Not applicable.

## Supplementary Materials

The following are available online at https://www.mdpi.com/article/10.3390/jof8050492/s1. Table S1. DP4+ analysis results of **1a** (Isomer 1) and **1b** (Isomer 2). Table S2. Experimental and calculated ^13^C NMR chemical shifts of **1a** and **1b**. Table S3. Important thermodynamic parameters of the M06-2X/def2-SVP optimized conformers of **1a** in the gas phase. Table S4. Conformational analysis of the M06-2X/def2-SVP optimized conformers of **1a** in the gas phase (T = 298.15 K). Table S5. Important thermodynamic parameters of the M06-2X/def2-SVP optimized conformers of **2** in the gas phase. Table S6. Conformational analysis of the M06-2X/def2-SVP optimized conformers of **2** in the gas phase (T = 298.15 K). Table S7. Important thermodynamic parameters of the M06-2X/def2-SVP optimized conformers of **3** in the gas phase. Table S8. Conformational analysis of the M06-2X/def2-SVP optimized conformers of **3** in the gas phase (T = 298.15 K). Table S9. Important thermodynamic parameters of the M06-2X/def2-SVP optimized conformers of **4** in the gas phase. Table S10. Conformational analysis of the M06-2X/def2-SVP optimized conformers of **4** in the gas phase (T = 298.15 K). Table S11. Cartesian coordinates for the low-energy optimized conformers of **1**–**4** at M06-2X/def2-SVP level. Figure S1. Experimental ECD spectra and calculated ECD spectra of **1**. Figure S2. Experimental ECD spectra and calculated ECD spectra of **2**. Figure S3. Experimental ECD spectra and calculated ECD spectra of **3**. Figure S4. Experimental ECD spectra and calculated ECD spectra of **4**. Figure S5. ^1^H NMR spectrum of **1** in CD_3_OD. Figure S6. ^13^C NMR spectrum of **1** in CD_3_OD. Figure S7. HSQC spectrum of **1** in CD_3_OD. Figure S8. HMBC spectrum of **1** in CD_3_OD. Figure S9. COSY spectrum of **1** in CD_3_OD. Figure S10. ROESY spectrum of **1** in CD_3_OD. Figure S11. HRMS spectrum of **1.** Figure S12. ^1^H NMR spectrum of **2** in CD_3_OD. Figure S13. ^13^C NMR spectrum of **2** in CD_3_OD. Figure S14. HSQC spectrum of **2** in CD_3_OD. Figure S15. HMBC spectrum of **2** in CD_3_OD. Figure S16. COSY spectrum of **2** in CD_3_OD. Figure S17. Roesy spectrum of **2** in CD_3_OD. Figure S18. HRMS spectrum of **2**. Figure S19. ^1^H NMR spectrum of **3** in CD_3_OD. Figure S20. ^13^C NMR spectrum of **3** in CD_3_OD. Figure S21. HSQC spectrum of **3** in CD_3_OD. Figure S22. HMBC spectrum of **3** in CD_3_OD. Figure S23. COSY spectrum of **3** in CD_3_OD. Figure S24. Roesy spectrum of **3** in CD_3_OD. Figure S25. HRMS spectrum of **3**. Figure S26. ^1^H NMR spectrum of **4** in CD_3_OD. Figure S27. ^13^C NMR spectrum of **4** in CD_3_OD. Figure S28. HSQC spectrum of **4** in CD_3_OD. Figure S29. HMBC spectrum of **4** in CD_3_OD. Figure S30. COSY spectrum of **4** in CD_3_OD. Figure S31. Roesy spectrum of **4** in CD_3_OD. Figure S32. HRMS spectrum of **4**. Figure S33. CD spectrum of **1**–**4** in MeOH.
