# Supplementary material for: Four New Highly Oxygenated Eremophilane Sesquiterpenes from an Endophytic Fungus Boeremia exigua Isolated from Fritillaria hupehensis
Source: J Fungi (Basel). 2022 May 8;8(5):492. doi: 10.3390/jof8050492 (PMC9146970; doi:10.3390/jof8050492)

*Supplementary materials for*

# Four New Highly Oxygenated Eremophilane Sesquiterpenes from an Endophytic Fungus *Boeremia exigua* Isolated from *Fritillaria hupehensis*

Hong-Lian Ai,[⊥] Xiao Lv,[⊥] Ke Ye, Meng-Xi, Wang, Rong Huang, Bao-Bao Shi,* and Zheng-Hui Li*

School of Pharmaceutical Sciences, South-Central MinZu University, Wuhan 430074, People's Republic of China

*Corresponding Author.

E-mail: shibb0505@163.com (B. B. Shi); lizhenghui@mail.scuec.edu.cn (Z. H. Li)

# Table of Contents


**Quantum chemical calculation** .......... 4

**1 NMR computational details for compound 1** .......... 4

Table S1. DP4+ analysis results of **1a** (Isomer 1) and **1b** (Isomer 2). .......... 5

Table S2. Experimental and calculated $^{13}$C NMR chemical shifts of **1a** and **1b**. .......... 6

**2 ECD computational details of compound 1** .......... 6

Figure S1. Experimental ECD spectra and calculated ECD spectra of **1**. .......... 6

Table S3. Important thermodynamic parameters of the M06-2X/def2-SVP optimized conformers of **1a** in the gas phase .......... 6

Table S4. Conformational analysis of the M06-2X/def2-SVP optimized conformers of **1a** in the gas phase (T=298.15 K) .......... 7

**3 ECD computational details of compound 2** .......... 7

Figure S2. Experimental ECD spectra and calculated ECD spectra of **2**. .......... 7

Table S5. Important thermodynamic parameters of the M06-2X/def2-SVP optimized conformers of **2** in the gas phase .......... 7

Table S6. Conformational analysis of the M06-2X/def2-SVP optimized conformers of **2** in the gas phase (T=298.15 K) .......... 8

**4 ECD computational details of compound 3** .......... 8

Figure S3. Experimental ECD spectra and calculated ECD spectra of **3**. .......... 8

Table S7. Important thermodynamic parameters of the M06-2X/def2-SVP optimized conformers of **3** in the gas phase .......... 8

Table S8. Conformational analysis of the M06-2X/def2-SVP optimized conformers of **3** in the gas phase (T=298.15 K) .......... 9

**5 ECD computational details of compound 4** .......... 9

Figure S4. Experimental ECD spectra and calculated ECD spectra of **4**. .......... 9

Table S9. Important thermodynamic parameters of the M06-2X/def2-SVP optimized conformers of **4** in the gas phase .......... 9

Table S10. Conformational analysis of the M06-2X/def2-SVP optimized conformers of **4** in the gas phase (T=298.15 K) .......... 10

Table S11. Cartesian coordinates for the low-energy optimized conformers of **1-4** at M06-2X/def2-SVP level. .......... 10

**Spectroscopic data** .......... 27

Figure S5. $^{1}$H NMR spectrum of **1** in $CD_3OD$ .......... 27

Figure S6 $^{13}$C NMR spectrum of **1** in $CD_3OD$ .......... 28

Figure S7 HSQC spectrum of **1** in $CD_3OD$ .......... 29

Figure S8 HMBC spectrum of **1** in $CD_3OD$ .......... 30

Figure S9 COSY spectrum of **1** in $CD_3OD$ .......... 31

Figure S10 ROESY spectrum of **1** in CD3OD .......... 32

Figure S11 HRMS spectrum of **1** .......... 33

Figure S12 $^{1}$H NMR spectrum of **2** in $CD_3OD$ .......... 34

Figure S13 $^{13}$C NMR spectrum of **2** in $CD_3OD$ .......... 35

Figure S14 HSQC spectrum of **2** in $CD_3OD$ .......... 36

Figure S15 HMBC spectrum of **2** in $CD_3OD$ .......... 37

Figure S16 COSY spectrum of **2** in $CD_3OD$ .......... 38
Figure S17 Roesy spectrum of **2** in $CD_3OD$ .......... 39
Figure S18 HRMS spectrum of **2** .......... 40
Figure S19 $^1$H NMR spectrum of **3** in $CD_3OD$ .......... 41
Figure S20 $^{13}$C NMR spectrum of **3** in $CD_3OD$ .......... 42
Figure S21 HSQC spectrum of **3** in $CD_3OD$ .......... 43
Figure S22 HMBC spectrum of **3** in $CD_3OD$ .......... 44
Figure S23 COSY spectrum of **3**in $CD_3OD$ .......... 45
Figure S24 Roesy spectrum of **3** in $CD_3OD$ .......... 46
Figure S25 HRMS spectrum of **3** .......... 47
Figure S26 $^1$H NMR spectrum of **4** in $CD_3OD$ .......... 48
Figure S27 $^{13}$C NMR spectrum of **4** in $CD_3OD$ .......... 49
Figure S28 HSQC spectrum of **4** in $CD_3OD$ .......... 50
Figure S29 HMBC spectrum of **4** in $CD_3OD$ .......... 51
Figure S30 COSY spectrum of **4** in $CD_3OD$ .......... 52
Figure S31 Roesy spectrum of **4** in $CD_3OD$ .......... 53
Figure S32 HRMS spectrum of **4** .......... 54
Figure S33 CD spectrum of **1-4** in MeOH .......... 55

# Quantum chemical calculation

The initial conformational analysis of the compound **1-4** were executed by employing Monte Carlo searching algorithm via the MMFF94 molecular mechanics force field, with the aid of the SPARTAN'16 program package, leading to afford a panel of relatively favored conformations in an energy range of 3 kcal/mol above the global minimum. The force field minimum energy conformers thus obtained were subsequently optimized by applying the density functional theory (DFT) with the M06-2X/def2-SVP level in vacuum, implemented in the Gaussian 09 software package. Harmonic vibrational frequencies were also performed to confirm no imaginary frequencies of the finally optimized conformers. These predominant conformers were subjected to theoretical calculation of ECD by utilizing Time-dependent density functional theory (TDDFT) calculations at the M06-2X/def2-SVP level in MeOH using the Polarizable Continuum Model (PCM) solvent model. The energies, oscillator strengths, and rotational strengths of each conformers were carried out with Gaussian 09 software package. The oretical calculations of ECD spectra for each conformer were then approximated by the Gaussian distribution. The final ECD spectrum of the individual conformers was summed up on the basis of Boltzmann-weighed population contribution by the SpecDisv1.71. Gauge Independent Atomic Orbital (GIAO) calculations of their $^{1}H$ and $^{13}C$ NMR chemical shifts using density functional theory (DFT) at the mPW1PW91/6-311+G(d,p) level with the PCM model in methanol. The calculated NMR data of these conformers were averaged according to the Boltzmann distribution theory and their relative Gibbs free energy. The $^{1}H$ and $^{13}C$ NMR chemical shifts for TMS were also calculated by the same procedures and used as the reference. After calculation, the experimental and calculated data were evaluated by the improved probability DP4+ method.

**1 NMR computational details for compound 1**

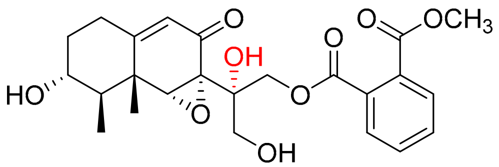


1a

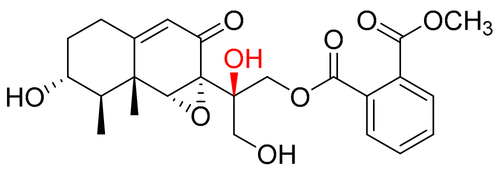


1b

Table S1. DP4+ analysis results of **1a** (Isomer 1) and **1b** (Isomer 2).

| Functional | | Solvent? | | Basis Set | | Type of Data | |
|---|---|---|---|---|---|---|---|
| mPW1PW91 | | PCM | | 6-311+G(d,p) | | Unscaled Shifts | |
| | | DP4+ | 100.00% | 0.00% | – | – | – |
| Nuclei | sp2? | xperimenta | Isomer 1 | Isomer 2 | Isomer 3 | Isomer 4 | Isomer 5 |
| C | x | 121.8 | 122.3 | 121.4 | | | |
| C | | 73.9 | 72.5 | 73.3 | | | |
| C | | 69.2 | 69.0 | 67.3 | | | |
| C | | 65.6 | 62.9 | 64.6 | | | |
| C | | 11.6 | 12.9 | 11.8 | | | |
| C | | 18.3 | 19.0 | 19.4 | | | |
| C | | 36.2 | 38.1 | 37.9 | | | |
| C | x | 169.5 | 166.7 | 166.3 | | | |
| C | x | 134.3 | 134.5 | 132.7 | | | |
| C | x | 129.7 | 131.0 | 131.1 | | | |
| C | x | 132.9 | 132.6 | 131.7 | | | |
| C | x | 132.8 | 131.6 | 132.5 | | | |
| C | x | 130.3 | 130.4 | 129.7 | | | |
| C | x | 132.3 | 132.4 | 134.5 | | | |
| C | x | 168.9 | 168.7 | 169.8 | | | |
| C | | 71.1 | 70.4 | 70.5 | | | |
| C | | 53.5 | 53.8 | 53.2 | | | |
| C | | 45.9 | 45.8 | 47.3 | | | |
| C | | 42.2 | 43.5 | 43.9 | | | |
| C | x | 166.5 | 171.4 | 171.8 | | | |
| C | | 31.6 | 32.8 | 33.2 | | | |
| C | | 63.9 | 61.3 | 61.3 | | | |
| C | | 62.9 | 61.4 | 60.6 | | | |
| C | x | 195.5 | 195.2 | 194.7 | | | |
| H | | 2.03 | 1.88 | 2.36 | | | |
| H | | 1.27 | 1.19 | 1.48 | | | |
| H | | 3.44 | 3.52 | 3.77 | | | |
| H | | 1.63 | 1.65 | 1.92 | | | |
| H | | 2.41 | 2.11 | 2.53 | | | |
| H | | 2.24 | 2.61 | 2.86 | | | |
| H | | 3.8 | 3.74 | 4.63 | | | |
| H | x | 5.61 | 5.57 | 6.17 | | | |
| H | | 4.64 | 4.37 | 5.21 | | | |
| H | | 4.59 | 4.78 | 4.37 | | | |
| H | | 4.19 | 3.63 | 5.18 | | | |
| H | | 3.76 | 3.45 | 3.80 | | | |
| H | | 1.16 | 1.34 | 1.91 | | | |
| H | | 0.64 | 1.23 | 1.14 | | | |
| H | x | 7.6 | 7.74 | 8.51 | | | |
| H | x | 7.61 | 7.64 | 8.33 | | | |
| H | x | 7.62 | 7.62 | 8.42 | | | |
| H | x | 7.77 | 7.93 | 8.38 | | | |
| H | | 3.85 | 3.84 | 4.66 | | | |

| Functional | Solvent? | | Basis Set | | Type of Data | |
|---|---|---|---|---|---|---|
| mPW1PW91 | PCM | | 6-311+G(d,p) | | Unscaled Shifts | |
| | Isomer 1 | Isomer 2 | Isomer 3 | Isomer 4 | Isomer 5 | Isomer 6 |
| sDP4+ (H data) | 94.72% | 5.28% | – | – | – | – |
| sDP4+ (C data) | 99.27% | 0.73% | – | – | – | – |
| sDP4+ (all data) | 99.96% | 0.04% | – | – | – | – |
| uDP4+ (H data) | 100.00% | 0.00% | – | – | – | – |
| uDP4+ (C data) | 68.40% | 31.60% | – | – | – | – |
| uDP4+ (all data) | 100.00% | 0.00% | – | – | – | – |
| DP4+ (H data) | 100.00% | 0.00% | – | – | – | – |
| DP4+ (C data) | 99.66% | 0.34% | – | – | – | – |
| DP4+ (all data) | 100.00% | 0.00% | – | – | – | – |

Table S2. Experimental and calculated $^{13}C$ NMR chemical shifts of **1a** and **1b**.

| Num. | Exp. | **1a** | **1b** | Num. | Exp. | **1a** | **1b** |
|---|---|---|---|---|---|---|---|
| 1 | 121.8 | 122.3 | 121.4 | 15 | 168.9 | 168.7 | 169.8 |
| 2 | 73.9 | 72.5 | 73.3 | 16 | 71.1 | 70.4 | 70.5 |
| 3 | 69.2 | 69.0 | 67.3 | 17 | 53.5 | 53.8 | 53.2 |
| 4 | 65.6 | 62.9 | 64.6 | 18 | 45.9 | 45.8 | 47.3 |
| 5 | 11.6 | 12.9 | 11.8 | 19 | 42.2 | 43.5 | 43.9 |
| 6 | 18.3 | 19.0 | 19.4 | 20 | 166.5 | 171.4 | 171.8 |
| 7 | 36.2 | 38.1 | 37.9 | 21 | 31.6 | 32.8 | 33.2 |
| 8 | 169.5 | 166.7 | 166.3 | 22 | 63.9 | 61.3 | 61.3 |
| 9 | 134.3 | 134.5 | 132.7 | 23 | 62.9 | 61.4 | 60.6 |
| 10 | 129.7 | 131.0 | 131.1 | 24 | 195.5 | 195.2 | 194.7 |
| 11 | 132.9 | 132.6 | 131.7 | $R^2$ | | **0.9991** | 0.9988 |
| 12 | 132.8 | 131.6 | 132.5 | MAE | | 2.6428 | 3.3397 |
| 13 | 130.3 | 130.4 | 129.7 | RMSD | | 1.6257 | 1.8275 |
| 14 | 132.3 | 132.4 | 134.5 | | | | |

## 2 ECD computational details of compound 1

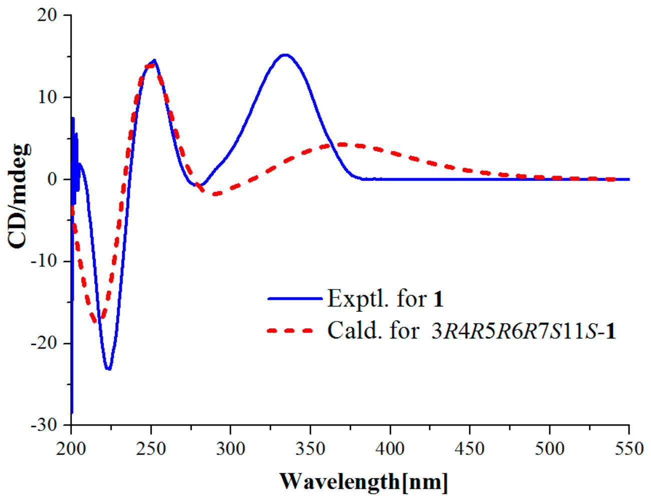


Figure S1. Experimental ECD spectra and calculated ECD spectra of **1**.

Table S3. Important thermodynamic parameters of the M06-2X/def2-SVP optimized conformers of **1a** in the gas phase

| Conformers | E[a](Hartree) | C[b](Hartree) | G[c](kcal/mol) |
|---|---|---|---|
| **1a**_1 | -1606.598579 | 0.442916 | -1007867.497250 |
| **1a**_2 | -1606.598449 | 0.442945 | -1007867.397478 |
| **1a**_3 | -1606.59598 | 0.442534 | -1007866.106076 |
| **1a**_4 | -1606.59598 | 0.442543 | -1007866.100366 |
| **1a**_5 | -1606.595358 | 0.442541 | -1007865.711126 |

[a]Electronic energy; [b]Thermal correction to Gibbs free energy ; [c]Gibbs free energy (E + C).

Table S4. Conformational analysis of the M06-2X/def2-SVP optimized conformers of **1a** in the gas phase (T=298.15 K)

| Conformers | ΔG (kcal/mol)[a] | Population[b] |
|---|---|---|
| **1a**_1 | 0.000000 | 47.99% |
| **1a**_2 | 0.099773 | 40.55% |
| **1a**_3 | 1.391802 | 4.57% |
| **1a**_4 | 1.397449 | 4.53% |
| **1a**_5 | 1.786501 | 2.35% |

[a]The relative Gibbs free energy; [b]The Boltzmann distribution of each conformer.

**3 ECD computational details of compound 2**

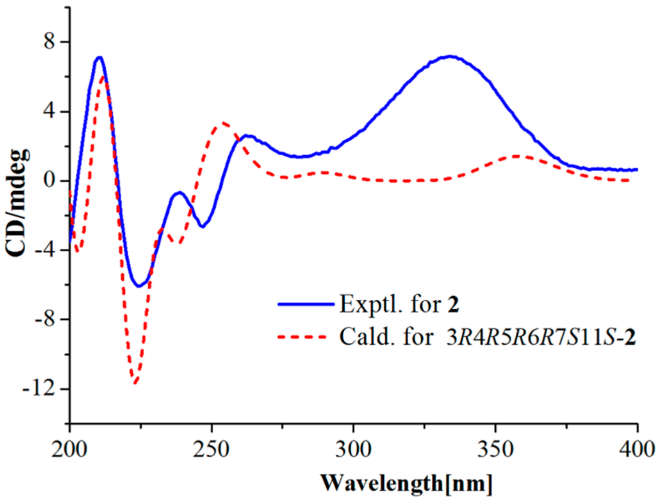


Figure S2. Experimental ECD spectra and calculated ECD spectra of **2**.

Table S5. Important thermodynamic parameters of the M06-2X/def2-SVP optimized conformers of **2** in the gas phase

| Conformers | E[a](Hartree) | C[b](Hartree) | G[c](kcal/mol) |
|---|---|---|---|
| **2**_1 | -1454.115283 | 0.404613 | -912207.806683 |
| **2**_2 | -1454.115638 | 0.405111 | -912207.716573 |
| **2**_3 | -1454.115283 | 0.404840 | -912207.663800 |
| **2**_4 | -1454.114912 | 0.404576 | -912207.596720 |
| **2**_5 | -1454.114626 | 0.404514 | -912207.456536 |
| **2**_6 | -1454.114230 | 0.404397 | -912207.281149 |
| **2**_7 | -1454.114175 | 0.404669 | -912207.076395 |

[a]Electronic energy; [b]Thermal correction to Gibbs free energy ; [c]Gibbs free energy (E + C).

Table S6. Conformational analysis of the M06-2X/def2-SVP optimized conformers of **2** in the gas phase (T=298.15 K)

| Conformers | ΔG (kcal/mol)[a] | Population[b] |
|---|---|---|
| **2**_1 | 0.000000 | 21.73% |
| **2**_2 | 0.090110 | 18.66% |
| **2**_3 | 0.142883 | 17.07% |
| **2**_4 | 0.209963 | 15.24% |
| **2**_5 | 0.350147 | 12.03% |
| **2**_6 | 0.525534 | 8.94% |
| **2**_7 | 0.730288 | 6.33% |

[a]The relative Gibbs free energy; [b]The Boltzmann distribution of each conformer.

**4 ECD computational details of compound 3**

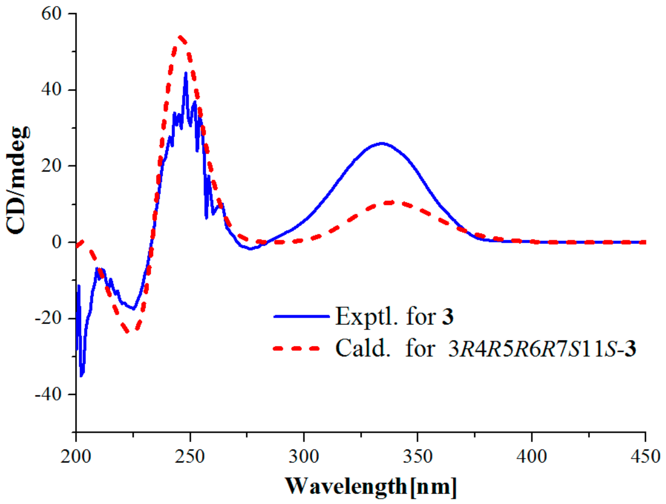


Figure S3. Experimental ECD spectra and calculated ECD spectra of **3**.

Table S7. Important thermodynamic parameters of the M06-2X/def2-SVP optimized conformers of **3** in the gas phase

| Conformers | E[a](Hartree) | C[b](Hartree) | G[c](kcal/mol) |
|---|---|---|---|
| **3**_1 | -1454.121572 | 0.405281 | -912211.333689 |
| **3**_2 | -1454.121054 | 0.405538 | -912210.847311 |
| **3**_3 | -1454.117371 | 0.405032 | -912208.853922 |
| **3**_4 | -1454.117396 | 0.405106 | -912208.823237 |
| **3**_5 | -1454.116985 | 0.404891 | -912208.700310 |

[a]Electronic energy; [b]Thermal correction to Gibbs free energy ; [c]Gibbs free energy (E + C).

Table S8. Conformational analysis of the M06-2X/def2-SVP optimized conformers of **3** in the gas phase (T=298.15 K)

| Conformers | ΔG (kcal/mol)[a] | Population[b] |
|---|---|---|
| **3**_1 | 0.000000 | 67.52% |
| **3**_2 | 0.486378 | 29.69% |
| **3**_3 | 2.479767 | 1.02% |
| **3**_4 | 2.510452 | 0.97% |
| **3**_5 | 2.633379 | 0.79% |

[a]The relative Gibbs free energy; [b]The Boltzmann distribution of each conformer.

**5 ECD computational details of compound 4**

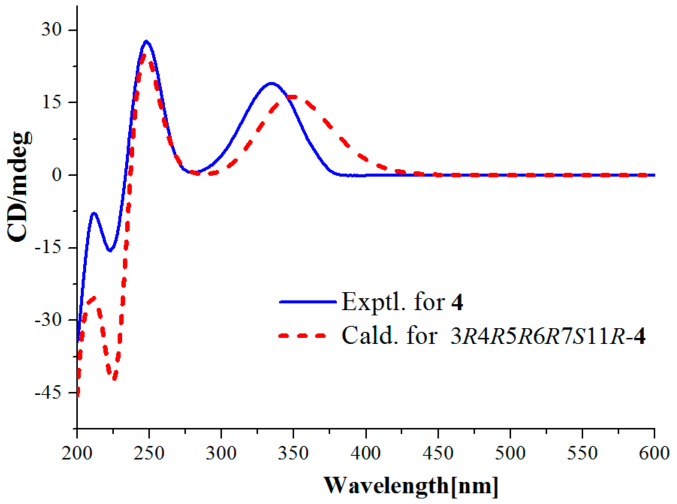


Figure S4. Experimental ECD spectra and calculated ECD spectra of **4**.

Table S9. Important thermodynamic parameters of the M06-2X/def2-SVP optimized conformers of **4** in the gas phase

| Conformers | E[a](Hartree) | C[b](Hartree) | G[c](kcal/mol) |
|---|---|---|---|
| **4**_1 | -1111.065458 | 0.324859 | -696993.057969 |
| **4**_2 | -1111.065133 | 0.324707 | -696992.949599 |
| **4**_3 | -1111.061775 | 0.324333 | -696991.077318 |
| **4**_4 | -1111.061525 | 0.324398 | -696990.879529 |
| **4**_5 | -1111.061126 | 0.325496 | -696989.939781 |
| **4**_6 | -1111.058664 | 0.323275 | -696989.788866 |

[a]Electronic energy; [b]Thermal correction to Gibbs free energy ; [c]Gibbs free energy (E + C).

Table S10. Conformational analysis of the M06-2X/def2-SVP optimized conformers of **4** in the gas phase (T=298.15 K)

| Conformers | ΔG (kcal/mol)[a] | Population[b] |
|---|---|---|
| **4**_1 | 0.000000 | 52.56% |
| **4**_2 | 0.108370 | 43.77% |
| **4**_3 | 1.980650 | 1.85% |
| **4**_4 | 2.178439 | 1.33% |
| **4**_5 | 3.118188 | 0.27% |
| **4**_6 | 3.269102 | 0.21% |

[a]The relative Gibbs free energy; [b]The Boltzmann distribution of each conformer.

Table S11. Cartesian coordinates for the low-energy optimized conformers of **1-4** at M06-2X/def2-SVP level.

| Conformer **1a**_1 | | | | | | | |
|---|---|---|---|---|---|---|---|
| C | 3.813507 | -2.167609 | 1.672918 | C | -0.17331 | -1.812801 | -2.600849 |
| C | 4.137678 | -2.014072 | 0.194551 | O | 1.455004 | 3.825742 | -1.175527 |
| C | 3.075336 | -1.184588 | -0.530326 | H | 4.596367 | -2.763237 | 2.170226 |
| C | 2.820246 | 0.204901 | 0.136454 | H | 2.866455 | -2.723197 | 1.762109 |
| C | 2.648075 | 0.034432 | 1.6392 | H | 5.113317 | -1.492316 | 0.096363 |
| C | 3.690018 | -0.797845 | 2.335704 | H | 2.12925 | -1.745179 | -0.417978 |
| C | 1.566643 | 0.765918 | -0.502885 | H | 3.444206 | -0.890163 | 3.401706 |
| C | 0.478136 | 1.419105 | 0.247573 | H | 4.663476 | -0.280695 | 2.272913 |
| C | 0.651241 | 1.513818 | 1.743047 | H | 1.670516 | 1.097119 | -1.540589 |
| C | 1.658893 | 0.617246 | 2.336711 | H | 1.608595 | 0.523532 | 3.423783 |
| O | 0.021852 | 2.303343 | 2.413142 | H | -2.475791 | 3.015593 | -0.565427 |
| O | 0.362169 | 0.091133 | -0.235474 | H | -1.786789 | 3.06384 | 1.09709 |
| C | -0.396858 | 2.453321 | -0.46871 | H | 0.526931 | 4.086188 | 0.616187 |
| C | -1.793077 | 2.524202 | 0.141707 | H | -0.449447 | 4.575477 | -0.796653 |
| C | 0.275049 | 3.828088 | -0.421287 | H | -1.007365 | 1.311001 | -1.940317 |
| O | -0.474561 | 2.120783 | -1.83399 | H | 3.446084 | -2.108574 | -2.440647 |
| C | 3.417668 | -1.097272 | -2.017393 | H | 4.410701 | -0.647965 | -2.169671 |
| C | 3.950684 | 1.216986 | -0.123395 | H | 2.685641 | -0.504348 | -2.582328 |
| O | 4.188841 | -3.265708 | -0.451582 | H | 3.707447 | 2.176929 | 0.353401 |
| O | -2.213434 | 1.182439 | 0.365349 | H | 4.917986 | 0.865846 | 0.262047 |
| C | -3.512193 | 0.898127 | 0.26672 | H | 4.059163 | 1.403805 | -1.200864 |
| C | -3.754861 | -0.579024 | 0.395461 | H | 4.860331 | -3.804964 | -0.020848 |
| O | -4.381824 | 1.717243 | 0.169166 | H | -5.372392 | -0.201376 | 1.746442 |
| C | -4.775087 | -0.973274 | 1.259189 | H | -5.806638 | -2.623263 | 2.18695 |
| C | -5.010778 | -2.325553 | 1.503002 | H | -4.403649 | -4.350728 | 1.067834 |
| C | -4.226528 | -3.291569 | 0.877131 | H | -2.621225 | -3.651354 | -0.528393 |

| | | | | | | | |
|---|---|---|---|---|---|---|---|
| C | -3.222716 | -2.905545 | -0.009319 | H | 0.289559 | -0.831738 | -2.436532 |
| C | -2.987629 | -1.553027 | -0.266117 | H | 0.580082 | -2.60603 | -2.566925 |
| C | -2.022015 | -1.167798 | -1.34165 | H | -0.690249 | -1.810129 | -3.570369 |
| O | -2.117734 | -0.160569 | -2.002835 | H | 1.208415 | 3.47908 | -2.044112 |
| O | -1.092776 | -2.089426 | -1.553252 | | | | |

| Conformer **1a**_2 | | | | | | | |
|---|---|---|---|---|---|---|---|
| C | 3.820279 | -2.168969 | 1.681244 | C | -0.209963 | -1.731348 | -2.676541 |
| C | 4.122762 | -2.039706 | 0.195929 | O | 1.457617 | 3.835284 | -1.156868 |
| C | 3.0617 | -1.195807 | -0.528184 | H | 4.607445 | -2.767514 | 2.161345 |
| C | 2.818644 | 0.199691 | 0.132106 | H | 2.867506 | -2.714835 | 1.801483 |
| C | 2.651941 | 0.033026 | 1.635728 | H | 5.105157 | -1.550849 | 0.073665 |
| C | 3.700845 | -0.792119 | 2.330137 | H | 2.10193 | -1.736972 | -0.408855 |
| C | 1.568585 | 0.770349 | -0.506282 | H | 3.465579 | -0.873756 | 3.399414 |
| C | 0.478392 | 1.417653 | 0.246594 | H | 4.674072 | -0.277135 | 2.252756 |
| C | 0.647227 | 1.501876 | 1.743597 | H | 1.676038 | 1.108721 | -1.541486 |
| C | 1.660311 | 0.610119 | 2.334567 | H | 1.613341 | 0.517306 | 3.421869 |
| O | 0.010539 | 2.28241 | 2.41713 | H | -2.478628 | 3.007567 | -0.558723 |
| O | 0.362267 | 0.09353 | -0.247693 | H | -1.78741 | 3.070854 | 1.101975 |
| C | -0.395282 | 2.458411 | -0.461789 | H | 0.529798 | 4.08137 | 0.636793 |
| C | -1.79154 | 2.524781 | 0.150341 | H | -0.446398 | 4.583297 | -0.771501 |
| C | 0.277691 | 3.832192 | -0.402816 | H | -1.021084 | 1.34014 | -1.945441 |
| O | -0.471703 | 2.137065 | -1.830233 | H | 3.452141 | -2.130845 | -2.432438 |
| C | 3.396962 | -1.117078 | -2.016807 | H | 4.38031 | -0.649963 | -2.17539 |
| C | 3.959951 | 1.197418 | -0.134732 | H | 2.651339 | -0.547014 | -2.587317 |
| O | 4.265344 | -3.304851 | -0.407372 | H | 3.736967 | 2.156454 | 0.353664 |
| O | -2.203215 | 1.181868 | 0.382999 | H | 4.927172 | 0.829099 | 0.234473 |
| C | -3.501573 | 0.891251 | 0.301666 | H | 4.056959 | 1.39249 | -1.211743 |
| C | -3.732049 | -0.589159 | 0.417858 | H | 3.439567 | -3.78711 | -0.276664 |
| O | -4.377393 | 1.705562 | 0.223726 | H | -5.321062 | -0.244075 | 1.810941 |
| C | -4.729614 | -1.003802 | 1.298066 | H | -5.728781 | -2.675492 | 2.222408 |
| C | -4.950673 | -2.361491 | 1.525464 | H | -4.34206 | -4.375487 | 1.043344 |
| C | -4.175212 | -3.312331 | 0.866166 | H | -2.601359 | -3.639248 | -0.582146 |
| C | -3.194308 | -2.905597 | -0.036528 | H | 0.256758 | -0.756118 | -2.488512 |
| C | -2.97358 | -1.547571 | -0.276074 | H | 0.544318 | -2.524357 | -2.691284 |
| C | -2.034916 | -1.136084 | -1.364631 | H | -0.749865 | -1.694715 | -3.632622 |
| O | -2.150754 | -0.116928 | -2.00374 | H | 1.21045 | 3.501397 | -2.030203 |
| O | -1.102663 | -2.046118 | -1.616734 | | | | |

| Conformer **1a**_3 | | | | | | | |
|---|---|---|---|---|---|---|---|
| C | -4.23733 | -2.292152 | -0.640628 | C | 4.074632 | 1.447555 | 2.375053 |
| C | -3.92598 | -2.209531 | 0.848999 | O | -1.28349 | 3.917138 | 0.807963 |

| | | | | | | | |
|---|---|---|---|---|---|---|---|
| C | -2.65116 | -1.388793 | 1.095458 | H | -5.163807 | -2.865055 | -0.788559 |
| C | -2.740411 | 0.046307 | 0.484173 | H | -3.42118 | -2.85298 | -1.129942 |
| C | -3.120646 | -0.075407 | -0.983254 | H | -4.770171 | -1.728493 | 1.37277 |
| C | -4.343284 | -0.905199 | -1.272686 | H | -1.845896 | -1.887581 | 0.522425 |
| C | -1.380402 | 0.684872 | 0.670881 | H | -4.497721 | -0.976328 | -2.357531 |
| C | -0.646076 | 1.377832 | -0.40422 | H | -5.227439 | -0.391356 | -0.857935 |
| C | -1.319132 | 1.454343 | -1.755614 | H | -1.160787 | 1.014965 | 1.691086 |
| C | -2.447125 | 0.532105 | -1.97362 | H | -2.779993 | 0.453433 | -3.010797 |
| O | -0.98551 | 2.265767 | -2.590224 | H | 2.3376 | 3.10051 | -0.726134 |
| O | -0.300421 | 0.064903 | 0.020512 | H | 1.309981 | 2.394307 | -2.035705 |
| C | 0.401069 | 2.436633 | -0.051149 | H | -0.591169 | 4.062455 | -1.098552 |
| C | 1.608289 | 2.321212 | -0.983069 | H | 0.586152 | 4.558392 | 0.155259 |
| C | -0.214605 | 3.836964 | -0.094475 | H | 1.106862 | 1.351268 | 1.433433 |
| O | 0.819912 | 2.271689 | 1.284365 | H | -2.967548 | -0.8513 | 3.192623 |
| C | -2.251786 | -1.40772 | 2.569245 | H | -1.246834 | -0.991391 | 2.7231 |
| C | -3.755192 | 0.960535 | 1.193659 | H | -2.248119 | -2.444006 | 2.928066 |
| O | -3.833104 | -3.497725 | 1.411632 | H | -3.696505 | 1.971238 | 0.763503 |
| O | 2.271345 | 1.078118 | -0.740665 | H | -4.786811 | 0.597433 | 1.097684 |
| C | 1.951982 | 0.028141 | -1.513044 | H | -3.522492 | 1.040046 | 2.264822 |
| C | 2.631222 | -1.227451 | -1.061797 | H | -3.105047 | -3.957068 | 0.974907 |
| O | 1.242568 | 0.09483 | -2.477273 | H | 2.229865 | -2.129229 | -2.956734 |
| C | 2.698479 | -2.274765 | -1.982645 | H | 3.406728 | -4.269823 | -2.385281 |
| C | 3.354237 | -3.458533 | -1.658427 | H | 4.470049 | -4.522507 | -0.14637 |
| C | 3.948669 | -3.599949 | -0.405308 | H | 4.329935 | -2.676831 | 1.50997 |
| C | 3.87556 | -2.56402 | 0.524302 | H | 5.044192 | 1.953239 | 2.357956 |
| C | 3.209762 | -1.379999 | 0.206454 | H | 3.270043 | 2.12978 | 2.066553 |
| C | 3.06442 | -0.329962 | 1.26969 | H | 3.853628 | 1.068972 | 3.381769 |
| O | 2.059479 | -0.168381 | 1.917283 | H | -0.937103 | 3.604184 | 1.654711 |
| O | 4.173276 | 0.369364 | 1.448287 | | | | |

| Conformer **1a**_4 | | | | | | | |
|---|---|---|---|---|---|---|---|
| C | 4.236731 | 2.292667 | -0.640771 | C | -4.074409 | -1.448108 | 2.374917 |
| C | 3.926052 | 2.209587 | 0.84897 | O | 1.283696 | -3.917074 | 0.807576 |
| C | 2.651519 | 1.388515 | 1.09582 | H | 5.162951 | 2.86592 | -0.788943 |
| C | 2.740616 | -0.046364 | 0.483991 | H | 3.420195 | 2.853313 | -1.129641 |
| C | 3.120476 | 0.075787 | -0.983516 | H | 4.770592 | 1.728573 | 1.372202 |
| C | 4.3429 | 0.905852 | -1.273062 | H | 1.845851 | 1.887385 | 0.52342 |
| C | 1.380633 | -0.684979 | 0.670802 | H | 4.497145 | 0.977149 | -2.357921 |
| C | 0.646136 | -1.37774 | -0.404321 | H | 5.227222 | 0.392133 | -0.858507 |
| C | 1.319072 | -1.454112 | -1.755771 | H | 1.16116 | -1.015281 | 1.690966 |
| C | 2.446837 | -0.531619 | -1.973866 | H | 2.779487 | -0.452677 | -3.011091 |
| O | 0.98554 | -2.265607 | -2.590351 | H | -2.337541 | -3.100392 | -0.726332 |

| | | | | | | | |
|---|---|---|---|---|---|---|---|
| O | 0.300574 | -0.064887 | 0.02068 | H | -1.309938 | -2.394034 | -2.035838 |
| C | -0.400995 | -2.436567 | -0.051289 | H | 0.591063 | -4.062405 | -1.09882 |
| C | -1.608235 | -2.321067 | -0.983191 | H | -0.586049 | -4.558327 | 0.155171 |
| C | 0.21467 | -3.836897 | -0.094682 | H | -1.106922 | -1.351302 | 1.433306 |
| O | -0.819858 | -2.271692 | 1.284226 | H | 1.248462 | 0.989925 | 2.724303 |
| C | 2.253068 | 1.406869 | 2.569861 | H | 2.249054 | 2.443049 | 2.928976 |
| C | 3.755586 | -0.960812 | 1.19291 | H | 2.969573 | 0.850705 | 3.192612 |
| O | 3.833145 | 3.497599 | 1.412016 | H | 3.697033 | -1.971307 | 0.762259 |
| O | -2.271308 | -1.078004 | -0.740639 | H | 4.787132 | -0.597465 | 1.097055 |
| C | -1.951997 | -0.027924 | -1.512887 | H | 3.522973 | -1.040918 | 2.264048 |
| C | -2.631431 | 1.227539 | -1.061579 | H | 3.104219 | 3.956587 | 0.97636 |
| O | -1.242391 | -0.094425 | -2.476995 | H | -2.230026 | 2.129564 | -2.956404 |
| C | -2.698744 | 2.274925 | -1.98234 | H | -3.407238 | 4.269922 | -2.384862 |
| C | -3.35469 | 3.458582 | -1.658069 | H | -4.470776 | 4.52226 | -0.146007 |
| C | -3.949251 | 3.5998 | -0.404995 | H | -4.330577 | 2.676462 | 1.510161 |
| C | -3.876093 | 2.563789 | 0.524526 | H | -5.043947 | -1.95383 | 2.357978 |
| C | -3.210112 | 1.379893 | 0.206638 | H | -3.853148 | -1.069636 | 3.381616 |
| C | -3.064593 | 0.329729 | 1.26973 | H | -3.26984 | -2.130229 | 2.066135 |
| O | -2.059581 | 0.16825 | 1.917244 | H | 0.937448 | -3.604169 | 1.654399 |
| O | -4.17332 | -0.369789 | 1.448298 | | | | |

| Conformer **1a**_5 | | | | | | | |
|---|---|---|---|---|---|---|---|
| C | -4.23545 | -2.273248 | -0.649264 | C | 4.094781 | 1.421301 | 2.369255 |
| C | -3.950828 | -2.167586 | 0.843825 | O | -1.26174 | 3.915517 | 0.816606 |
| C | -2.667083 | -1.373722 | 1.094699 | H | -5.170478 | -2.832433 | -0.817672 |
| C | -2.734453 | 0.057835 | 0.473225 | H | -3.416875 | -2.85361 | -1.103723 |
| C | -3.103304 | -0.066148 | -0.997079 | H | -4.794144 | -1.64275 | 1.340027 |
| C | -4.325898 | -0.892651 | -1.296692 | H | -1.871136 | -1.897896 | 0.536884 |
| C | -1.368779 | 0.682424 | 0.665959 | H | -4.464251 | -0.973835 | -2.382978 |
| C | -0.628679 | 1.37813 | -0.403589 | H | -5.212798 | -0.368218 | -0.900754 |
| C | -1.296295 | 1.462092 | -1.756915 | H | -1.150607 | 1.008301 | 1.687736 |
| C | -2.422891 | 0.540285 | -1.983261 | H | -2.748151 | 0.461094 | -3.022796 |
| O | -0.960046 | 2.277712 | -2.586511 | H | 2.362692 | 3.090442 | -0.707666 |
| O | -0.289819 | 0.06184 | 0.016224 | H | 1.335692 | 2.396755 | -2.024456 |
| C | 0.420539 | 2.432317 | -0.042847 | H | -0.563382 | 4.064978 | -1.087625 |
| C | 1.630347 | 2.316187 | -0.971305 | H | 0.611794 | 4.552707 | 0.171565 |
| C | -0.190513 | 3.834763 | -0.083275 | H | 1.117878 | 1.340147 | 1.44121 |
| O | 0.834246 | 2.261911 | 1.293526 | H | -2.251569 | -2.43143 | 2.917553 |
| C | -2.28369 | -1.391428 | 2.572822 | H | -3.019025 | -0.856702 | 3.193187 |
| C | -3.739739 | 0.994242 | 1.168185 | H | -1.291299 | -0.950203 | 2.737879 |
| O | -3.774529 | -3.445339 | 1.412028 | H | -3.643454 | 2.006715 | 0.748892 |
| O | 2.285553 | 1.068349 | -0.733395 | H | -4.778771 | 0.661319 | 1.046261 |

| | | | | | | | |
|---|---|---|---|---|---|---|---|
| C | 1.962784 | 0.02429 | -1.51301 | H | -3.528222 | 1.057025 | 2.244992 |
| C | 2.625077 | -1.239845 | -1.061468 | H | -4.572358 | -3.960947 | 1.254883 |
| O | 1.261348 | 0.102524 | -2.482242 | H | 2.21493 | -2.135383 | -2.957399 |
| C | 2.679238 | -2.287862 | -1.982354 | H | 3.356379 | -4.294041 | -2.38261 |
| C | 3.315186 | -3.481736 | -1.656203 | H | 4.40693 | -4.564118 | -0.139756 |
| C | 3.902384 | -3.632874 | -0.400877 | H | 4.289903 | -2.71709 | 1.516501 |
| C | 3.84219 | -2.596223 | 0.528755 | H | 5.06708 | 1.921497 | 2.345174 |
| C | 3.196836 | -1.401583 | 0.208694 | H | 3.291984 | 2.107655 | 2.065131 |
| C | 3.065604 | -0.349758 | 1.271947 | H | 3.878031 | 1.045854 | 3.378099 |
| O | 2.066122 | -0.179316 | 1.925447 | H | -0.919696 | 3.593492 | 1.661797 |
| O | 4.181205 | 0.341236 | 1.443856 | | | | |

| Conformer **2**_1 | | | | | | | |
|---|---|---|---|---|---|---|---|
| O | 0.852007 | -2.795104 | -1.238511 | C | 3.868586 | -1.091705 | -0.393751 |
| C | 1.559303 | -1.876972 | -0.879395 | C | 3.021285 | -1.917002 | -1.031824 |
| C | 0.982271 | -0.640265 | -0.234222 | H | -0.374286 | -2.684259 | 0.962963 |
| C | -0.480714 | -0.655608 | 0.213174 | H | -1.77047 | -1.727766 | 1.540324 |
| C | -0.689013 | -1.693638 | 1.316238 | H | -0.167474 | -0.463734 | 2.682705 |
| O | 0.072966 | -1.369869 | 2.447713 | H | -0.836841 | 1.258444 | 0.118596 |
| O | -0.796168 | 0.580458 | 0.811044 | H | -1.086256 | -0.250371 | -1.826994 |
| C | -1.386695 | -0.909474 | -0.999151 | H | -1.316393 | -1.955022 | -1.313613 |
| O | -2.750175 | -0.668122 | -0.668872 | H | -4.631901 | 2.811236 | -0.851328 |
| C | -3.207189 | 0.583088 | -0.798821 | H | -7.063695 | 3.088313 | -0.26855 |
| C | -4.651338 | 0.704451 | -0.444507 | H | -8.395045 | 1.102061 | 0.456918 |
| C | -5.240593 | 1.966077 | -0.531074 | H | -6.996678 | -2.097245 | 0.701176 |
| C | -6.588812 | 2.108342 | -0.206175 | H | -4.915121 | -1.386158 | 0.019273 |
| C | -7.339674 | 1.009923 | 0.198805 | H | 1.493778 | 0.883752 | 1.230884 |
| C | -6.743644 | -0.254322 | 0.283431 | H | 3.080375 | -1.307245 | 2.218318 |
| O | -7.518618 | -1.287521 | 0.681424 | H | 4.731188 | -0.622153 | 2.180902 |
| C | -5.39537 | -0.407821 | -0.039698 | H | 3.353308 | 0.38026 | 2.686701 |
| O | -2.526507 | 1.506013 | -1.171404 | H | 3.771749 | 1.582583 | -0.875393 |
| C | 1.922095 | 0.278689 | 0.427281 | H | 4.320564 | 3.4229 | 0.688881 |
| O | 1.397093 | 0.574372 | -0.846025 | H | 4.156664 | 2.348515 | 2.081449 |
| C | 3.411452 | 0.02259 | 0.534578 | H | 2.729096 | 2.7366 | 1.082069 |
| C | 3.671052 | -0.408252 | 1.990462 | H | 6.072401 | 0.933127 | 1.017202 |
| C | 4.140405 | 1.34725 | 0.141858 | H | 5.971112 | 2.625145 | -1.225182 |
| C | 3.806486 | 2.525791 | 1.053753 | H | 7.110429 | -0.04785 | -0.999474 |
| C | 5.662856 | 1.17648 | 0.021292 | H | 5.666901 | 0.3466 | -1.956145 |
| O | 6.290093 | 2.382071 | -0.347166 | H | 5.575409 | -2.0533 | -1.26464 |
| C | 6.017526 | 0.058075 | -0.949278 | H | 5.77663 | -1.580338 | 0.427329 |
| C | 5.357096 | -1.257087 | -0.540833 | H | 3.394806 | -2.734142 | -1.652858 |

| Conformer **2**_2 | | | | | | | |
|---|---|---|---|---|---|---|---|
| O | 1.069423 | -2.910431 | -1.186459 | C | 3.942784 | -0.947313 | -0.407844 |
| C | 1.701176 | -1.931839 | -0.847314 | C | 3.158953 | -1.84892 | -1.02338 |
| C | 1.03099 | -0.742237 | -0.203552 | H | -0.123611 | -2.876962 | 1.045789 |
| C | -0.417499 | -0.877298 | 0.26988 | H | -1.587324 | -2.036465 | 1.63515 |
| C | -0.517027 | -1.911917 | 1.39108 | H | -0.083937 | -0.622575 | 2.733552 |
| O | 0.233555 | -1.505946 | 2.5038 | H | -0.924815 | 0.999886 | 0.15331 |
| O | -0.828455 | 0.337059 | 0.854731 | H | -1.088621 | -0.561428 | -1.766327 |
| C | -1.319807 | -1.226776 | -0.921403 | H | -1.171249 | -2.268893 | -1.218714 |
| O | -2.692104 | -1.090934 | -0.569036 | H | -4.804057 | -1.96401 | 0.202168 |
| C | -3.253081 | 0.11301 | -0.711931 | H | -7.226867 | -1.826465 | 0.818469 |
| C | -4.69519 | 0.121784 | -0.328949 | H | -8.441518 | 0.346046 | 0.61855 |
| C | -5.349597 | -1.025254 | 0.122446 | H | -6.873124 | 3.266118 | -0.511713 |
| C | -6.699766 | -0.939944 | 0.463427 | H | -4.827942 | 2.212707 | -0.800161 |
| C | -7.386375 | 0.264409 | 0.355955 | H | 1.434137 | 0.834721 | 1.2387 |
| C | -6.723839 | 1.4116 | -0.099165 | H | 4.805799 | -0.370259 | 2.145747 |
| O | -7.436998 | 2.555978 | -0.185521 | H | 3.354248 | 0.514902 | 2.664268 |
| C | -5.374988 | 1.337505 | -0.441801 | H | 3.221947 | -1.196021 | 2.221022 |
| O | -2.660673 | 1.083447 | -1.116678 | H | 3.607264 | 1.700652 | -0.919201 |
| C | 1.899468 | 0.260485 | 0.433056 | H | 2.50085 | 2.790331 | 1.03759 |
| O | 1.329776 | 0.497137 | -0.833647 | H | 3.968105 | 2.535068 | 2.02214 |
| C | 3.406962 | 0.134934 | 0.516106 | H | 4.024505 | 3.602075 | 0.615193 |
| C | 3.727968 | -0.252168 | 1.971886 | H | 5.986349 | 1.278532 | 0.940505 |
| C | 4.01154 | 1.512198 | 0.094262 | H | 5.703946 | 2.923495 | -1.321131 |
| C | 3.591693 | 2.669897 | 0.997262 | H | 7.072293 | 0.363256 | -1.081672 |
| C | 5.540978 | 1.471995 | -0.050905 | H | 5.584701 | 0.618787 | -2.017924 |
| O | 6.055399 | 2.722119 | -0.444964 | H | 5.711588 | -1.770273 | -1.296841 |
| C | 5.975276 | 0.375238 | -1.01385 | H | 5.899086 | -1.259806 | 0.385756 |
| C | 5.437398 | -0.986457 | -0.578541 | H | 3.590762 | -2.638427 | -1.642381 |

| Conformer **2**_3 | | | | | | | |
|---|---|---|---|---|---|---|---|
| O | 1.066391 | -2.925277 | -1.157341 | C | 3.939088 | -0.9483 | -0.41535 |
| C | 1.697671 | -1.940802 | -0.834311 | C | 3.154069 | -1.856128 | -1.01996 |
| C | 1.029305 | -0.744098 | -0.202652 | H | -0.128069 | -2.864562 | 1.069669 |
| C | -0.418904 | -0.872712 | 0.273277 | H | -1.590407 | -2.015598 | 1.650633 |
| C | -0.519919 | -1.895452 | 1.405083 | H | -0.081606 | -0.590846 | 2.73076 |
| O | 0.231278 | -1.479095 | 2.513678 | H | -0.917591 | 1.00548 | 0.138293 |
| O | -0.828707 | 0.347952 | 0.845729 | H | -1.091248 | -0.575089 | -1.765035 |
| C | -1.321884 | -1.23304 | -0.914193 | H | -1.173251 | -2.277793 | -1.202148 |

| | | | | | | | |
|---|---|---|---|---|---|---|---|
| O | -2.693928 | -1.094267 | -0.561863 | H | -4.80432 | -1.95995 | 0.221994 |
| C | -3.255288 | 0.10822 | -0.715821 | H | -7.2259 | -1.816452 | 0.8422 |
| C | -4.696624 | 0.12058 | -0.329246 | H | -8.440926 | 0.354149 | 0.624252 |
| C | -5.350092 | -1.022011 | 0.13457 | H | -6.874267 | 3.263196 | -0.536806 |
| C | -6.699517 | -0.933374 | 0.477621 | H | -4.830235 | 2.20676 | -0.820229 |
| C | -7.386352 | 0.269956 | 0.360173 | H | 1.43533 | 0.84833 | 1.220802 |
| C | -6.724705 | 1.412661 | -0.107244 | H | 4.794 | -0.394483 | 2.139091 |
| O | -7.437845 | 2.556402 | -0.202882 | H | 3.362184 | 0.524807 | 2.653519 |
| C | -5.376627 | 1.335159 | -0.452191 | H | 3.192562 | -1.185064 | 2.216638 |
| O | -2.664621 | 1.074468 | -1.13232 | H | 3.622148 | 1.71363 | -0.925407 |
| C | 1.899641 | 0.265781 | 0.420656 | H | 2.510575 | 2.770323 | 1.067348 |
| O | 1.328052 | 0.48766 | -0.846956 | H | 4.011765 | 2.554625 | 2.008187 |
| C | 3.40679 | 0.137035 | 0.507223 | H | 4.001088 | 3.605569 | 0.581184 |
| C | 3.719075 | -0.253285 | 1.964353 | H | 5.973077 | 1.241221 | 0.94484 |
| C | 4.014001 | 1.513308 | 0.087545 | H | 6.914629 | 2.729027 | -0.576754 |
| C | 3.601841 | 2.671857 | 0.993576 | H | 7.063229 | 0.349353 | -1.108457 |
| C | 5.537416 | 1.462969 | -0.052295 | H | 5.569211 | 0.64292 | -2.024316 |
| O | 5.955398 | 2.734048 | -0.493111 | H | 5.702036 | -1.767475 | -1.319462 |
| C | 5.964153 | 0.377484 | -1.030851 | H | 5.899757 | -1.264297 | 0.364439 |
| C | 5.432568 | -0.986583 | -0.596253 | H | 3.583848 | -2.648274 | -1.636976 |

| Conformer **2**_4 | | | | | | | |
|---|---|---|---|---|---|---|---|
| O | 0.848425 | -2.81153 | -1.206752 | C | 3.865183 | -1.091799 | -0.400157 |
| C | 1.555494 | -1.886371 | -0.8653 | C | 3.01646 | -1.924099 | -1.026971 |
| C | 0.980427 | -0.641792 | -0.234344 | H | -0.379337 | -2.669751 | 0.988256 |
| C | -0.482208 | -0.650265 | 0.21429 | H | -1.773728 | -1.703735 | 1.554429 |
| C | -0.692229 | -1.674469 | 1.329761 | H | -0.164701 | -0.427234 | 2.677899 |
| O | 0.070305 | -1.338454 | 2.457389 | H | -0.828076 | 1.264321 | 0.097716 |
| O | -0.796524 | 0.59319 | 0.797305 | H | -1.089125 | -0.266739 | -1.829512 |
| C | -1.388776 | -0.917355 | -0.994705 | H | -1.318225 | -1.966194 | -1.298041 |
| O | -2.752051 | -0.673176 | -0.665771 | H | -4.636612 | 2.802391 | -0.884848 |
| C | -3.209822 | 0.576338 | -0.809305 | H | -7.066861 | 3.084883 | -0.297732 |
| C | -4.653095 | 0.700936 | -0.451725 | H | -8.394306 | 1.10673 | 0.456687 |
| C | -5.243689 | 1.960896 | -0.552078 | H | -6.992025 | -2.088076 | 0.736125 |
| C | -6.590974 | 2.10613 | -0.224649 | H | -4.913565 | -1.383846 | 0.03885 |
| C | -7.339679 | 1.012239 | 0.196386 | H | 1.495124 | 0.899093 | 1.210817 |
| C | -6.742272 | -0.250303 | 0.294819 | H | 4.715719 | -0.641955 | 2.175811 |
| O | -7.51503 | -1.279294 | 0.708169 | H | 3.358045 | 0.39035 | 2.676542 |
| C | -5.394915 | -0.406728 | -0.03072 | H | 3.051572 | -1.293464 | 2.213492 |
| O | -2.531379 | 1.49508 | -1.195296 | H | 3.789141 | 1.595279 | -0.880665 |
| C | 1.922438 | 0.284972 | 0.413672 | H | 2.736467 | 2.718028 | 1.109796 |

| | | | | | | | |
|---|---|---|---|---|---|---|---|
| O | 1.395421 | 0.564666 | -0.861476 | H | 4.198923 | 2.364494 | 2.069765 |
| C | 3.411031 | 0.025729 | 0.526045 | H | 4.299975 | 3.429023 | 0.656644 |
| C | 3.660348 | -0.407157 | 1.983466 | H | 6.054799 | 0.896483 | 1.023634 |
| C | 4.143156 | 1.349046 | 0.136109 | H | 7.150396 | 2.316578 | -0.457692 |
| C | 3.816429 | 2.527875 | 1.050839 | H | 7.101385 | -0.060164 | -1.023493 |
| C | 5.658679 | 1.168024 | 0.022267 | H | 5.654927 | 0.372831 | -1.960156 |
| O | 6.193634 | 2.403354 | -0.392849 | H | 5.567188 | -2.048841 | -1.285338 |
| C | 6.007627 | 0.062015 | -0.963971 | H | 5.777146 | -1.584097 | 0.408113 |
| C | 5.352918 | -1.256004 | -0.556679 | H | 3.388166 | -2.744216 | -1.645151 |

| Conformer **2**_5 | | | | | | | |
|---|---|---|---|---|---|---|---|
| O | 0.861303 | -2.798055 | -1.242474 | C | 3.873071 | -1.089881 | -0.390263 |
| C | 1.565834 | -1.879009 | -0.880899 | C | 3.028514 | -1.916661 | -1.029861 |
| C | 0.985262 | -0.643636 | -0.236414 | H | -0.365378 | -2.688326 | 0.965233 |
| C | -0.477935 | -0.662081 | 0.210175 | H | -1.764669 | -1.735834 | 1.539986 |
| C | -0.683541 | -1.697858 | 1.315893 | H | -0.16963 | -0.464382 | 2.682009 |
| O | 0.077874 | -1.368414 | 2.446588 | H | -0.836852 | 1.250697 | 0.109144 |
| O | -0.796417 | 0.575351 | 0.804113 | H | -1.083874 | -0.2665 | -1.832167 |
| C | -1.383814 | -0.921801 | -1.001081 | H | -1.314085 | -1.968828 | -1.310553 |
| O | -2.74686 | -0.679296 | -0.670783 | H | -4.62492 | 2.79992 | -0.870615 |
| C | -3.204655 | 0.569139 | -0.805248 | H | -7.054084 | 3.087001 | -0.284772 |
| C | -4.649266 | 0.692516 | -0.448383 | H | -8.387123 | 1.130197 | 0.453077 |
| C | -5.235633 | 1.959073 | -0.543198 | H | -8.335187 | -1.116946 | 0.881143 |
| C | -6.578866 | 2.107573 | -0.217512 | H | -4.934885 | -1.400294 | 0.036594 |
| C | -7.331177 | 1.008858 | 0.197728 | H | 1.491256 | 0.881416 | 1.228974 |
| C | -6.73959 | -0.254632 | 0.289921 | H | 4.730139 | -0.616579 | 2.185483 |
| O | -7.421653 | -1.352211 | 0.685859 | H | 3.349833 | 0.384328 | 2.687919 |
| C | -5.390177 | -0.41376 | -0.035868 | H | 3.080117 | -1.303933 | 2.221009 |
| O | -2.526896 | 1.492532 | -1.183271 | H | 3.771593 | 1.583049 | -0.874992 |
| C | 1.922283 | 0.277163 | 0.426247 | H | 2.722612 | 2.737097 | 1.078744 |
| O | 1.398892 | 0.571734 | -0.848045 | H | 4.149065 | 2.35382 | 2.08151 |
| C | 3.411992 | 0.024273 | 0.536283 | H | 4.313287 | 3.426809 | 0.687836 |
| C | 3.669988 | -0.404443 | 1.993071 | H | 6.070106 | 0.941067 | 1.02279 |
| C | 4.138898 | 1.349906 | 0.143257 | H | 5.968634 | 2.63017 | -1.22172 |
| C | 3.800581 | 2.528959 | 1.052866 | H | 7.114115 | -0.040278 | -0.990585 |
| C | 5.661905 | 1.18224 | 0.025798 | H | 5.671585 | 0.349964 | -1.950562 |
| O | 6.287383 | 2.388728 | -0.343173 | H | 5.58355 | -2.049233 | -1.256489 |
| C | 6.020869 | 0.063362 | -0.942669 | H | 5.780516 | -1.573919 | 0.435345 |
| C | 5.362228 | -1.252638 | -0.534009 | H | 3.404633 | -2.733418 | -1.649857 |

| Conformer **2**_6 | | | | | | | |
|---|---|---|---|---|---|---|---|
| O | 0.857803 | -2.814772 | -1.210462 | C | 3.869632 | -1.089693 | -0.397002 |
| C | 1.562061 | -1.888487 | -0.866905 | C | 3.023728 | -1.923619 | -1.025272 |
| C | 0.983388 | -0.645158 | -0.236893 | H | -0.369888 | -2.673543 | 0.99096 |
| C | -0.479443 | -0.656829 | 0.210932 | H | -1.767634 | -1.711725 | 1.554197 |
| C | -0.686471 | -1.678384 | 1.329456 | H | -0.166988 | -0.426992 | 2.676868 |
| O | 0.075406 | -1.336144 | 2.456166 | H | -0.828296 | 1.256359 | 0.087096 |
| O | -0.797032 | 0.588171 | 0.789449 | H | -1.086866 | -0.284027 | -1.835087 |
| C | -1.385903 | -0.930456 | -0.996767 | H | -1.315754 | -1.98084 | -1.294631 |
| O | -2.748771 | -0.68512 | -0.667823 | H | -4.630057 | 2.789583 | -0.907996 |
| C | -3.20746 | 0.561455 | -0.816473 | H | -7.057511 | 3.08296 | -0.317652 |
| C | -4.651117 | 0.688432 | -0.456081 | H | -8.386185 | 1.135584 | 0.452598 |
| C | -5.238985 | 1.953002 | -0.566581 | H | -8.330699 | -1.104781 | 0.912182 |
| C | -6.581181 | 2.104974 | -0.238241 | H | -4.933053 | -1.397496 | 0.059339 |
| C | -7.331039 | 1.011589 | 0.195179 | H | 1.492419 | 0.897205 | 1.208205 |
| C | -6.73793 | -0.249906 | 0.303075 | H | 3.05019 | -1.290219 | 2.21555 |
| O | -7.417718 | -1.342358 | 0.717182 | H | 4.713888 | -0.637078 | 2.180314 |
| C | -5.389583 | -0.41251 | -0.025361 | H | 3.35402 | 0.394171 | 2.677568 |
| O | -2.532002 | 1.480303 | -1.20867 | H | 3.789108 | 1.596531 | -0.879781 |
| C | 1.922507 | 0.283668 | 0.41214 | H | 2.730008 | 2.718245 | 1.107798 |
| O | 1.397174 | 0.561901 | -0.864105 | H | 4.191642 | 2.369337 | 2.070645 |
| C | 3.411392 | 0.027573 | 0.52752 | H | 4.292593 | 3.432881 | 0.656723 |
| C | 3.658604 | -0.403609 | 1.985793 | H | 6.052185 | 0.903619 | 1.029835 |
| C | 4.141646 | 1.351938 | 0.137904 | H | 7.148459 | 2.323814 | -0.451725 |
| C | 3.810568 | 2.530891 | 1.050889 | H | 7.105166 | -0.052169 | -1.014427 |
| C | 5.657747 | 1.173819 | 0.027434 | H | 5.659669 | 0.377028 | -1.954404 |
| O | 6.191619 | 2.409897 | -0.387206 | H | 5.57539 | -2.044242 | -1.277715 |
| C | 6.011002 | 0.06778 | -0.957238 | H | 5.78099 | -1.577551 | 0.415757 |
| C | 5.358046 | -1.251196 | -0.550186 | H | 3.398111 | -2.743331 | -1.642393 |

| Conformer **2**_7 | | | | | | | |
|---|---|---|---|---|---|---|---|
| O | 1.066907 | -2.928581 | -1.142664 | C | 3.941702 | -0.951448 | -0.409637 |
| C | 1.698994 | -1.942942 | -0.824642 | C | 3.155693 | -1.861254 | -1.009945 |
| C | 1.031913 | -0.741803 | -0.20019 | H | -0.129987 | -2.854747 | 1.079698 |
| C | -0.416973 | -0.865196 | 0.275307 | H | -1.592174 | -2.001242 | 1.654811 |
| C | -0.52106 | -1.883794 | 1.410611 | H | -0.081076 | -0.572816 | 2.728991 |
| O | 0.228679 | -1.463919 | 2.518958 | H | -0.918941 | 1.012716 | 0.134828 |
| O | -0.824074 | 0.357897 | 0.844119 | H | -1.087792 | -0.571852 | -1.763789 |
| C | -1.319681 | -1.227676 | -0.911627 | H | -1.171059 | -2.273242 | -1.196812 |
| O | -2.691853 | -1.087356 | -0.561099 | H | -4.795303 | -1.95702 | 0.212096 |
| C | -3.251704 | 0.116655 | -0.718979 | H | -7.217197 | -1.825202 | 0.828893 |

| | | | | | | | |
|---|---|---|---|---|---|---|---|
| C | -4.695184 | 0.128668 | -0.335441 | H | -8.440365 | 0.320196 | 0.619421 |
| C | -5.345207 | -1.021498 | 0.126133 | H | -8.270615 | 2.521696 | 0.019636 |
| C | -6.691576 | -0.940724 | 0.466787 | H | -4.854738 | 2.225291 | -0.819647 |
| C | -7.382441 | 0.264793 | 0.349759 | H | 1.439303 | 0.85807 | 1.214494 |
| C | -6.726561 | 1.410087 | -0.113379 | H | 3.192188 | -1.173622 | 2.222757 |
| O | -7.348225 | 2.602487 | -0.246225 | H | 4.795163 | -0.386318 | 2.142764 |
| C | -5.375318 | 1.33887 | -0.457356 | H | 3.3645 | 0.538153 | 2.651159 |
| O | -2.658292 | 1.080178 | -1.134898 | H | 3.629532 | 1.708432 | -0.933411 |
| C | 1.90348 | 0.270209 | 0.418164 | H | 2.517907 | 2.777095 | 1.052798 |
| O | 1.333631 | 0.48566 | -0.85108 | H | 4.017975 | 2.563844 | 1.996123 |
| C | 3.410349 | 0.139419 | 0.506918 | H | 4.010062 | 3.607382 | 0.563666 |
| C | 3.720643 | -0.244067 | 1.966297 | H | 5.977917 | 1.241175 | 0.94139 |
| C | 4.02017 | 1.512595 | 0.08088 | H | 6.923646 | 2.719675 | -0.586996 |
| C | 3.609052 | 2.676438 | 0.980571 | H | 7.068517 | 0.337847 | -1.106776 |
| C | 5.543608 | 1.458969 | -0.05724 | H | 5.575657 | 0.629391 | -2.025242 |
| O | 5.964413 | 2.727144 | -0.503688 | H | 5.703894 | -1.777763 | -1.30871 |
| C | 5.969405 | 0.368111 | -1.030199 | H | 5.901414 | -1.266803 | 0.372891 |
| C | 5.435266 | -0.992946 | -0.589428 | H | 3.584665 | -2.65722 | -1.622608 |

| Conformer 3_1 | | | | | | | |
|---|---|---|---|---|---|---|---|
| C | -2.673459 | 3.308165 | 1.029953 | O | 5.533303 | 3.18304 | 0.388325 |
| C | -3.049825 | 3.031357 | -0.419329 | O | -2.557989 | -3.577603 | -0.373049 |
| C | -2.30635 | 1.801754 | -0.963801 | H | -3.234155 | 4.18189 | 1.391275 |
| C | -2.511182 | 0.527242 | -0.083731 | H | -1.599872 | 3.56504 | 1.067904 |
| C | -2.222549 | 0.874325 | 1.369202 | H | -4.136313 | 2.845223 | -0.479801 |
| C | -2.932838 | 2.08641 | 1.908831 | H | -1.225875 | 2.030218 | -0.882533 |
| C | -1.569362 | -0.536089 | -0.611366 | H | -2.624281 | 2.269932 | 2.946517 |
| C | -0.714682 | -1.365822 | 0.252905 | H | -4.017814 | 1.885516 | 1.927661 |
| C | -0.74905 | -1.096133 | 1.736676 | H | -1.860561 | -1.000204 | -1.557637 |
| C | -1.412903 | 0.147706 | 2.158373 | H | -1.272557 | 0.409599 | 3.209514 |
| O | -0.270468 | -1.872264 | 2.536382 | H | 1.565726 | -3.82602 | -0.391646 |
| O | -0.189097 | -0.285144 | -0.505888 | H | 1.020615 | -3.552111 | 1.308741 |
| C | -0.298699 | -2.759315 | -0.222727 | H | -1.464458 | -3.728271 | 1.334324 |
| C | 1.096928 | -3.117654 | 0.307424 | H | -0.917683 | -4.800515 | 0.013667 |
| C | -1.323204 | -3.797295 | 0.247848 | H | 0.462272 | -2.407334 | -1.975551 |
| O | -0.349201 | -2.818443 | -1.628609 | H | -3.705873 | 1.361358 | -2.579638 |
| C | -2.638643 | 1.588405 | -2.43901 | H | -2.04607 | 0.778345 | -2.884496 |
| C | -3.931596 | -0.059331 | -0.181873 | H | -2.429535 | 2.510306 | -2.994612 |
| O | -2.827996 | 4.166359 | -1.223166 | H | -3.993244 | -0.976997 | 0.420574 |
| O | 1.933129 | -1.975313 | 0.47813 | H | -4.700118 | 0.644807 | 0.164382 |
| C | 2.411379 | -1.376193 | -0.613959 | H | -4.163515 | -0.332169 | -1.2207 |

| | | | | | | | |
|---|---|---|---|---|---|---|---|
| C | 3.235874 | -0.187068 | -0.299367 | H | -1.883778 | 4.365334 | -1.197963 |
| O | 2.189579 | -1.771956 | -1.736612 | H | 3.656986 | 0.156413 | -2.375951 |
| C | 3.821886 | 0.515972 | -1.359456 | H | 5.058404 | 2.204438 | -1.925163 |
| C | 4.590928 | 1.642491 | -1.116787 | H | 4.33869 | 1.735704 | 2.288413 |
| C | 4.779202 | 2.082523 | 0.200607 | H | 2.958696 | -0.290304 | 1.834695 |
| C | 4.191319 | 1.386051 | 1.263699 | H | 5.595273 | 3.381625 | 1.329422 |
| C | 3.423236 | 0.255492 | 1.013308 | H | -2.369501 | -3.519188 | -1.320033 |

| Conformer 3_2 | | | | | | | |
|---|---|---|---|---|---|---|---|
| C | -2.727142 | 3.277302 | 1.024645 | O | 5.546093 | 3.222035 | 0.380061 |
| C | -3.123322 | 2.9788 | -0.414738 | O | -2.514562 | -3.596027 | -0.373133 |
| C | -2.350245 | 1.77729 | -0.963555 | H | -3.302461 | 4.13741 | 1.404416 |
| C | -2.520336 | 0.501078 | -0.079326 | H | -1.663019 | 3.562066 | 1.030551 |
| C | -2.230505 | 0.854498 | 1.371966 | H | -4.207771 | 2.741786 | -0.448119 |
| C | -2.954433 | 2.057386 | 1.914401 | H | -1.281075 | 2.045317 | -0.893487 |
| C | -1.558225 | -0.542596 | -0.609735 | H | -2.635029 | 2.252275 | 2.946695 |
| C | -0.695794 | -1.364857 | 0.254252 | H | -4.035224 | 1.835781 | 1.949563 |
| C | -0.73464 | -1.09851 | 1.738238 | H | -1.841309 | -1.008811 | -1.557339 |
| C | -1.410916 | 0.138411 | 2.160368 | H | -1.270483 | 0.403442 | 3.210706 |
| O | -0.249715 | -1.871021 | 2.537803 | H | 1.610463 | -3.8009 | -0.392654 |
| O | -0.182002 | -0.275939 | -0.500476 | H | 1.063451 | -3.531934 | 1.307958 |
| C | -0.264142 | -2.752938 | -0.223038 | H | -1.418081 | -3.73724 | 1.333334 |
| C | 1.13526 | -3.096869 | 0.306537 | H | -0.860509 | -4.801152 | 0.010301 |
| C | -1.276825 | -3.802854 | 0.246675 | H | 0.491719 | -2.390269 | -1.975758 |
| O | -0.314514 | -2.811894 | -1.628916 | H | -3.75904 | 1.341344 | -2.575276 |
| C | -2.688606 | 1.556456 | -2.436565 | H | -2.107479 | 0.734006 | -2.874267 |
| C | -3.925291 | -0.124139 | -0.167629 | H | -2.459688 | 2.471061 | -2.995529 |
| O | -2.839907 | 4.074272 | -1.254371 | H | -3.950857 | -1.056354 | 0.414904 |
| O | 1.960682 | -1.946872 | 0.476591 | H | -4.70823 | 0.549339 | 0.206114 |
| C | 2.434402 | -1.345035 | -0.61633 | H | -4.165445 | -0.380291 | -1.208982 |
| C | 3.255444 | -0.153427 | -0.303303 | H | -3.334195 | 4.83763 | -0.938168 |
| O | 2.212197 | -1.742127 | -1.738476 | H | 3.664979 | 0.195834 | -2.38127 |
| C | 3.834357 | 0.553361 | -1.364832 | H | 5.061888 | 2.247266 | -1.933333 |
| C | 4.601224 | 1.681579 | -1.123703 | H | 4.363872 | 1.768444 | 2.282648 |
| C | 4.794094 | 2.119816 | 0.193536 | H | 2.988013 | -0.261016 | 1.831764 |
| C | 4.213266 | 1.419715 | 1.258089 | H | 5.605475 | 3.422757 | 1.320831 |
| C | 3.447396 | 0.287402 | 1.00927 | H | -2.326271 | -3.528432 | -1.31961 |

| Conformer 3_3 | | | | | | | |
|---|---|---|---|---|---|---|---|
| C | 6.058903 | 0.183854 | -0.967845 | O | -8.64961 | 0.773017 | 0.443007 |

| | | | | | | | |
|---|---|---|---|---|---|---|---|
| C | 5.656399 | 1.335991 | -0.057138 | O | 0.192906 | -1.334753 | 2.530259 |
| C | 4.128089 | 1.44396 | 0.061893 | H | 7.155366 | 0.124803 | -1.017758 |
| C | 3.461498 | 0.111 | 0.530035 | H | 5.692892 | 0.40158 | -1.986968 |
| C | 3.965332 | -1.029238 | -0.34062 | H | 6.07927 | 1.165606 | 0.948289 |
| C | 5.459427 | -1.135787 | -0.486287 | H | 3.746228 | 1.606135 | -0.96472 |
| C | 1.961689 | 0.294017 | 0.418077 | H | 5.711127 | -1.958988 | -1.167862 |
| C | 1.061309 | -0.698645 | -0.189746 | H | 5.895798 | -1.387812 | 0.495487 |
| C | 1.691234 | -1.939576 | -0.774588 | H | 1.508762 | 0.919859 | 1.191676 |
| C | 3.153653 | -1.922746 | -0.931141 | H | 3.561235 | -2.753724 | -1.511073 |
| O | 1.025678 | -2.905406 | -1.084824 | H | -1.180026 | -2.157568 | -1.193479 |
| O | 1.420262 | 0.499735 | -0.865696 | H | -1.028445 | -0.469277 | -1.784317 |
| C | -0.397983 | -0.754976 | 0.267273 | H | -0.197986 | -2.739198 | 1.112841 |
| C | -1.296964 | -1.103198 | -0.9263 | H | -1.634669 | -1.819828 | 1.649383 |
| C | -0.556486 | -1.747822 | 1.419071 | H | -0.86637 | 1.128969 | 0.083717 |
| O | -0.762592 | 0.493405 | 0.809304 | H | 4.10102 | 2.548749 | 1.944365 |
| C | 3.743013 | 2.654457 | 0.909505 | H | 2.657357 | 2.818578 | 0.929205 |
| C | 3.745776 | -0.228715 | 2.005164 | H | 4.216851 | 3.552179 | 0.494965 |
| O | 6.227607 | 2.546897 | -0.49343 | H | 3.196812 | -1.139648 | 2.284258 |
| O | -2.668646 | -0.910256 | -0.601484 | H | 4.815135 | -0.384485 | 2.200496 |
| C | -3.184348 | 0.312418 | -0.786205 | H | 3.39519 | 0.580971 | 2.660113 |
| C | -4.627418 | 0.379623 | -0.447328 | H | 5.892945 | 2.728752 | -1.380389 |
| O | -2.541026 | 1.249946 | -1.193548 | H | -4.709063 | 2.46639 | -0.943494 |
| C | -5.283765 | 1.609206 | -0.590223 | H | -7.160641 | 2.675609 | -0.39293 |
| C | -6.630688 | 1.728934 | -0.289223 | H | -7.252071 | -1.492954 | 0.657948 |
| C | -7.342627 | 0.609125 | 0.16218 | H | -4.830291 | -1.69114 | 0.110843 |
| C | -6.692634 | -0.622709 | 0.306326 | H | -9.02845 | -0.060843 | 0.743231 |
| C | -5.34135 | -0.734876 | 0.001817 | H | -0.086962 | -0.429166 | 2.71928 |

| Conformer 3_4 | | | | | | | |
|---|---|---|---|---|---|---|---|
| C | 6.057584 | 0.191945 | -0.970176 | O | -8.653281 | 0.648201 | 0.473502 |
| C | 5.652948 | 1.343946 | -0.060245 | O | 0.199137 | -1.334538 | 2.533039 |
| C | 4.124463 | 1.448602 | 0.05945 | H | 7.154155 | 0.135212 | -1.02046 |
| C | 3.461086 | 0.114586 | 0.529065 | H | 5.690729 | 0.408148 | -1.98933 |
| C | 3.966822 | -1.025109 | -0.341209 | H | 6.076712 | 1.17544 | 0.945114 |
| C | 5.461078 | -1.128639 | -0.487515 | H | 3.741734 | 1.609049 | -0.967115 |
| C | 1.960805 | 0.294264 | 0.41812 | H | 5.714166 | -1.951701 | -1.16874 |
| C | 1.062168 | -0.700526 | -0.188639 | H | 5.898454 | -1.379217 | 0.494176 |
| C | 1.694314 | -1.940237 | -0.773651 | H | 1.507063 | 0.919312 | 1.191877 |
| C | 3.156677 | -1.920524 | -0.930905 | H | 3.565619 | -2.750962 | -1.510654 |
| O | 1.030553 | -2.907358 | -1.083481 | H | -1.178644 | -2.167821 | -1.185988 |
| O | 1.417931 | 0.498444 | -0.865365 | H | -1.029092 | -0.481201 | -1.78211 |

| | | | | | | | |
|---|---|---|---|---|---|---|---|
| C | -0.39649 | -0.76001 | 0.269981 | H | -0.19027 | -2.742253 | 1.118473 |
| C | -1.296085 | -1.11276 | -0.921879 | H | -1.628528 | -1.82602 | 1.655704 |
| C | -0.550948 | -1.751332 | 1.423643 | H | -0.868126 | 1.122389 | 0.082908 |
| O | -0.763664 | 0.488595 | 0.809916 | H | 4.095557 | 2.554829 | 1.941043 |
| C | 3.737101 | 2.65898 | 0.906181 | H | 2.651089 | 2.82075 | 0.926032 |
| C | 3.747166 | -0.223481 | 2.004218 | H | 4.208986 | 3.557341 | 0.490781 |
| O | 6.221205 | 2.555787 | -0.497952 | H | 3.200324 | -1.135364 | 2.284351 |
| O | -2.667689 | -0.920094 | -0.596323 | H | 4.816996 | -0.37683 | 2.198885 |
| C | -3.184848 | 0.300842 | -0.783914 | H | 3.395279 | 0.585867 | 2.658891 |
| C | -4.627919 | 0.36695 | -0.444367 | H | 5.886337 | 2.73549 | -1.385267 |
| O | -2.543216 | 1.238553 | -1.194364 | H | -4.831671 | -1.703319 | 0.120536 |
| C | -5.344784 | -0.748334 | 0.009329 | H | -7.271047 | -1.491956 | 0.6682 |
| C | -6.693066 | -0.638803 | 0.313445 | H | -7.142403 | 2.67024 | -0.403524 |
| C | -7.343339 | 0.593525 | 0.165239 | H | -4.708518 | 2.450747 | -0.946658 |
| C | -6.631642 | 1.711078 | -0.289011 | H | -8.987361 | 1.541015 | 0.331139 |
| C | -5.281506 | 1.593461 | -0.59099 | H | -0.084845 | -0.43046 | 2.722945 |

| Conformer 3_5 | | | | | | | |
|---|---|---|---|---|---|---|---|
| C | -3.6856563 | 3.12944699 | -3.057654 | O | 4.99516989 | -5.2533028 | 5.77387385 |
| C | -2.6464934 | 4.03089438 | -2.3962828 | O | 1.95465975 | -0.9474846 | -1.8312765 |
| C | -1.7850212 | 3.26176965 | -1.3681527 | H | -4.2510616 | 3.70146419 | -3.8036753 |
| C | -1.1214875 | 1.97213608 | -1.9780573 | H | -4.4250448 | 2.80827593 | -2.3115856 |
| C | -2.1939873 | 1.1609315 | -2.7213721 | H | -2.0051775 | 4.48384606 | -3.1612446 |
| C | -3.0420129 | 1.91945332 | -3.7146972 | H | -2.4582222 | 2.94702288 | -0.5562146 |
| C | -0.437107 | 1.18855446 | -0.8638235 | H | -3.8319367 | 1.28379336 | -4.133249 |
| C | -0.6810311 | -0.3021603 | -0.671354 | H | -2.4241175 | 2.2355426 | -4.5629935 |
| C | -1.6768958 | -0.9757863 | -1.5484142 | H | 0.51590718 | 1.61008717 | -0.5700242 |
| C | -2.4181182 | -0.1494557 | -2.5236225 | H | -3.1826055 | -0.6902514 | -3.0726157 |
| O | -1.9054113 | -2.1768144 | -1.4613067 | H | -0.8178154 | -2.8976608 | 0.54135513 |
| O | -1.283066 | 0.69899753 | 0.18550701 | H | -0.9208877 | -1.6085175 | 1.72453238 |
| C | 0.39873219 | -1.1379444 | 0.03015852 | H | 0.66652794 | -2.5423163 | -1.6580273 |
| C | -0.230631 | -2.1258372 | 1.04600107 | H | 2.02314775 | -2.5011353 | -0.5166206 |
| C | 1.25688033 | -1.8916806 | -1.007651 | H | 0.92228779 | -0.031652 | 1.57801736 |
| O | 1.32837024 | -0.2526194 | 0.71172947 | H | -0.1113537 | 4.67729318 | -1.4568809 |
| C | -0.7767041 | 4.22509658 | -0.7148326 | H | -0.1632295 | 3.7209904 | 0.03716019 |
| C | -0.0163345 | 2.32530869 | -3.0083861 | H | -1.2952202 | 5.04113399 | -0.1998156 |
| O | -3.330566 | 5.09818771 | -1.7390545 | H | 0.38861033 | 1.41644455 | -3.4714299 |
| O | 0.76907336 | -2.8310756 | 1.81301105 | H | -0.3772958 | 2.97223783 | -3.8134772 |
| C | 1.21383595 | -2.1911555 | 2.92332462 | H | 0.83062264 | 2.83532101 | -2.5357321 |
| C | 2.20775393 | -3.0212467 | 3.65471834 | H | -3.8499543 | 5.57677221 | -2.4076114 |
| O | 0.86641462 | -1.0746742 | 3.27971829 | H | 2.1527368 | -4.7400553 | 2.32539517 |

| | | | | | | | |
|---|---|---|---|---|---|---|---|
| C | 2.58177199 | -4.3012706 | 3.22253411 | H | 3.81368917 | -6.0301308 | 3.61632145 |
| C | 3.52127663 | -5.0374515 | 3.94851592 | H | 4.15827616 | -2.8002 | 6.44128425 |
| C | 4.08350867 | -4.4972135 | 5.09989185 | H | 2.50268763 | -1.4976732 | 5.16570426 |
| C | 3.72384343 | -3.2287905 | 5.54340407 | H | 5.30352427 | -4.7559879 | 6.54930997 |
| C | 2.78417237 | -2.490476 | 4.81882777 | H | 2.38096494 | -0.3389822 | -1.1931607 |

| Conformer 4_1 | | | | | | | |
|---|---|---|---|---|---|---|---|
| C | 1.937348 | -0.553228 | -2.071236 | H | 2.816218 | -0.946315 | -2.599989 |
| C | 2.374039 | 0.068302 | -0.754853 | H | 1.480582 | 0.233313 | -2.692778 |
| C | 1.195029 | 0.617401 | 0.049127 | H | 2.927819 | -0.680427 | -0.164011 |
| C | 0.108134 | -0.476119 | 0.306843 | H | 0.70396 | 1.376042 | -0.584624 |
| C | -0.240066 | -1.158185 | -1.009309 | H | 0.571302 | -2.105808 | -2.752946 |
| C | 0.926069 | -1.668591 | -1.810726 | H | 1.423126 | -2.477341 | -1.248255 |
| C | -1.090082 | 0.229525 | 0.916903 | H | -0.9933 | 0.522403 | 1.96784 |
| C | -2.474895 | 0.015061 | 0.456586 | H | -1.698177 | -1.871579 | -2.378148 |
| C | -2.694535 | -0.912549 | -0.707326 | H | -5.310032 | 1.5262 | 1.559427 |
| C | -1.499515 | -1.336062 | -1.447354 | H | -4.604399 | 1.495793 | -0.07436 |
| O | -3.809704 | -1.291264 | -1.008898 | H | -3.04871 | -1.418718 | 2.739561 |
| O | 3.254098 | 1.166924 | -1.005536 | H | -4.777577 | -0.769296 | 2.975473 |
| O | -1.739326 | 1.183017 | 0.116956 | H | -5.465913 | -0.67638 | -0.185371 |
| C | -3.636877 | 0.238655 | 1.38258 | H | 5.316575 | 2.751526 | -0.60107 |
| C | -4.893796 | 0.864217 | 0.786982 | H | 6.370922 | 1.859373 | -1.753698 |
| C | -3.774208 | -0.615327 | 2.567787 | H | 4.884789 | 2.70765 | -2.317826 |
| O | -3.260867 | 0.695637 | 2.665298 | H | 2.243699 | 0.595744 | 1.965019 |
| O | -5.870212 | -0.076652 | 0.45872 | H | 0.860353 | 1.721998 | 1.910941 |
| C | 4.540544 | 0.876159 | -1.269836 | H | 2.364143 | 2.118399 | 1.062613 |
| C | 5.340267 | 2.12777 | -1.50465 | H | -0.212706 | -2.316825 | 1.420273 |
| O | 4.967464 | -0.244639 | -1.303453 | H | 1.494144 | -2.045588 | 0.998923 |
| C | 1.686479 | 1.297243 | 1.325789 | H | 0.741117 | -1.099382 | 2.300263 |
| C | 0.566674 | -1.548078 | 1.31235 | | | | |

| Conformer 4_2 | | | | | | | |
|---|---|---|---|---|---|---|---|
| C | 1.926264 | -0.607352 | -2.140065 | H | 2.809607 | -0.988587 | -2.670175 |
| C | 2.358565 | 0.034547 | -0.832149 | H | 1.451388 | 0.164846 | -2.766051 |
| C | 1.174193 | 0.570232 | -0.027251 | H | 2.928697 | -0.698246 | -0.236942 |
| C | 0.109434 | -0.540341 | 0.248133 | H | 0.665661 | 1.312611 | -0.666446 |
| C | -0.234798 | -1.243614 | -1.059103 | H | 0.585241 | -2.190709 | -2.798937 |
| C | 0.937377 | -1.73838 | -1.862841 | H | 1.453516 | -2.532888 | -1.297011 |
| C | -1.097933 | 0.150776 | 0.858009 | H | -1.000339 | 0.455459 | 1.905557 |
| C | -2.480048 | -0.093437 | 0.403851 | H | -1.685593 | -2.008725 | -2.405345 |

| | | | | | | | |
|---|---|---|---|---|---|---|---|
| C | -2.695188 | -1.060453 | -0.731847 | H | -4.406814 | 1.815638 | 0.295372 |
| C | -1.492702 | -1.455852 | -1.483315 | H | -5.299556 | 0.297344 | -0.008791 |
| O | -3.798213 | -1.491281 | -0.983244 | H | -3.131639 | -1.613212 | 2.614946 |
| O | 3.217726 | 1.146603 | -1.097869 | H | -4.848891 | -0.920962 | 2.84808 |
| O | -1.766688 | 1.083462 | 0.049009 | H | -5.256285 | 1.584351 | 2.497915 |
| C | -3.637931 | 0.121657 | 1.3323 | H | 5.25699 | 2.770688 | -0.715418 |
| C | -4.812824 | 0.901251 | 0.767968 | H | 6.316347 | 1.889458 | -1.871469 |
| C | -3.834886 | -0.78756 | 2.462608 | H | 4.810045 | 2.707284 | -2.427744 |
| O | -3.287177 | 0.509539 | 2.647014 | H | 2.237944 | 0.589851 | 1.880474 |
| O | -5.74785 | 1.18905 | 1.76549 | H | 0.830737 | 1.685536 | 1.826902 |
| C | 4.508199 | 0.877231 | -1.363552 | H | 2.319038 | 2.106026 | 0.962956 |
| C | 5.283296 | 2.141237 | -1.61502 | H | -0.171656 | -2.372399 | 1.385366 |
| O | 4.956915 | -0.235333 | -1.385919 | H | 1.526828 | -2.077277 | 0.947809 |
| C | 1.66108 | 1.273075 | 1.238824 | H | 0.768309 | -1.129427 | 2.24478 |
| C | 0.593613 | -1.591721 | 1.263143 | | | | |

| Conformer 4_3 | | | | | | | |
|---|---|---|---|---|---|---|---|
| C | 1.776101 | 0.705461 | -2.146369 | H | 2.566486 | 0.663189 | -2.90811 |
| C | 2.288912 | 0.098805 | -0.851138 | H | 1.526981 | 1.762516 | -1.961422 |
| C | 1.23238 | 0.106497 | 0.254026 | H | 2.637915 | -0.929673 | -1.043223 |
| C | -0.081802 | -0.613628 | -0.191075 | H | 0.949406 | 1.161346 | 0.413684 |
| C | -0.522112 | -0.073286 | -1.547077 | H | 0.12929 | 0.392819 | -3.53534 |
| C | 0.535713 | -0.051101 | -2.617368 | H | 0.822762 | -1.088178 | -2.862717 |
| C | -1.114234 | -0.342536 | 0.88987 | H | -0.987617 | -0.893035 | 1.828127 |
| C | -2.49411 | 0.094065 | 0.593712 | H | -2.056067 | 0.652398 | -2.82096 |
| C | -2.886547 | 0.255854 | -0.850669 | H | -5.310928 | 0.927087 | 0.881161 |
| C | -1.776622 | 0.326526 | -1.816657 | H | -5.158126 | 0.787332 | 2.663247 |
| O | -4.049189 | 0.279692 | -1.184446 | H | -3.43916 | -2.363746 | 1.444548 |
| O | 3.401795 | 0.855877 | -0.367097 | H | -4.96691 | -1.745163 | 2.300196 |
| O | -1.500393 | 0.996866 | 1.058031 | H | -3.185561 | 2.189719 | 2.216147 |
| C | -3.601421 | -0.15897 | 1.577236 | H | 6.62201 | 1.286142 | -0.811215 |
| C | -4.605448 | 0.965275 | 1.721406 | H | 5.385662 | 2.537744 | -0.423202 |
| C | -3.95485 | -1.533604 | 1.940188 | H | 5.741906 | 1.267465 | 0.757958 |
| O | -3.156998 | -0.740576 | 2.790991 | H | 2.185256 | -1.471351 | 1.425799 |
| O | -3.989935 | 2.221998 | 1.683593 | H | 1.084901 | -0.434458 | 2.372774 |
| C | 4.600238 | 0.614305 | -0.926913 | H | 2.666559 | 0.179088 | 1.862919 |
| C | 5.666364 | 1.481856 | -0.316444 | H | -0.852894 | -2.592871 | -0.656475 |
| O | 4.769497 | -0.195597 | -1.795894 | H | 0.886455 | -2.426129 | -0.987946 |
| C | 1.817019 | -0.442216 | 1.554619 | H | 0.314574 | -2.573295 | 0.687466 |
| C | 0.082742 | -2.140508 | -0.295938 | | | | |

| Conformer 4_4 | | | | | | | |
|---|---|---|---|---|---|---|---|
| C | 1.655531 | -0.106838 | -2.346121 | H | 2.412468 | -0.422087 | -3.077074 |
| C | 2.236597 | -0.198414 | -0.944931 | H | 1.395063 | 0.944594 | -2.546918 |
| C | 1.228545 | 0.206242 | 0.131262 | H | 2.598686 | -1.224776 | -0.7667 |
| C | -0.08612 | -0.63396 | 0.040061 | H | 0.930302 | 1.245748 | -0.089752 |
| C | -0.598117 | -0.618613 | -1.395888 | H | -0.044772 | -0.904712 | -3.447017 |
| C | 0.410004 | -0.985008 | -2.451234 | H | 0.704772 | -2.039983 | -2.317485 |
| C | -1.077893 | -0.006785 | 1.003485 | H | -0.907171 | -0.197939 | 2.068043 |
| C | -2.47692 | 0.28046 | 0.641275 | H | -2.193595 | -0.384375 | -2.77527 |
| C | -2.931422 | -0.027946 | -0.762942 | H | -4.759287 | 1.810552 | 0.72924 |
| C | -1.868282 | -0.334583 | -1.733821 | H | -5.15463 | 1.535548 | 2.447636 |
| O | -4.107702 | -0.035895 | -1.046375 | H | -3.7301 | -1.855257 | 1.769941 |
| O | 3.352402 | 0.687321 | -0.822778 | H | -5.126668 | -0.929924 | 2.568257 |
| O | -1.485898 | 1.308926 | 0.710806 | H | -2.694806 | 2.6832 | 1.695907 |
| C | -3.544228 | 0.35099 | 1.696282 | H | 6.541721 | 0.936351 | -1.544324 |
| C | -4.326175 | 1.645295 | 1.733066 | H | 5.299479 | 2.241191 | -1.566468 |
| C | -4.100436 | -0.912509 | 2.188786 | H | 5.73457 | 1.476029 | -0.029926 |
| O | -3.173407 | -0.175184 | 2.952727 | H | 2.260821 | -0.832766 | 1.75142 |
| O | -3.53704 | 2.717752 | 2.166339 | H | 1.180776 | 0.474221 | 2.305945 |
| C | 4.531006 | 0.262242 | -1.312373 | H | 2.726849 | 0.865171 | 1.534673 |
| C | 5.605763 | 1.293363 | -1.105164 | H | -0.829367 | -2.654255 | 0.353149 |
| O | 4.677458 | -0.806691 | -1.837167 | H | 0.889137 | -2.605055 | -0.103016 |
| C | 1.87792 | 0.171197 | 1.513666 | H | 0.394144 | -2.146458 | 1.54057 |
| C | 0.110021 | -2.09638 | 0.48021 | | | | |

| Conformer 4_5 | | | | | | | |
|---|---|---|---|---|---|---|---|
| C | 1.887005 | -0.858749 | -2.194085 | H | 2.702727 | -1.312311 | -2.775791 |
| C | 2.45131 | -0.227643 | -0.929752 | H | 1.414518 | -0.081834 | -2.810014 |
| C | 1.358058 | 0.428671 | -0.074386 | H | 2.935464 | -1.015307 | -0.330304 |
| C | 0.265892 | -0.616386 | 0.330059 | H | 0.83659 | 1.162498 | -0.707213 |
| C | -0.216816 | -1.333838 | -0.924095 | H | 0.402549 | -2.370878 | -2.69442 |
| C | 0.856399 | -1.91905 | -1.803046 | H | 1.363521 | -2.730991 | -1.253294 |
| C | -0.851812 | 0.154442 | 1.010505 | H | -0.655897 | 0.479243 | 2.038073 |
| C | -2.277717 | -0.028747 | 0.679653 | H | -1.810359 | -2.039186 | -2.137245 |
| C | -2.626779 | -0.988074 | -0.425179 | H | -4.956791 | 1.614757 | 1.970831 |
| C | -1.514287 | -1.481033 | -1.24656 | H | -4.394843 | 1.513759 | 0.285017 |
| O | -3.775422 | -1.33693 | -0.617321 | H | -2.699672 | -1.369605 | 3.050369 |
| O | 3.544198 | 0.656963 | -1.203039 | H | -4.379006 | -0.651127 | 3.410724 |
| O | -1.53824 | 1.100243 | 0.23469 | H | -5.331047 | -0.630674 | 0.315872 |
| C | -3.346966 | 0.265697 | 1.693474 | H | 5.408366 | 1.926733 | -2.462466 |
| C | -4.629423 | 0.917252 | 1.186958 | H | 4.457139 | 3.446795 | -2.652061 |

| | | | | | | | |
|---|---|---|---|---|---|---|---|
| C | -3.409971 | -0.546022 | 2.914092 | H | 4.966594 | 2.865933 | -1.027988 |
| O | -2.846575 | 0.747778 | 2.923538 | H | 2.590438 | 0.48704 | 1.73258 |
| O | -5.659812 | 0.001219 | 0.972187 | H | 1.204783 | 1.613225 | 1.758522 |
| C | 3.345722 | 1.78566 | -1.904319 | H | 2.621229 | 1.972855 | 0.76903 |
| C | 4.627654 | 2.564473 | -2.028504 | H | -0.040506 | -2.370638 | 1.576622 |
| O | 2.285532 | 2.120416 | -2.355753 | H | 1.622151 | -2.239345 | 0.965357 |
| C | 1.971987 | 1.159006 | 1.118128 | H | 1.074453 | -1.174322 | 2.27834 |
| C | 0.767659 | -1.661969 | 1.342213 | | | | |

| Conformer 4_6 | | | | | | | |
|---|---|---|---|---|---|---|---|
| C | 1.655469 | -0.362157 | -2.352597 | H | 2.463114 | -0.754686 | -2.985216 |
| C | 2.221313 | 0.044109 | -1.003271 | H | 1.235102 | 0.532182 | -2.839558 |
| C | 1.147136 | 0.596102 | -0.065529 | H | 2.723256 | -0.823332 | -0.542373 |
| C | -0.028875 | -0.413531 | 0.134229 | H | 0.703737 | 1.470048 | -0.574552 |
| C | -0.504034 | -0.930236 | -1.219449 | H | 0.122333 | -1.707225 | -3.115134 |
| C | 0.569243 | -1.416156 | -2.155736 | H | 1.02956 | -2.324394 | -1.729426 |
| C | -1.132674 | 0.328669 | 0.86513 | H | -0.915985 | 0.57218 | 1.913111 |
| C | -2.558619 | 0.185362 | 0.524787 | H | -2.096916 | -1.462955 | -2.516755 |
| C | -2.907791 | -0.694492 | -0.65151 | H | -3.039675 | -0.848836 | 3.257141 |
| C | -1.80026 | -1.023121 | -1.562167 | H | -4.798014 | -0.706307 | 2.989223 |
| O | -4.029064 | -1.124872 | -0.808027 | H | -3.36782 | 2.548411 | 1.665556 |
| O | 3.195693 | 1.077154 | -1.174129 | H | -4.34113 | 1.813211 | 3.07437 |
| O | -1.810887 | 1.337956 | 0.168277 | H | -4.323518 | -2.046764 | 1.053925 |
| C | -3.615057 | 0.352879 | 1.578479 | H | 5.425618 | 2.399453 | -0.718413 |
| C | -3.824451 | -0.845322 | 2.484115 | H | 6.323896 | 1.559665 | -2.031288 |
| C | -3.934388 | 1.701606 | 2.062861 | H | 4.904213 | 2.606621 | -2.398129 |
| O | -4.768642 | 1.036735 | 1.14241 | H | 2.316816 | 0.244637 | 1.744396 |
| O | -3.728001 | -2.070867 | 1.816571 | H | 1.040814 | 1.473982 | 1.943136 |
| C | 4.430645 | 0.701143 | -1.551444 | H | 2.509508 | 1.857532 | 1.031735 |
| C | 5.339706 | 1.891456 | -1.688287 | H | -0.528314 | -2.277387 | 1.126144 |
| O | 4.740581 | -0.441901 | -1.742984 | H | 1.169916 | -2.208602 | 0.580864 |
| C | 1.78148 | 1.065175 | 1.243357 | H | 0.65733 | -1.291509 | 2.014 |
| C | 0.347636 | -1.621615 | 1.01216 | | | | |

## Spectroscopic data

Figure S5. $^{1}H$ NMR spectrum of **1** in $CD_3OD$

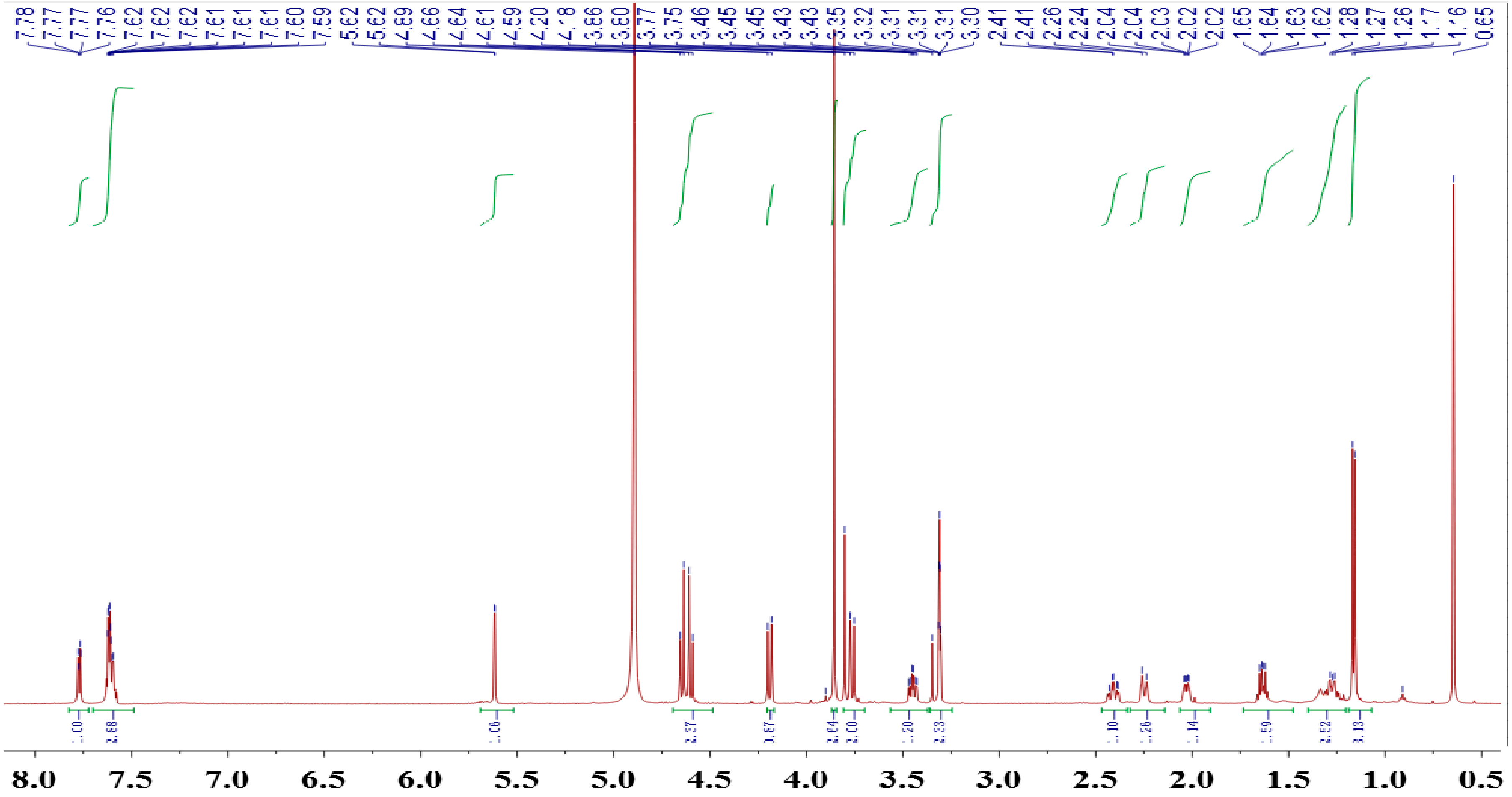

Figure S6 $^{13}$C NMR spectrum of **1** in $CD_3OD$

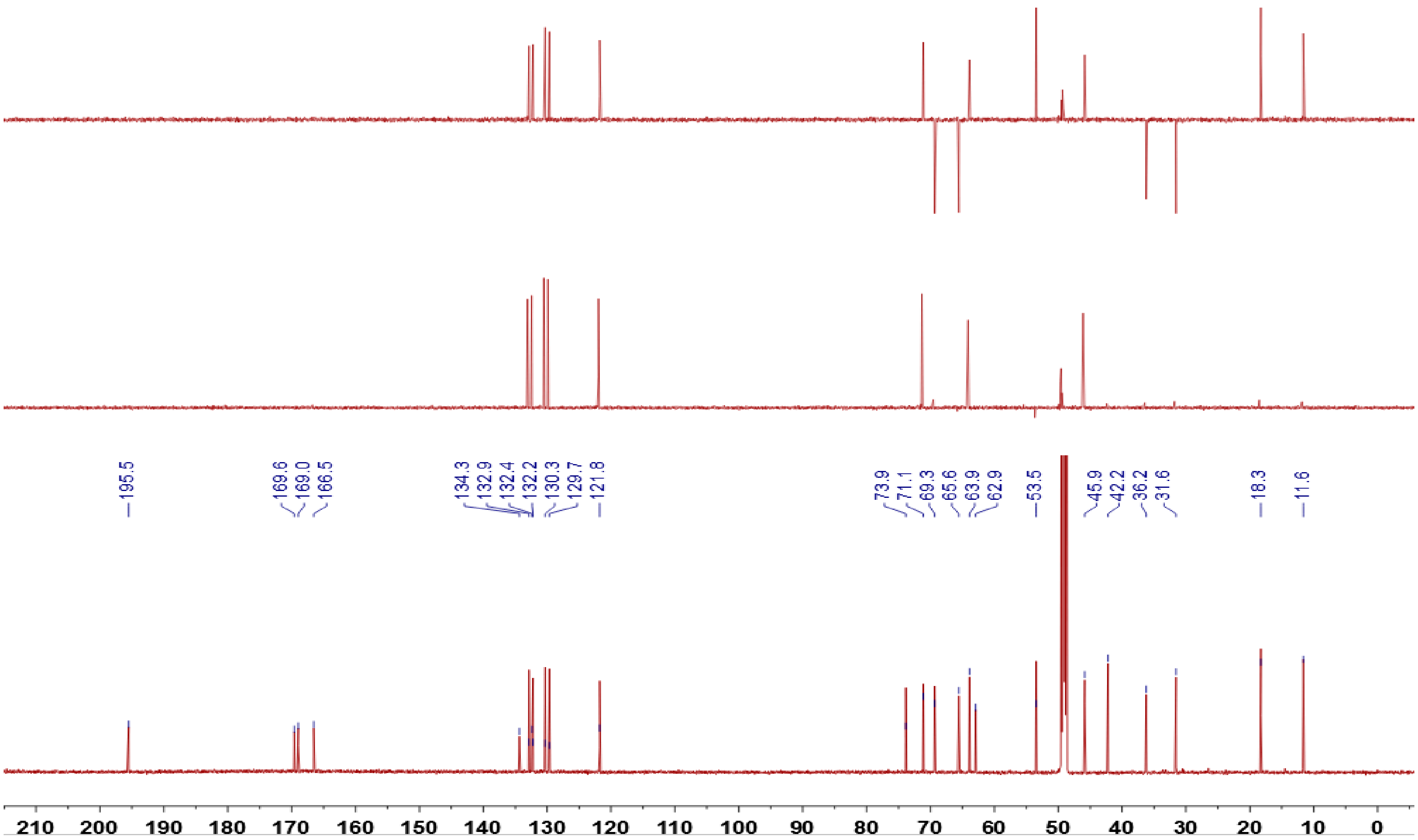

Figure S7 HSQC spectrum of **1** in $CD_3OD$

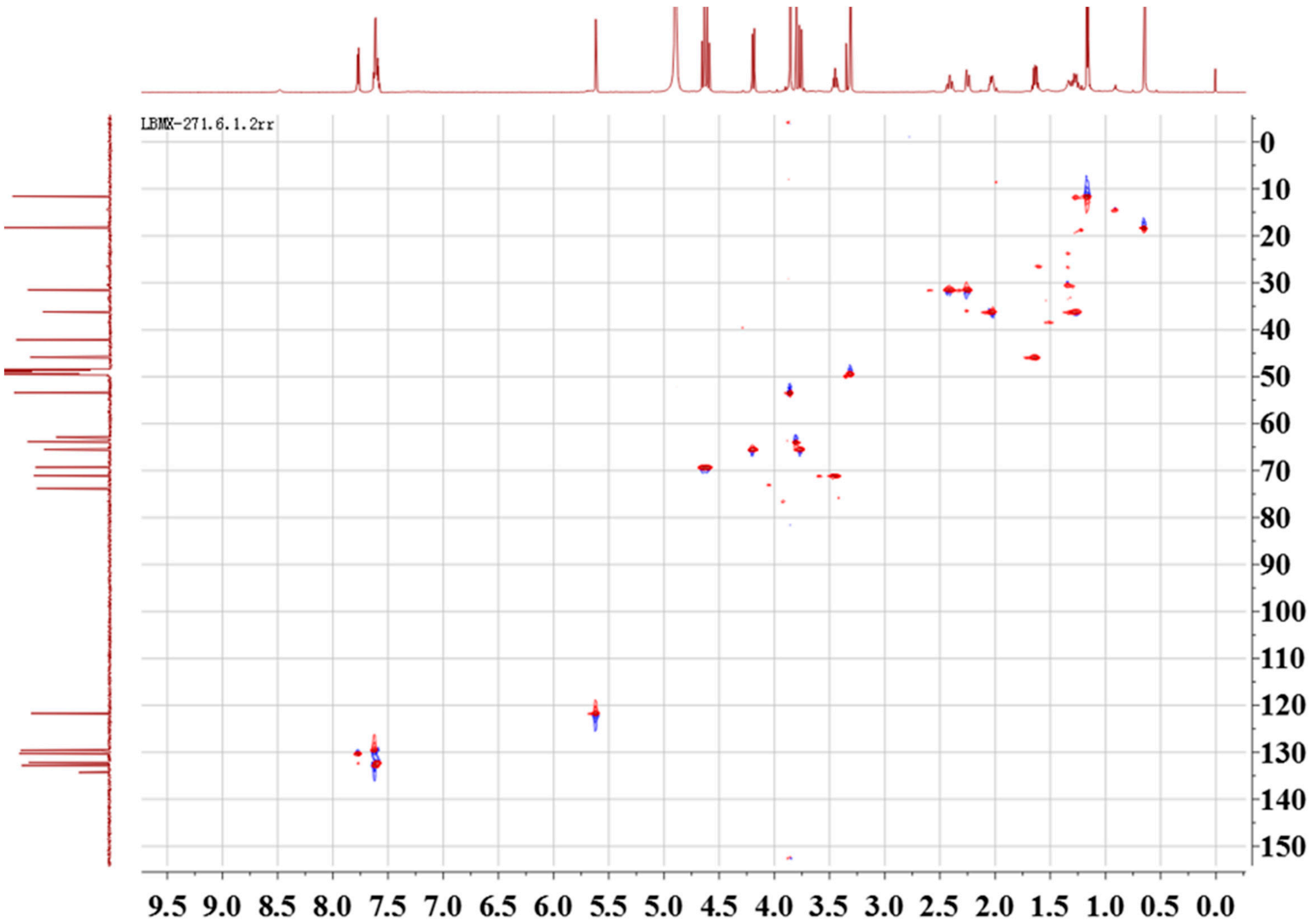

Figure S8 HMBC spectrum of **1** in $CD_3OD$

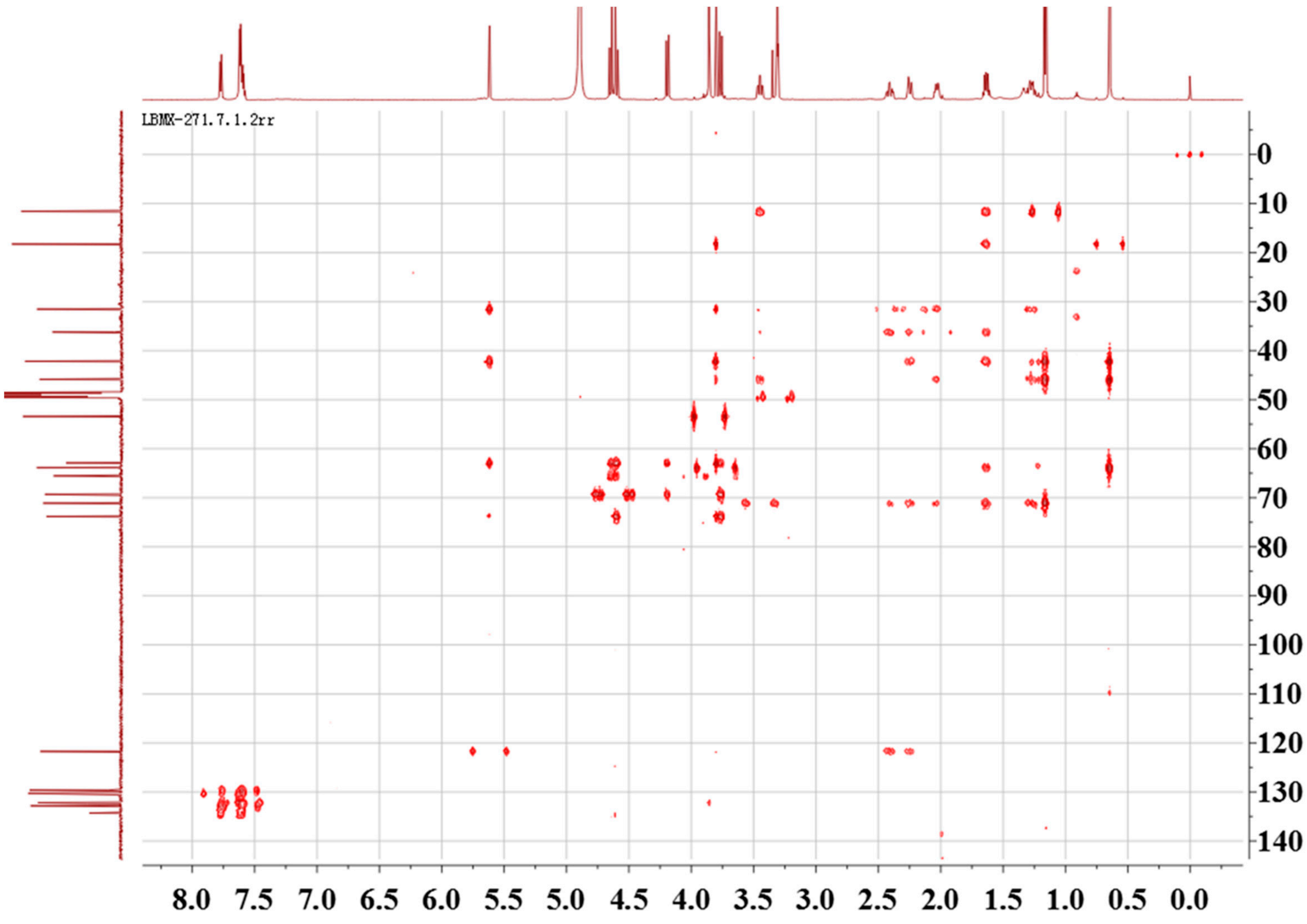

Figure S9 COSY spectrum of **1** in $CD_3OD$

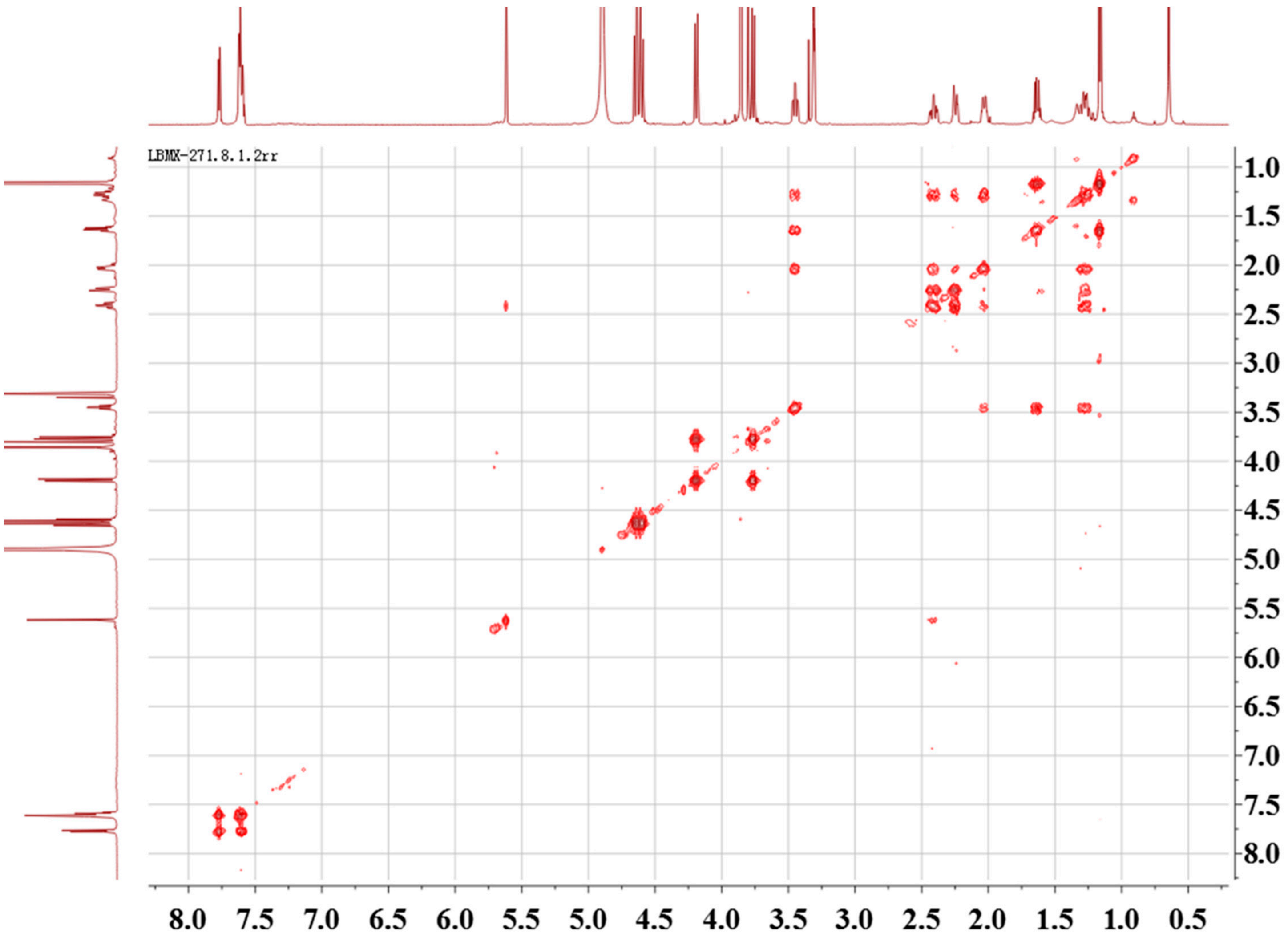

Figure S10 ROESY spectrum of **1** in $CD_3OD$

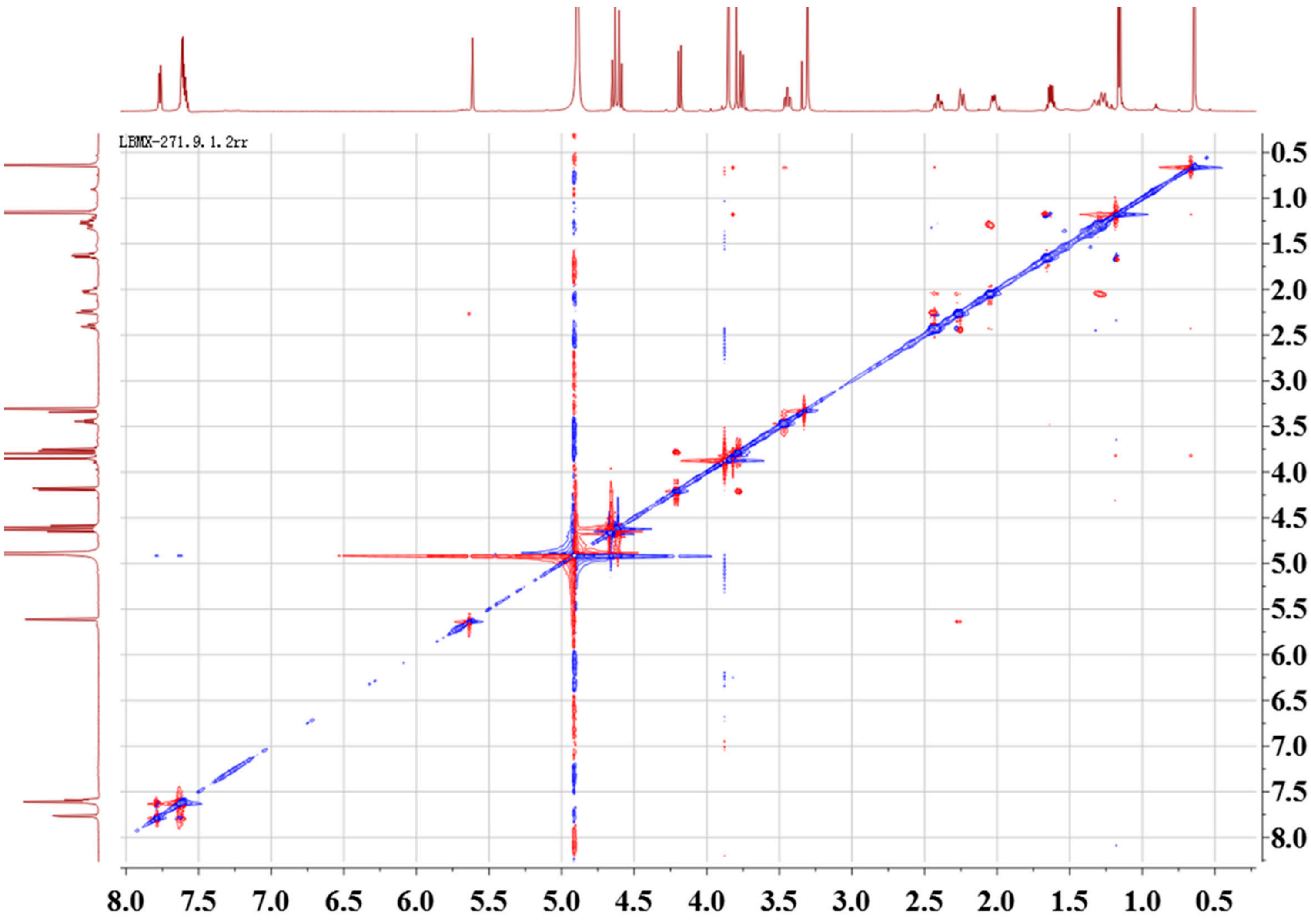

Figure S11 HRMS spectrum of **1**

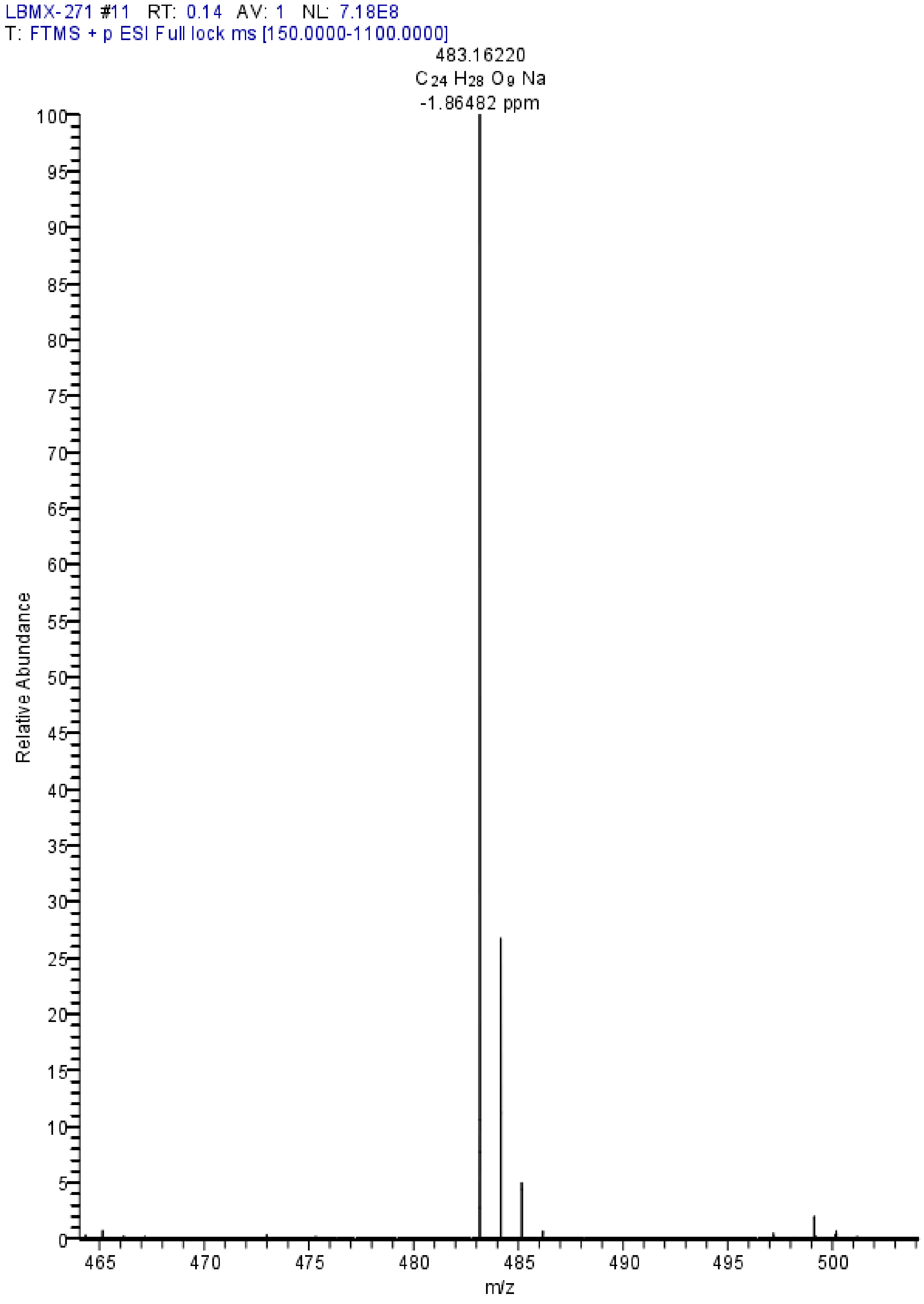

Figure S12 $^{1}H$ NMR spectrum of **2** in $CD_3OD$

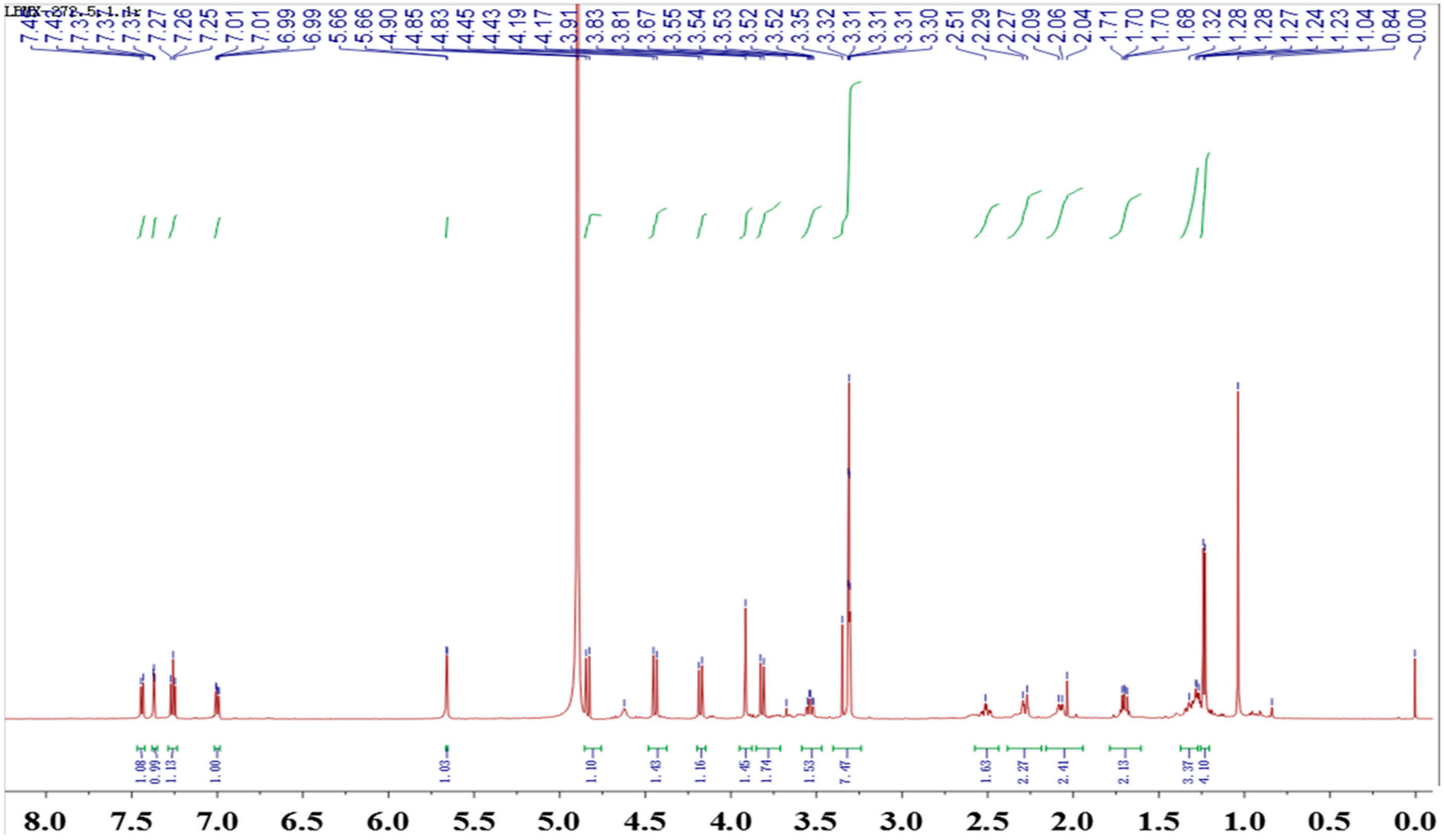

Figure S13 $^{13}C$ NMR spectrum of **2** in $CD_3OD$

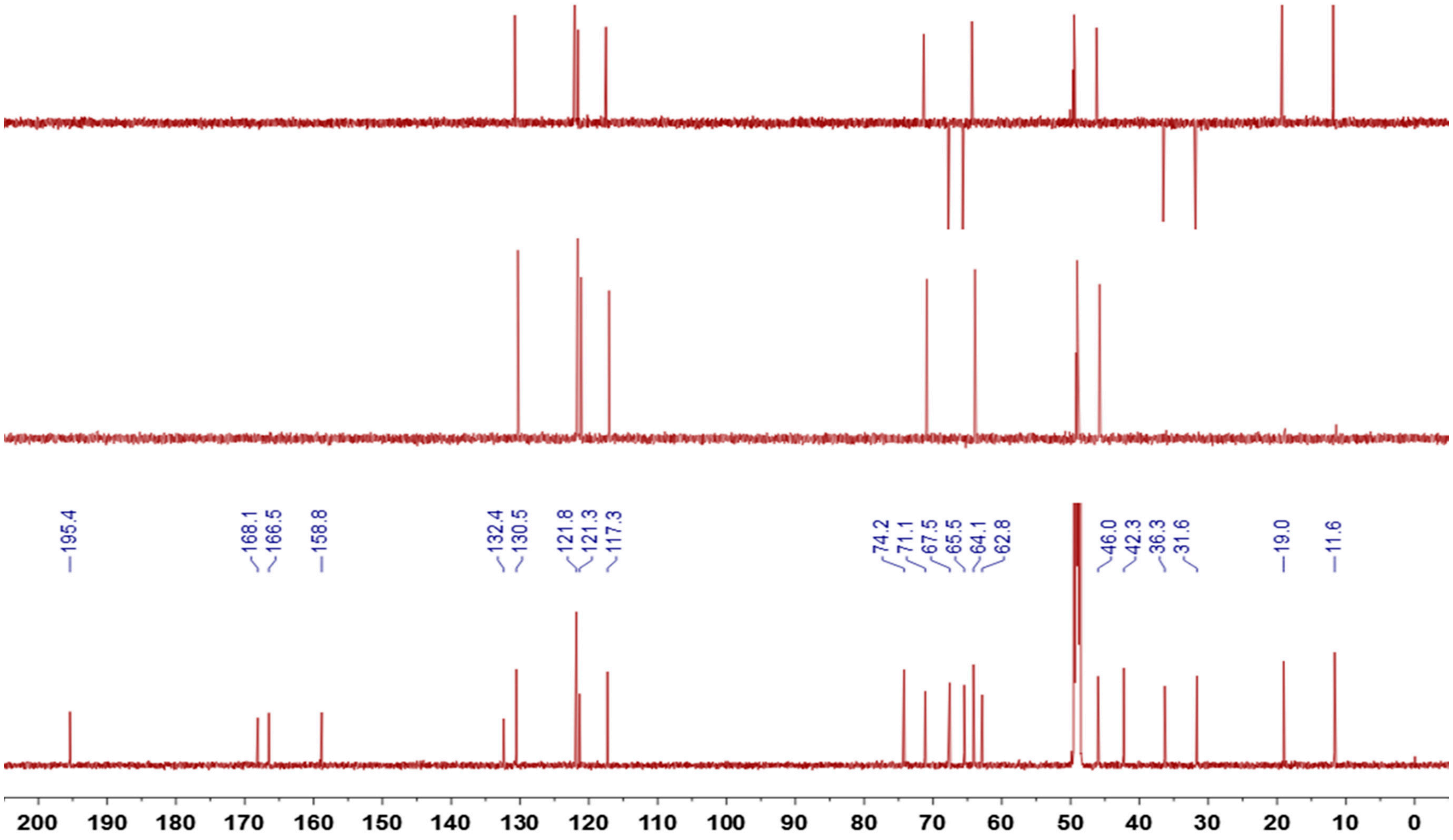

Figure S14 HSQC spectrum of **2** in $CD_3OD$

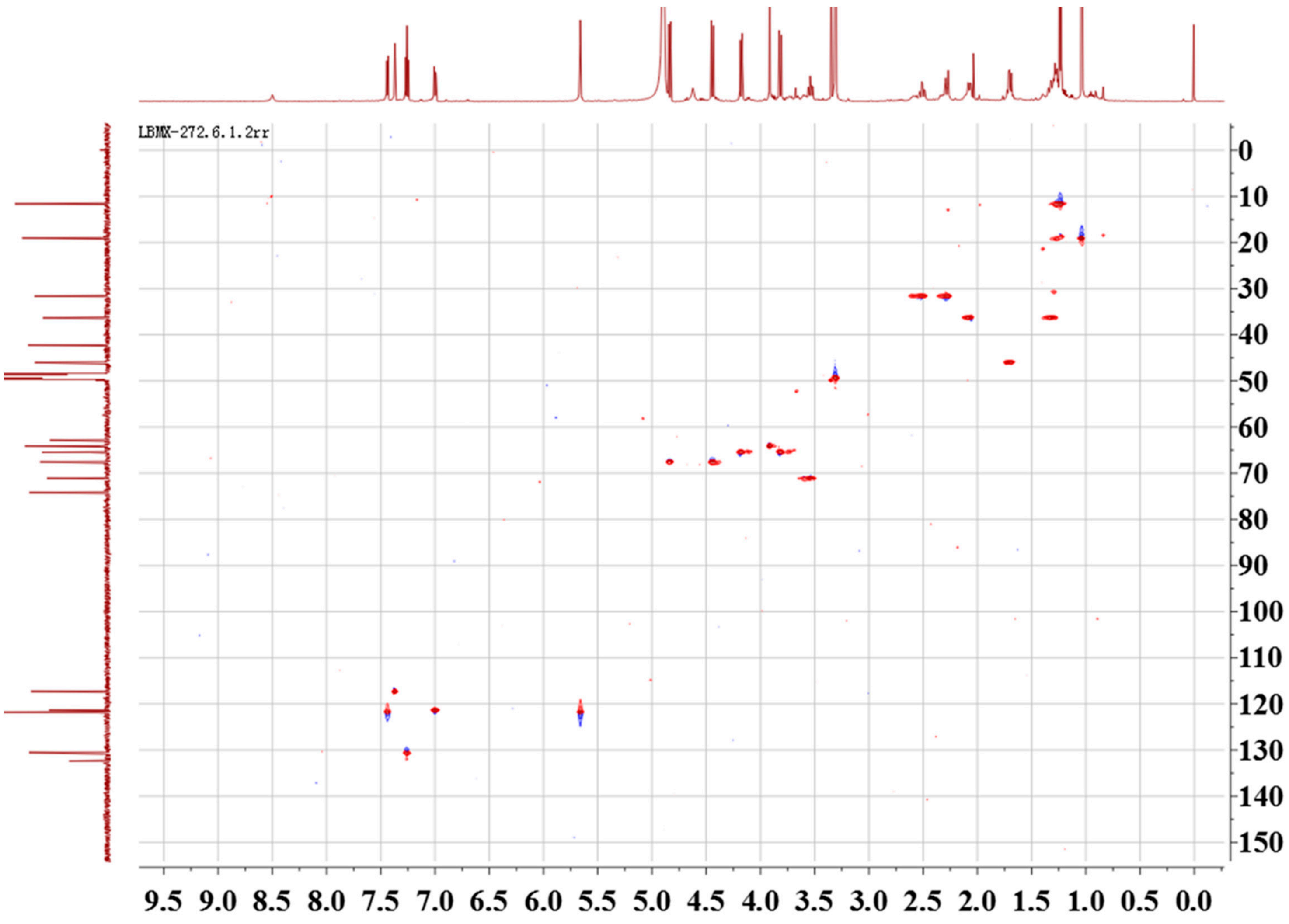

Figure S15 HMBC spectrum of **2** in $CD_3OD$

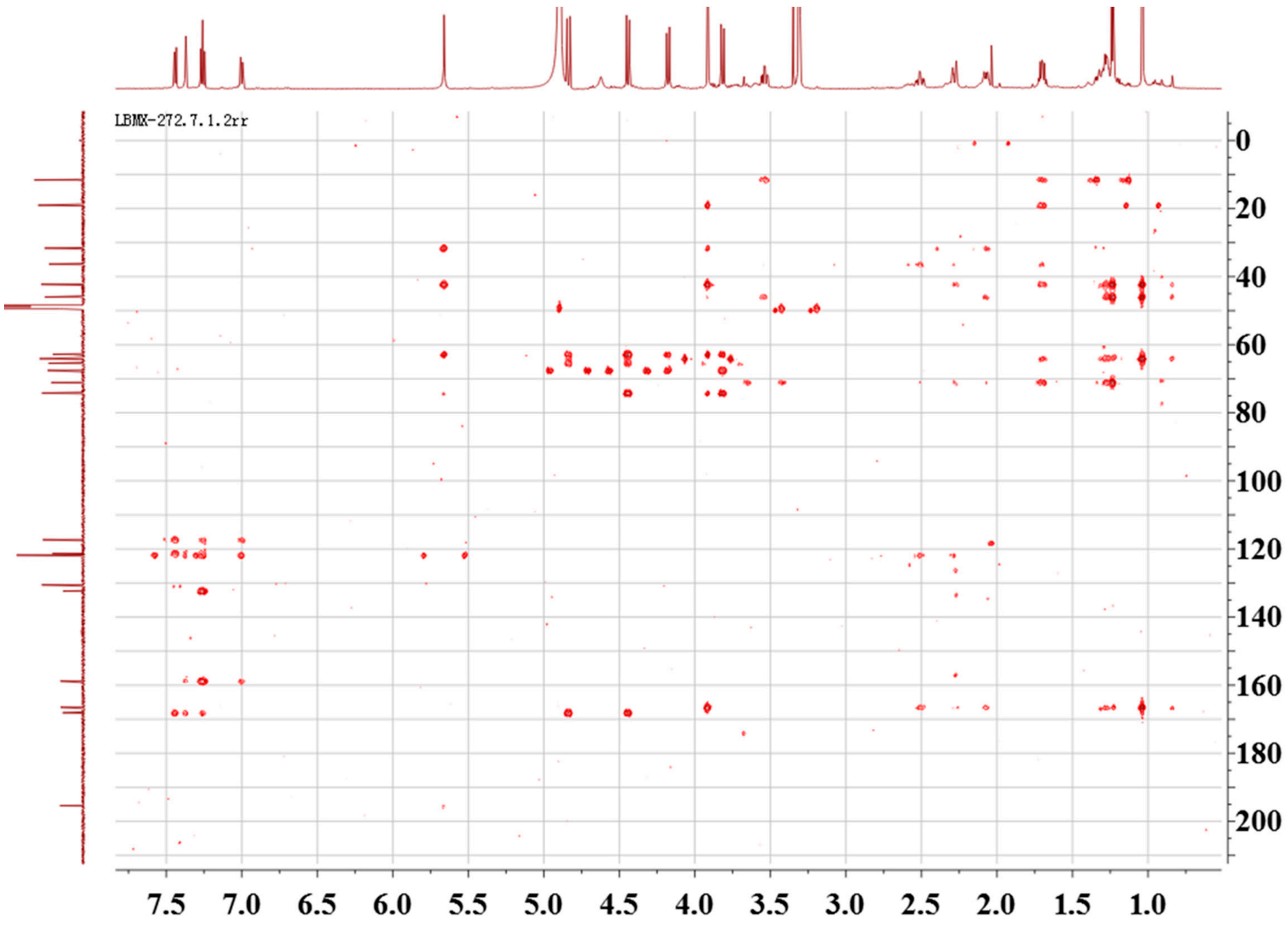

Figure S16 COSY spectrum of **2** in $CD_3OD$

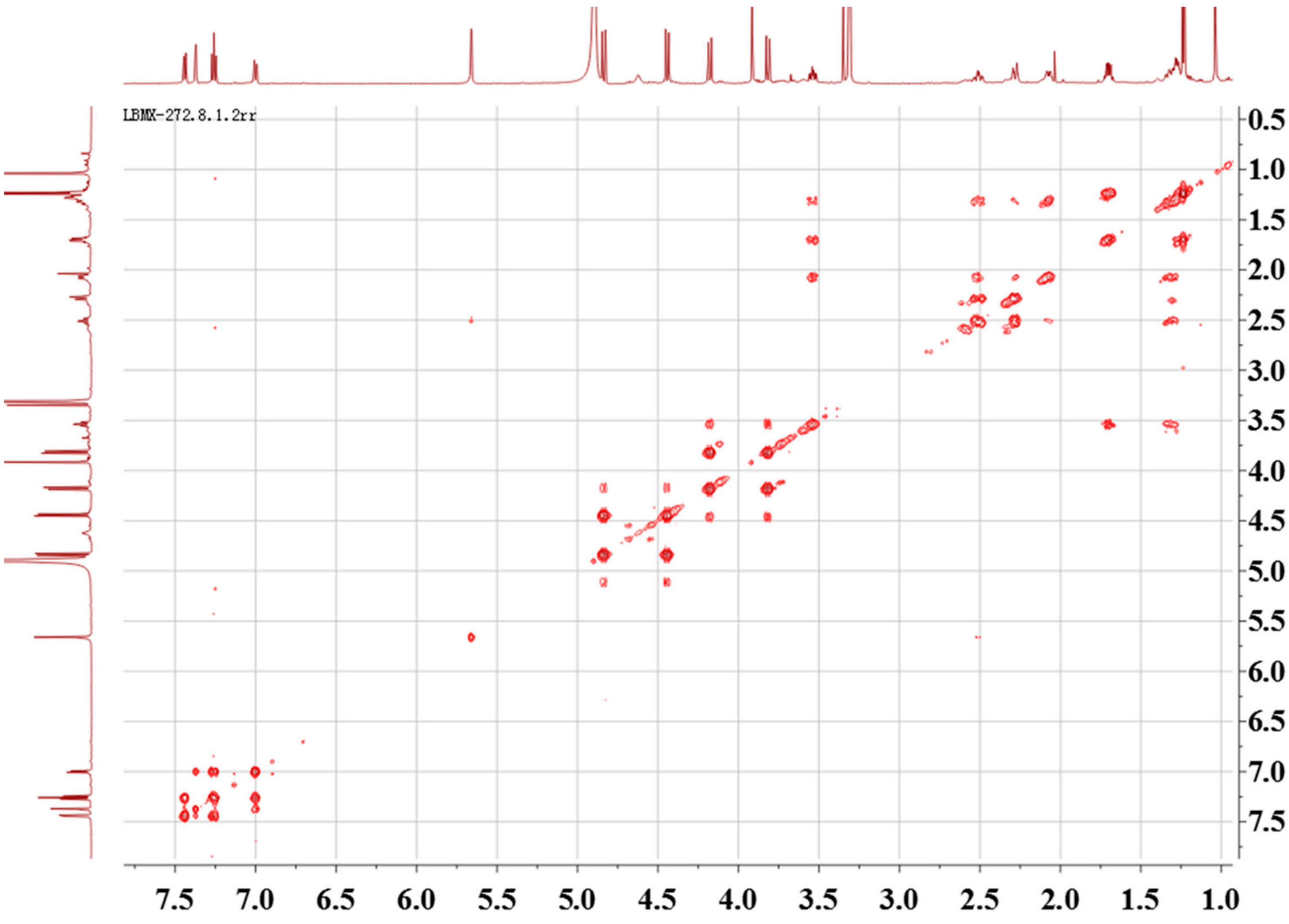

Figure S17 Roesy spectrum of **2** in $CD_3OD$

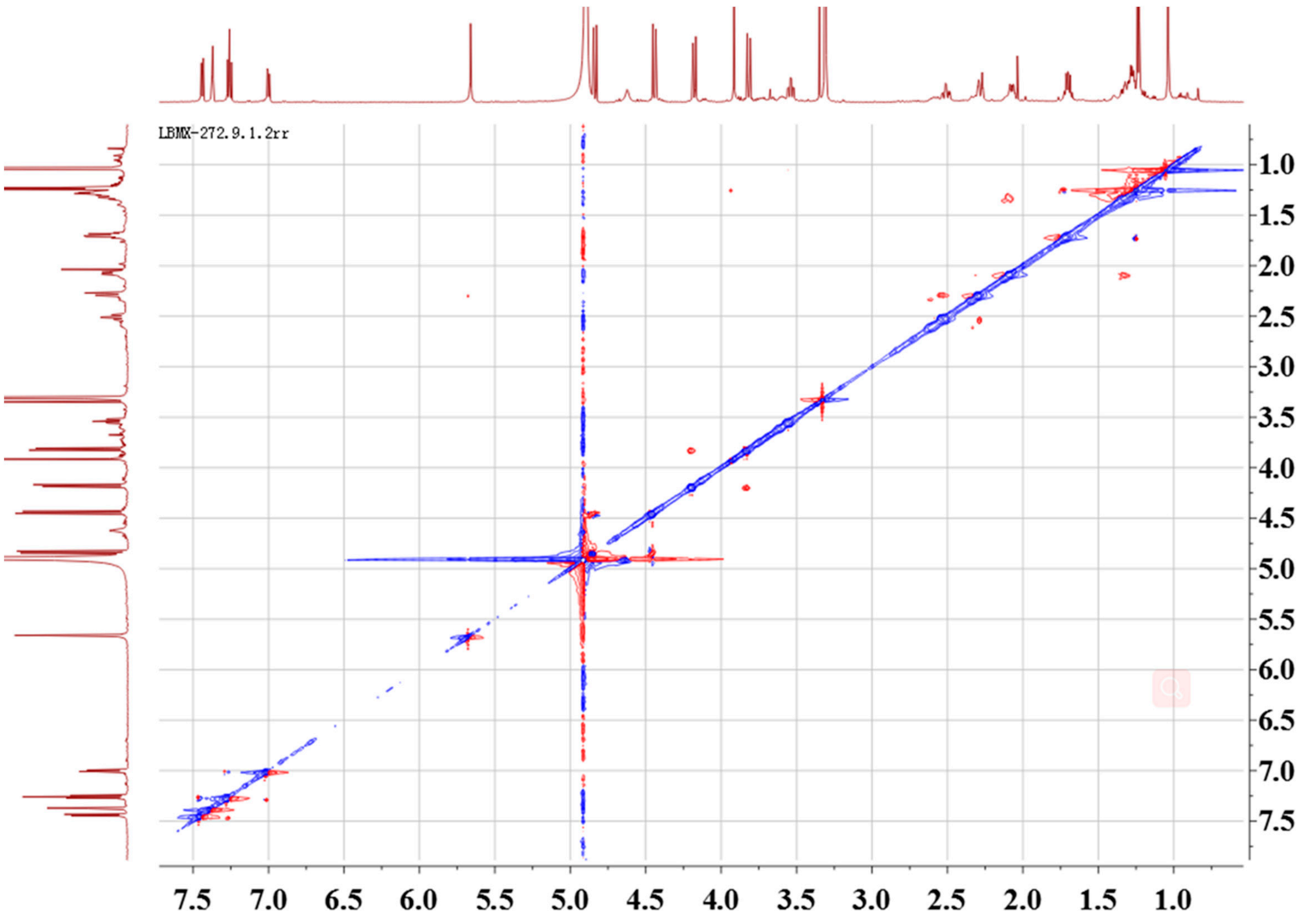

Figure S18 HRMS spectrum of **2**

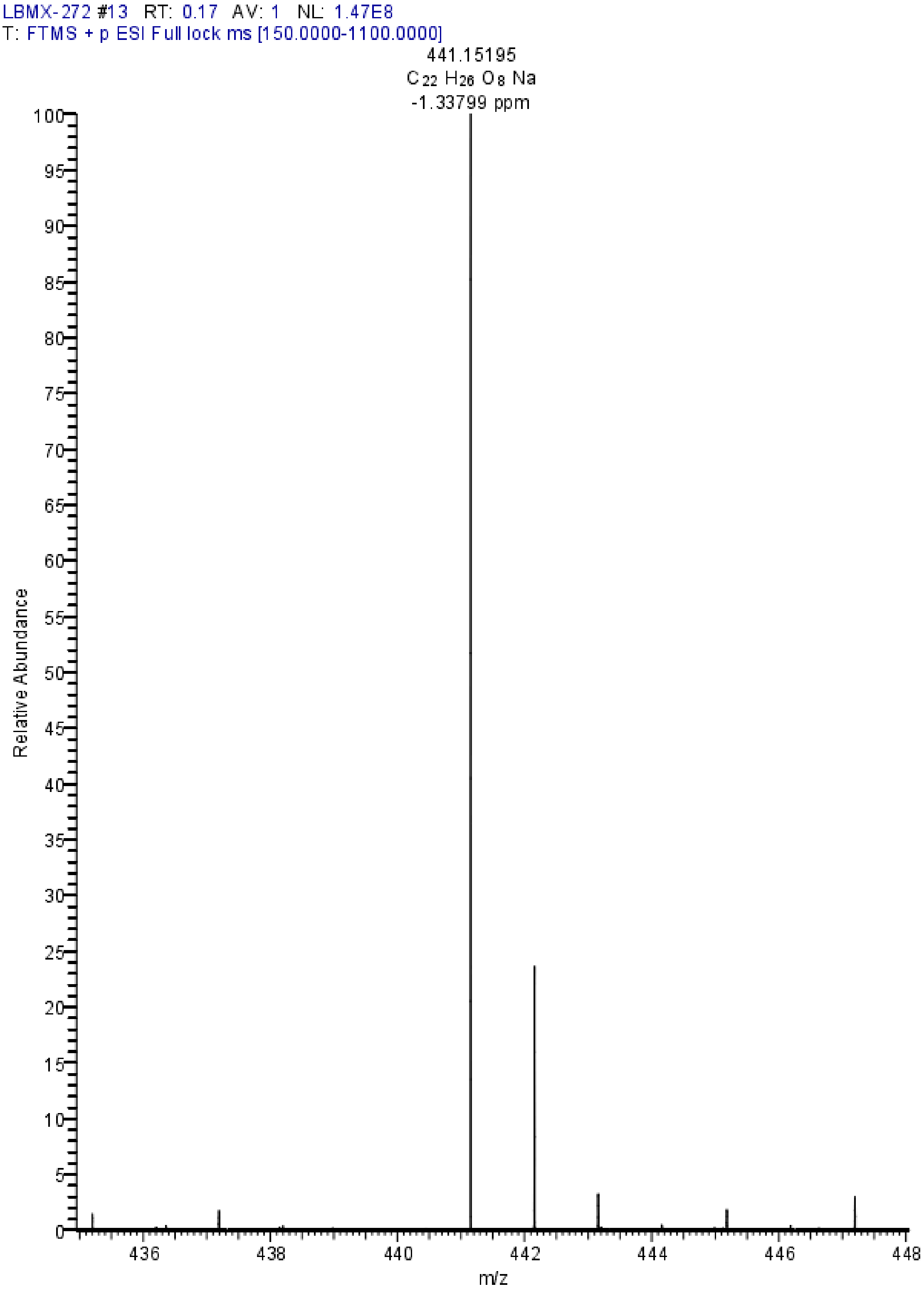

Figure S19 $^{1}$H NMR spectrum of **3** in $CD_3OD$

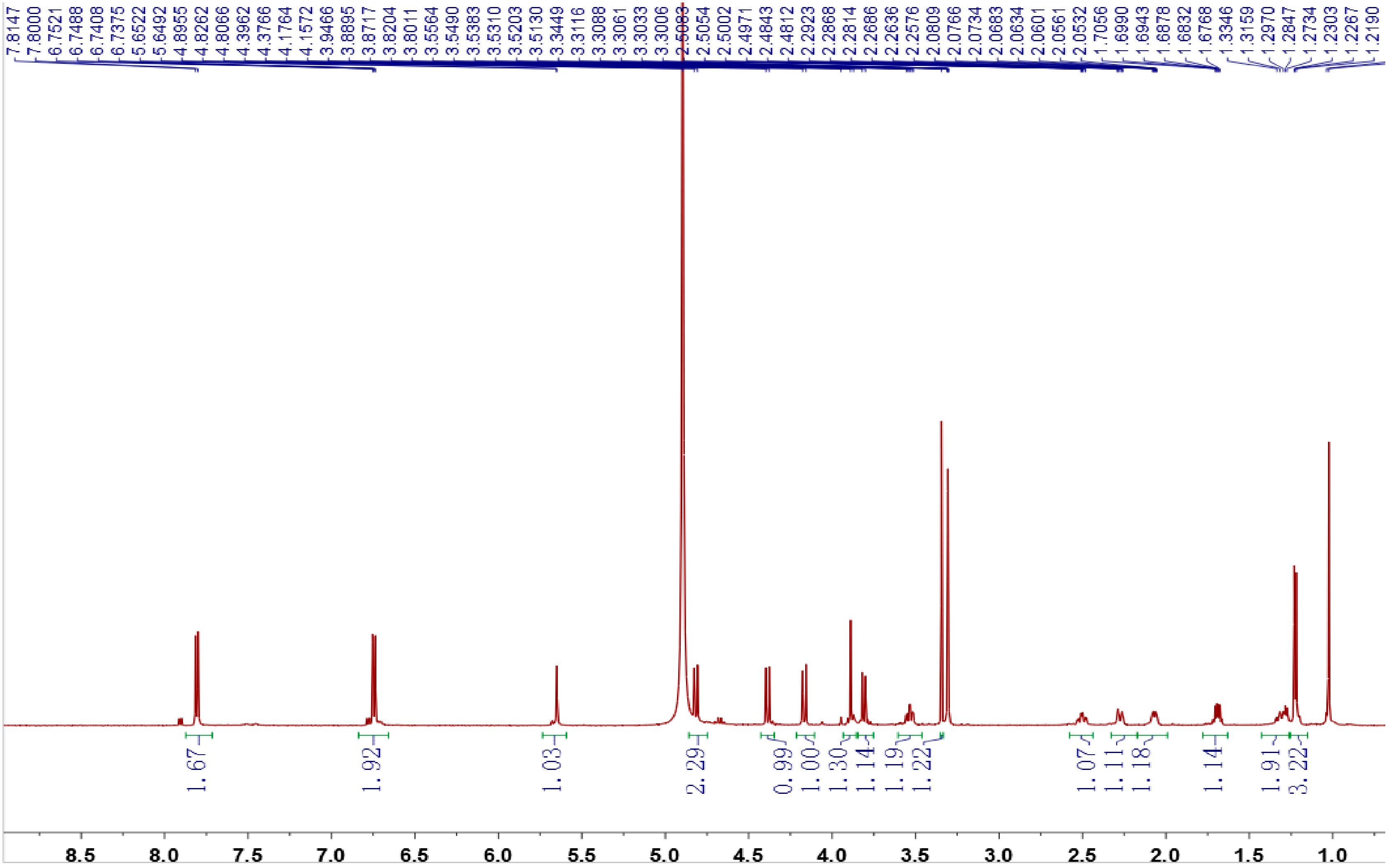

Figure S20 $^{13}C$ NMR spectrum of **3** in $CD_3OD$

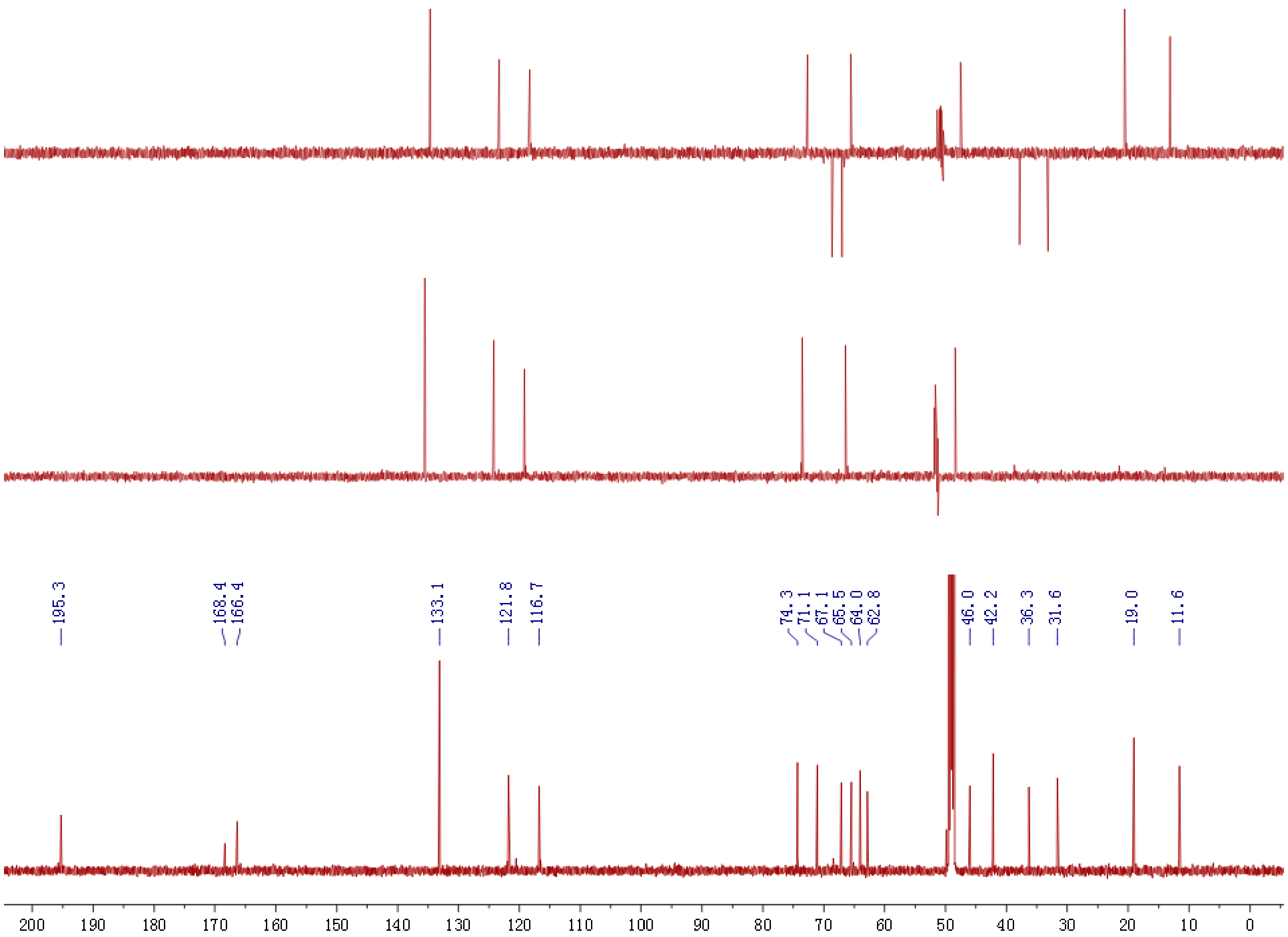

Figure S21 HSQC spectrum of **3** in $CD_3OD$

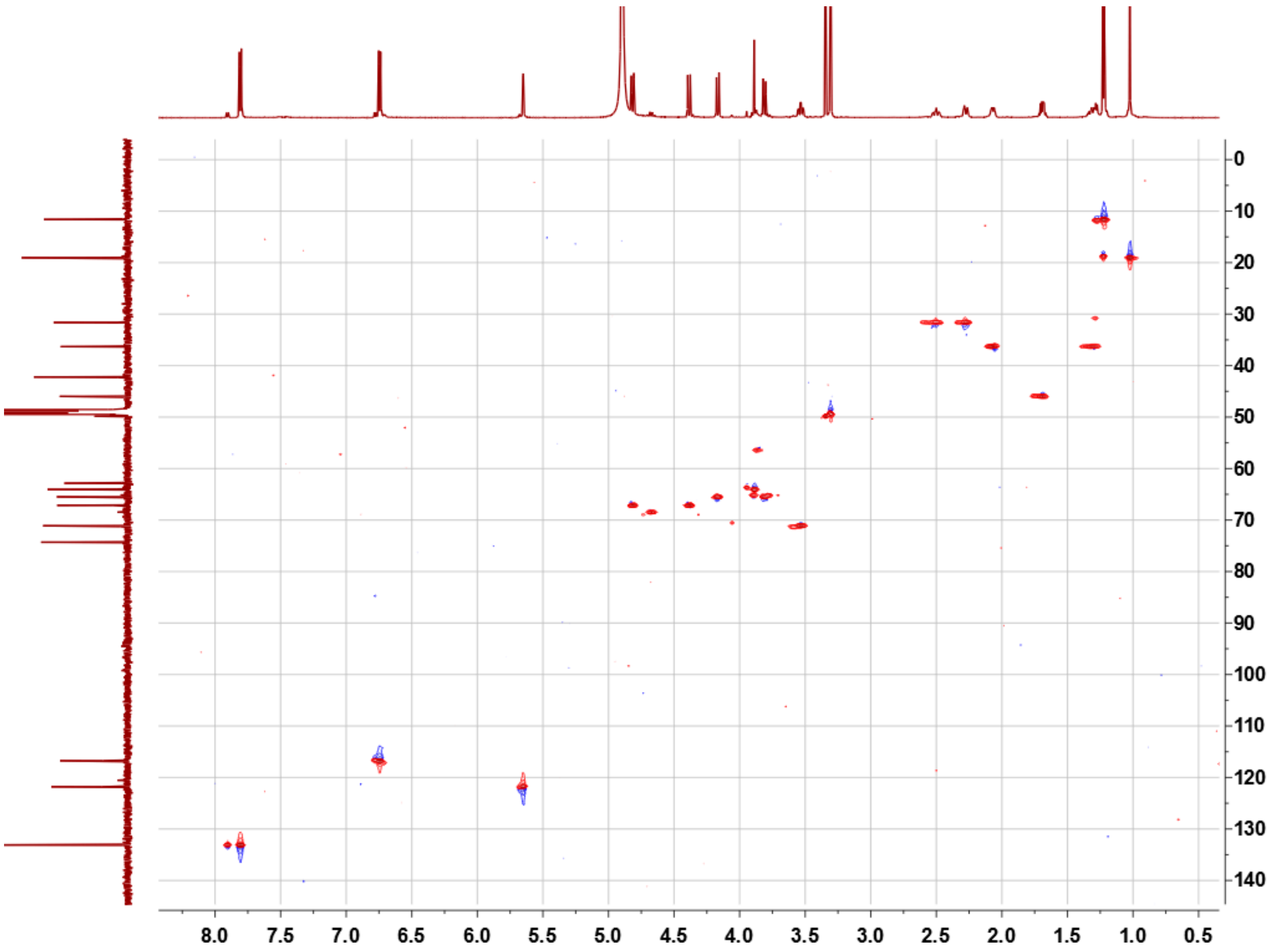

Figure S22 HMBC spectrum of **3** in $CD_3OD$

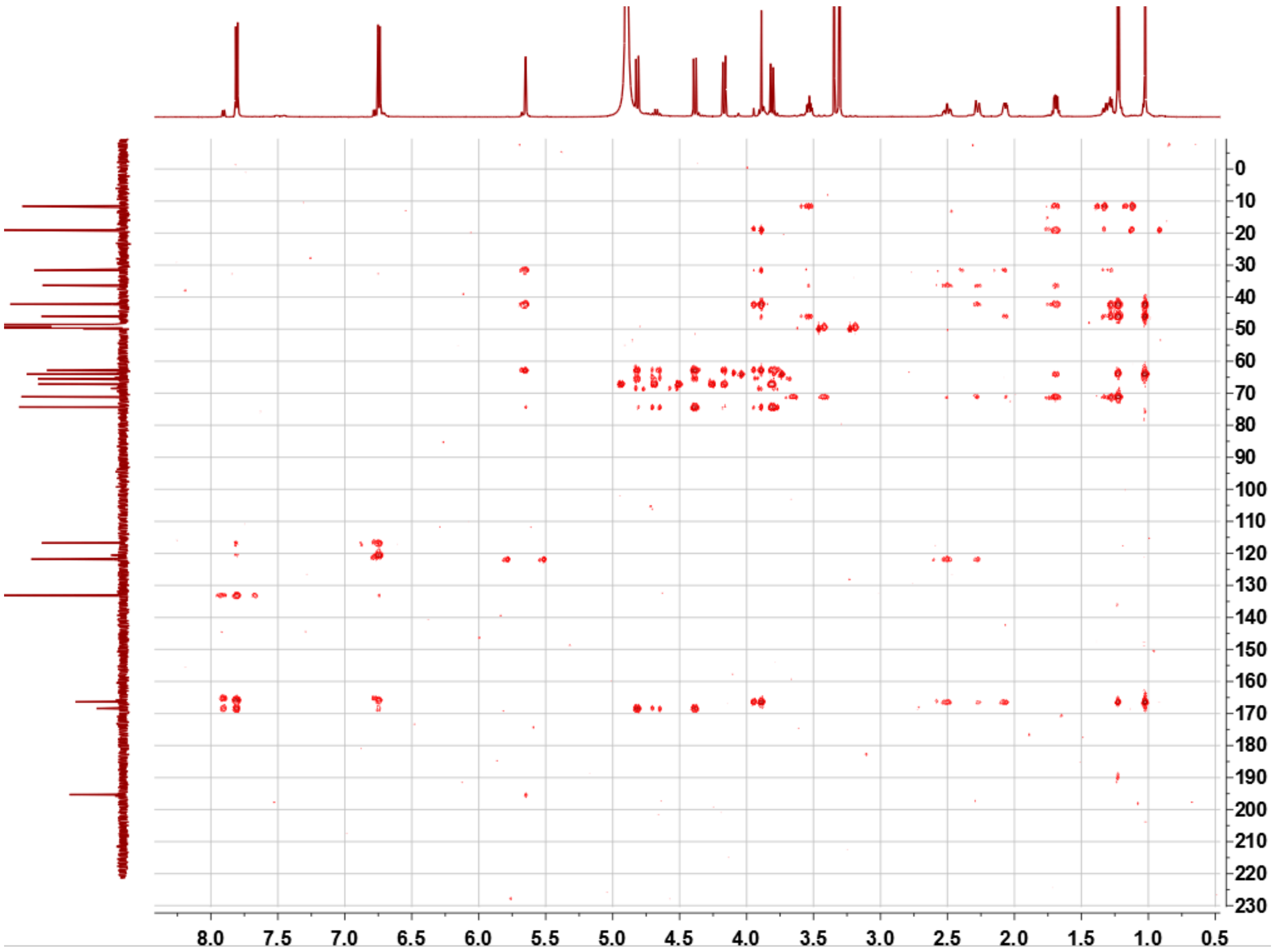

Figure S23 COSY spectrum of **3** in $CD_3OD$

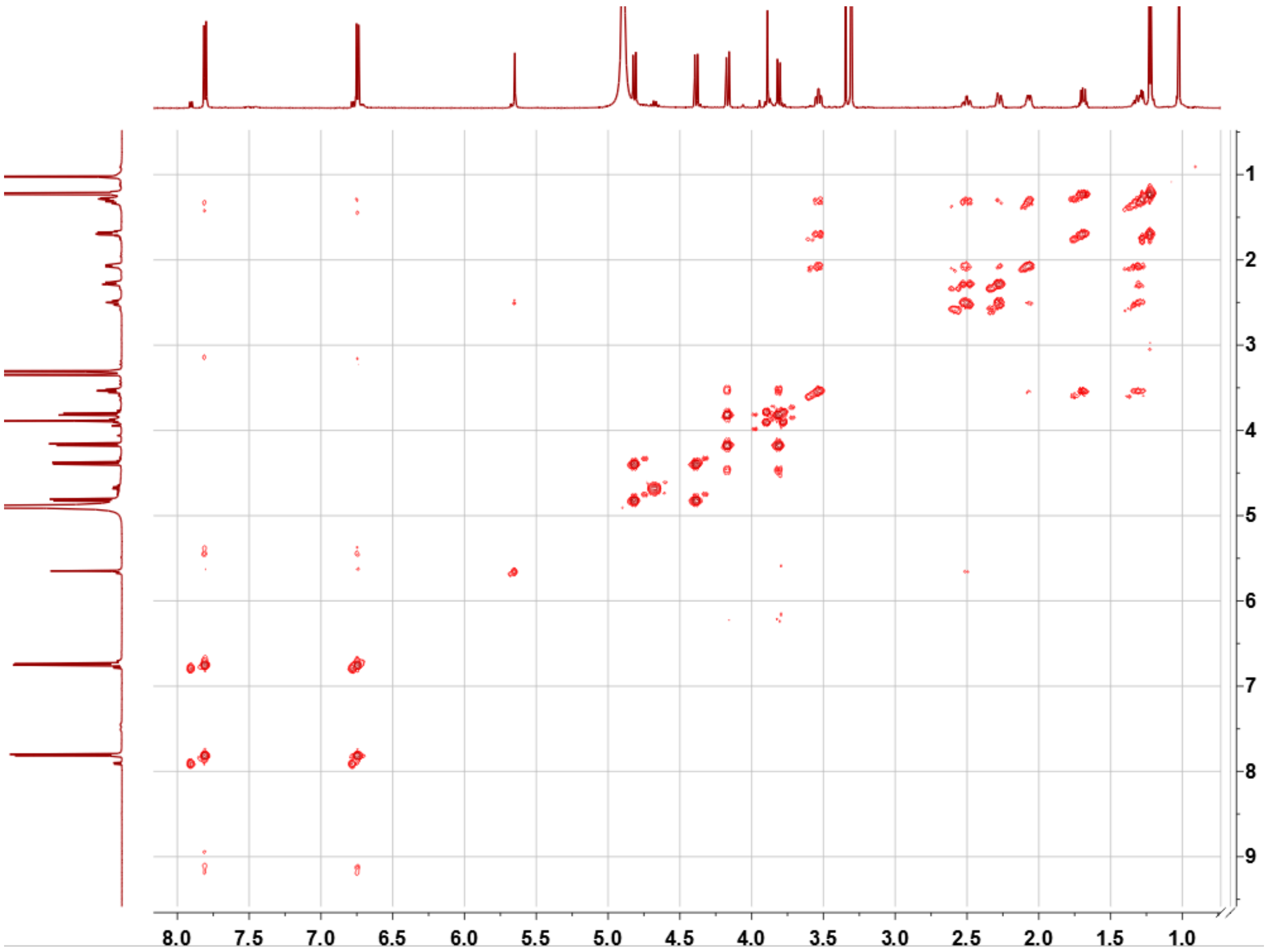

Figure S24 Roesy spectrum of **3** in $CD_3OD$

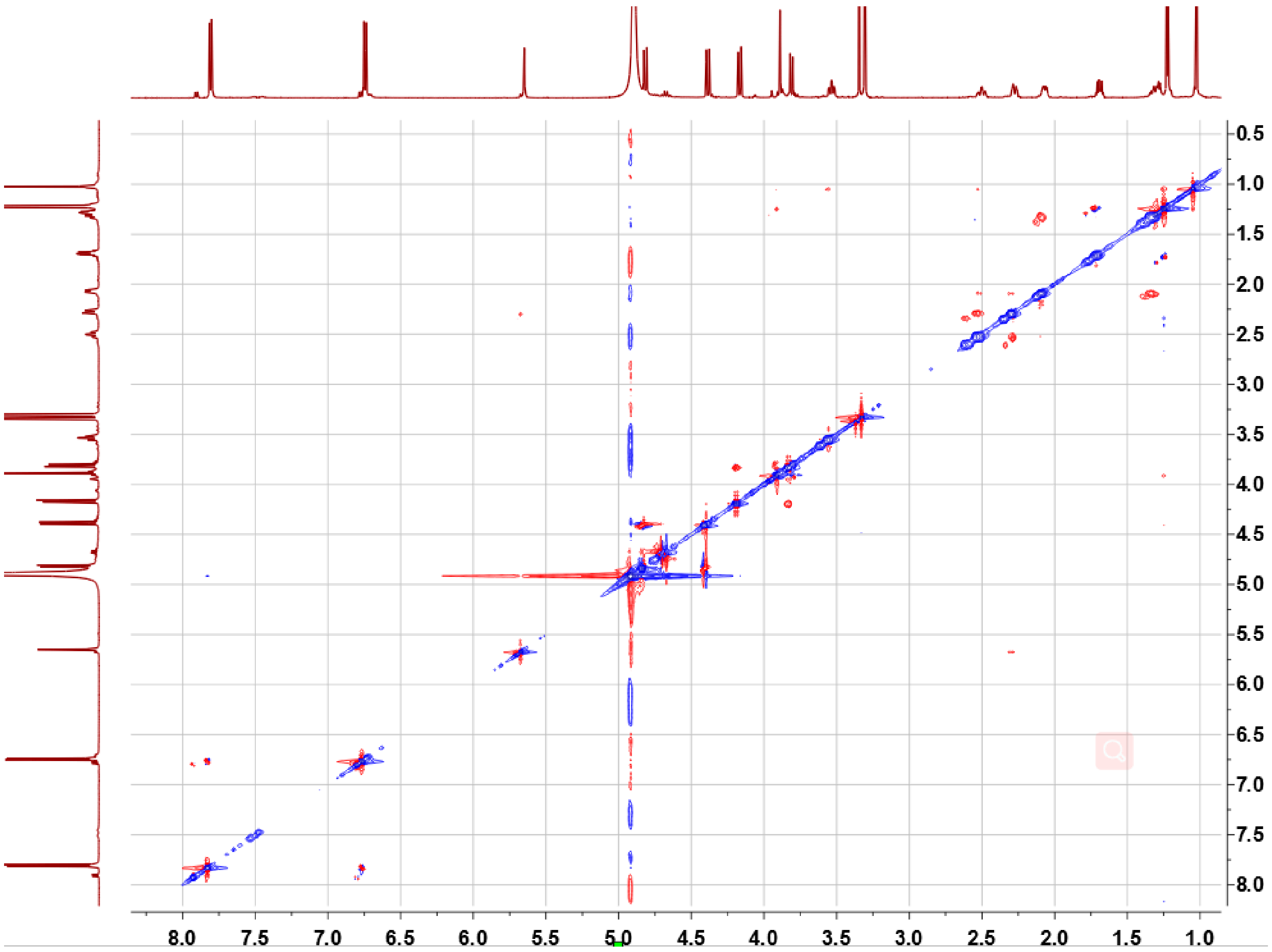

Figure S25 HRMS spectrum of **3**

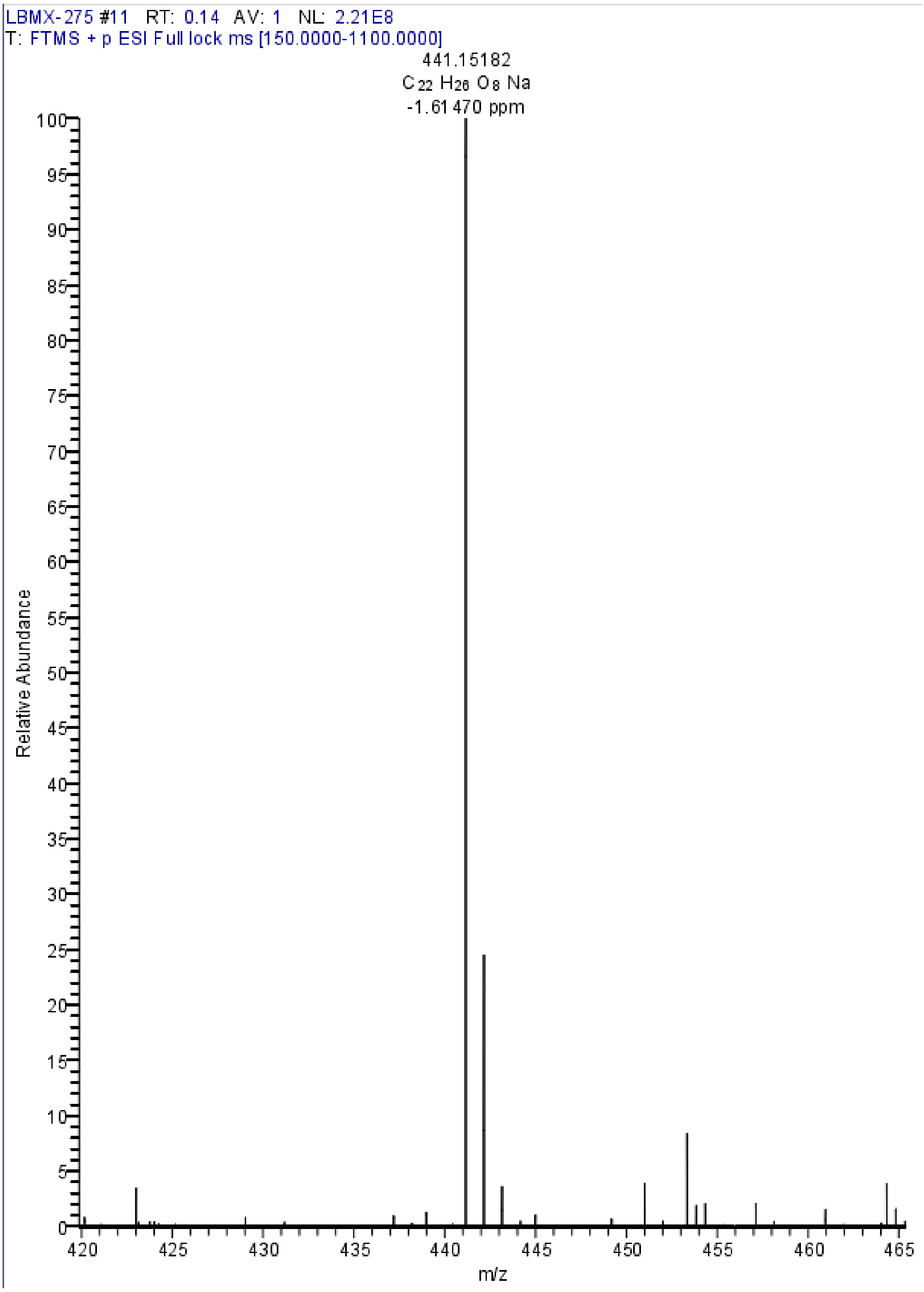

Figure S26 $^{1}$H NMR spectrum of **4** in $CD_3OD$

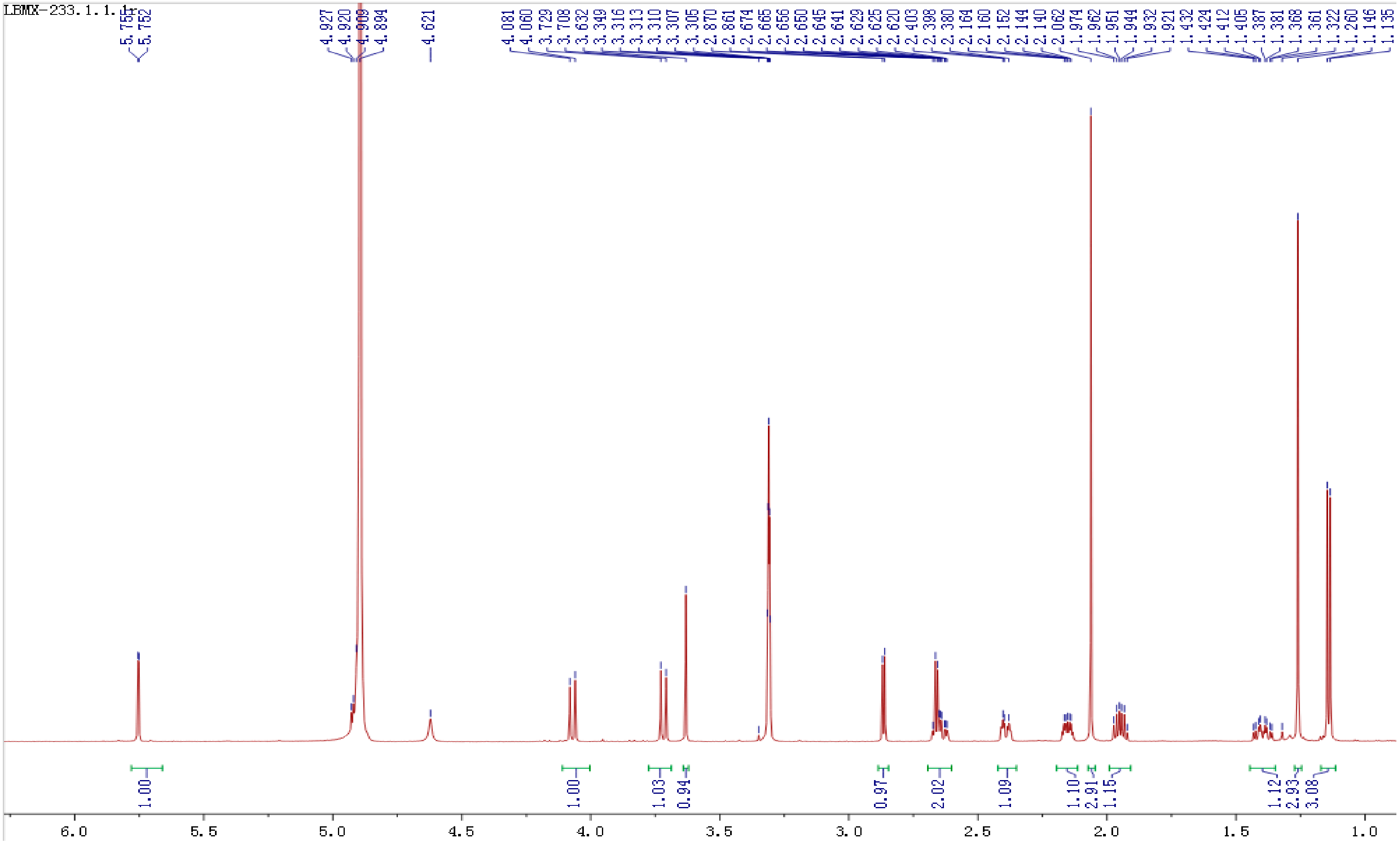

Figure S27 $^{13}C$ NMR spectrum of **4** in $CD_3OD$

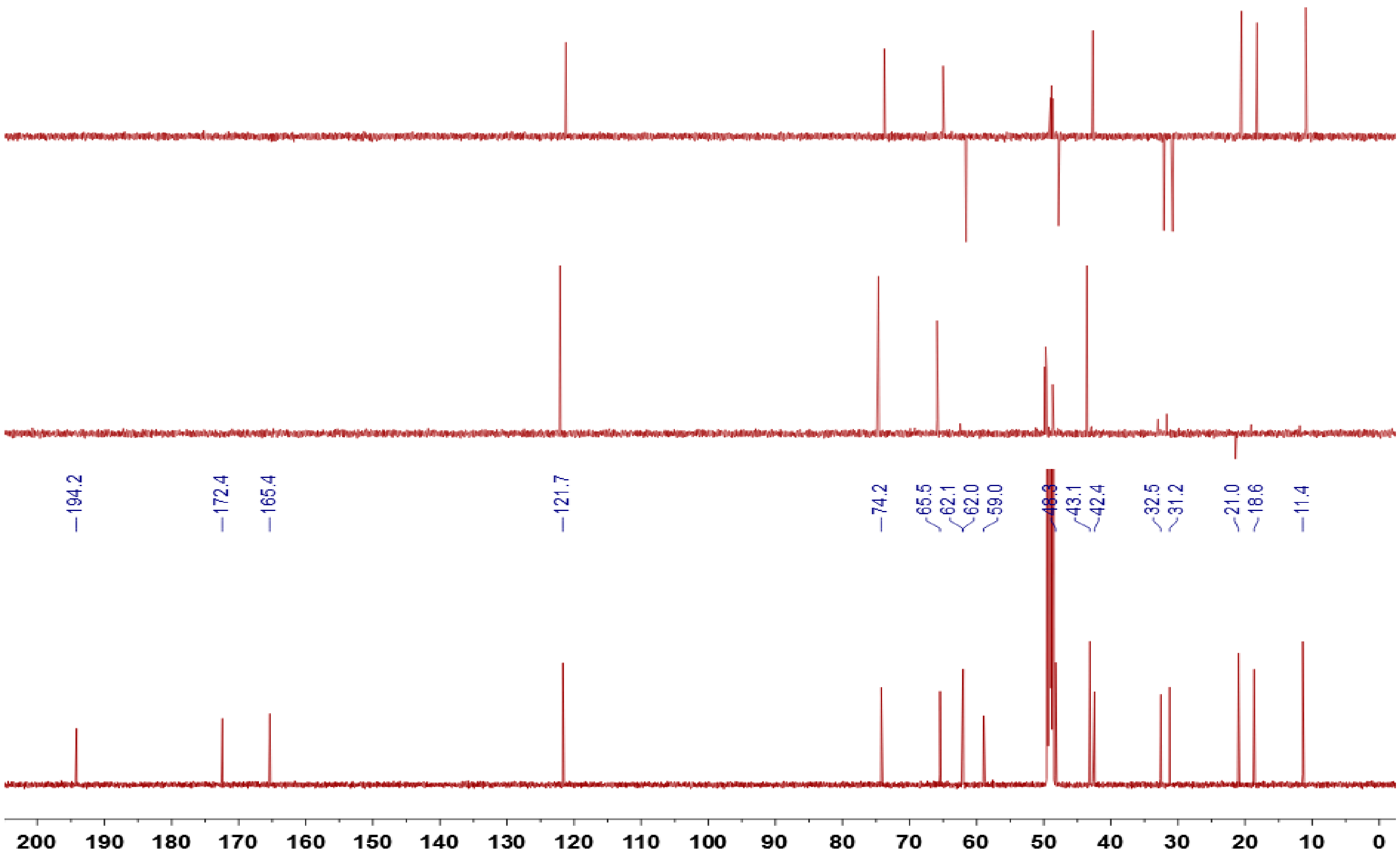

Figure S28 HSQC spectrum of **4** in $CD_3OD$

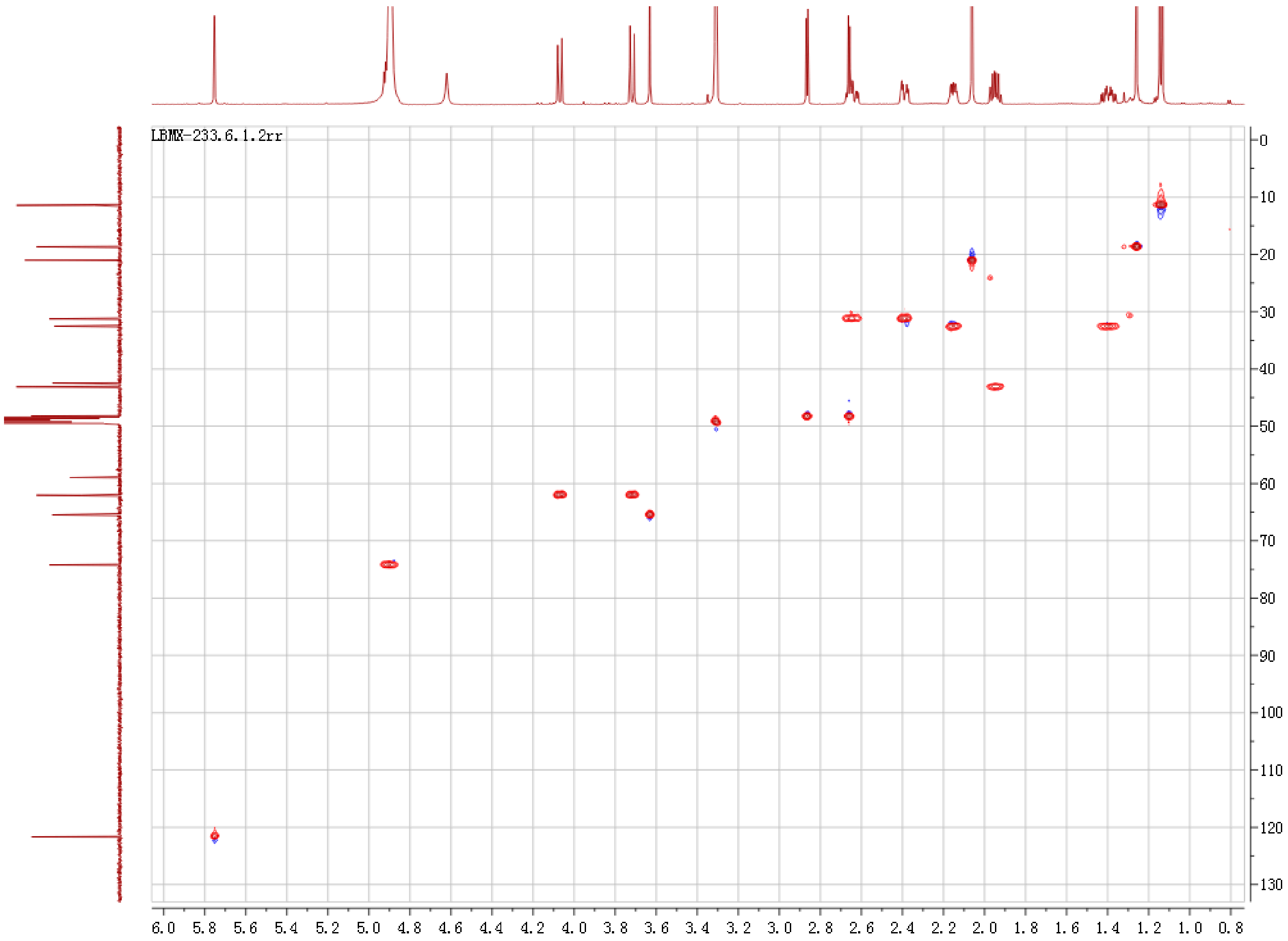

Figure S29 HMBC spectrum of **4** in $CD_3OD$

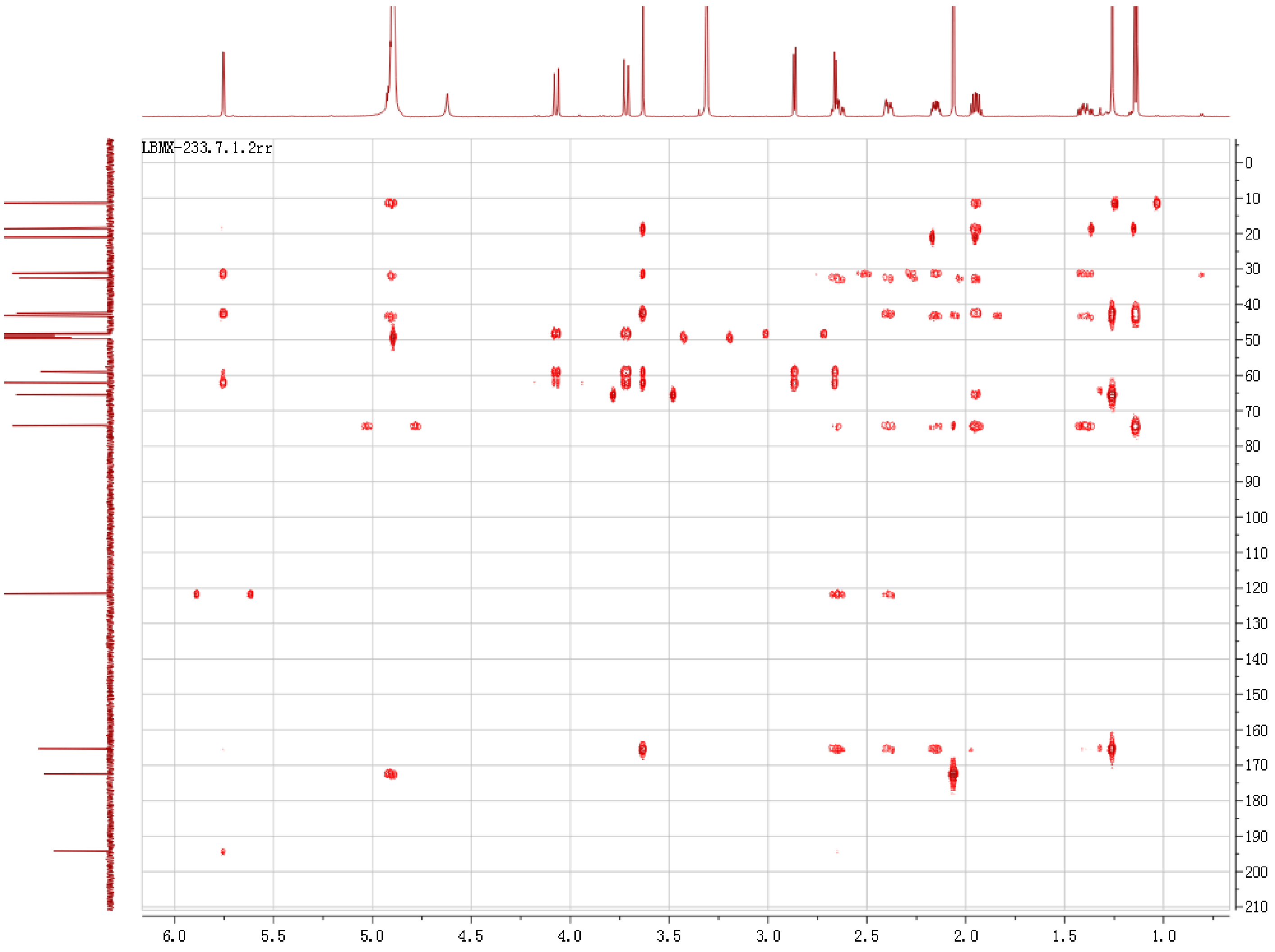

Figure S30 COSY spectrum of **4** in $CD_3OD$

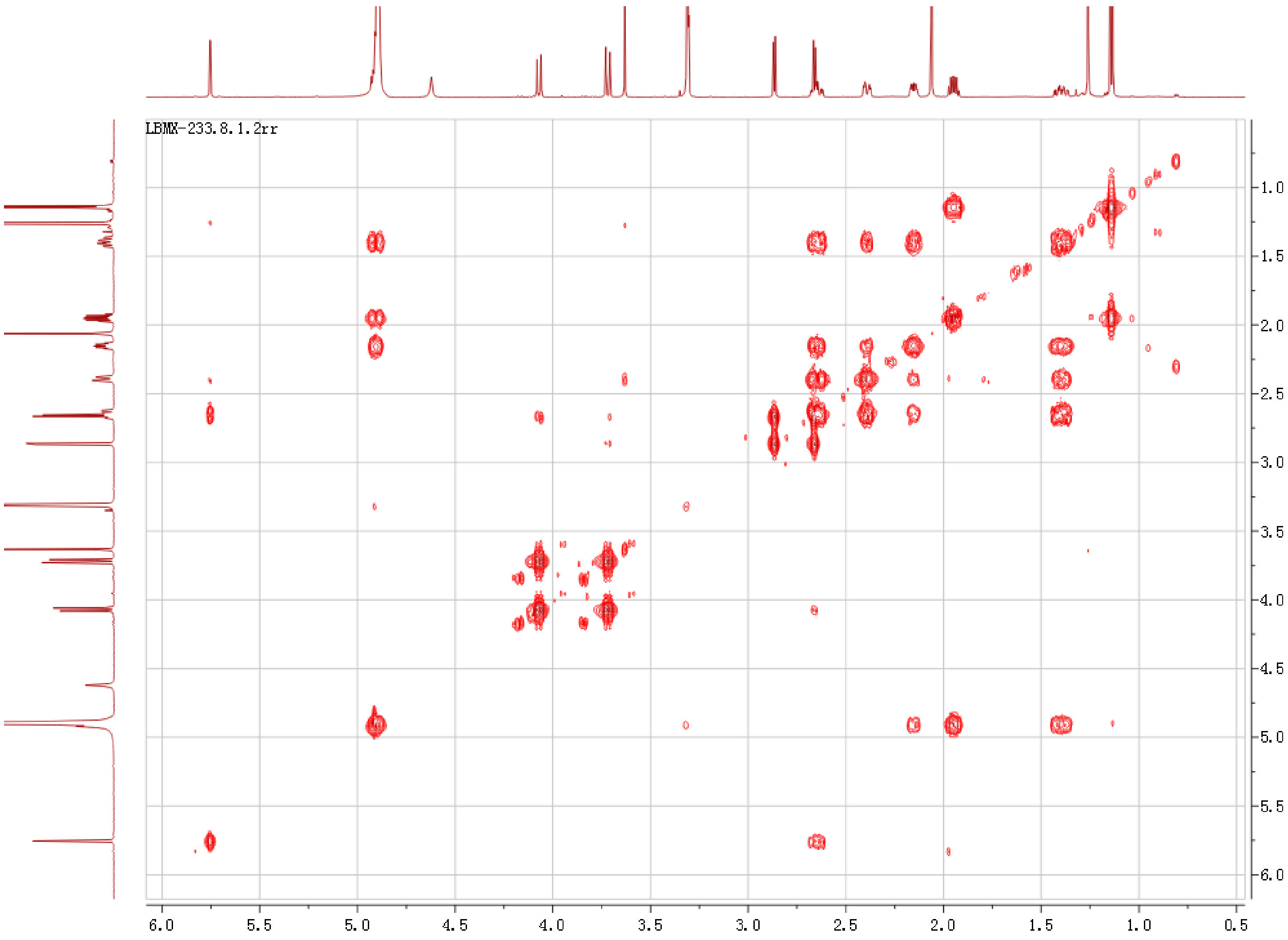

Figure S31 Roesy spectrum of **4** in $CD_3OD$

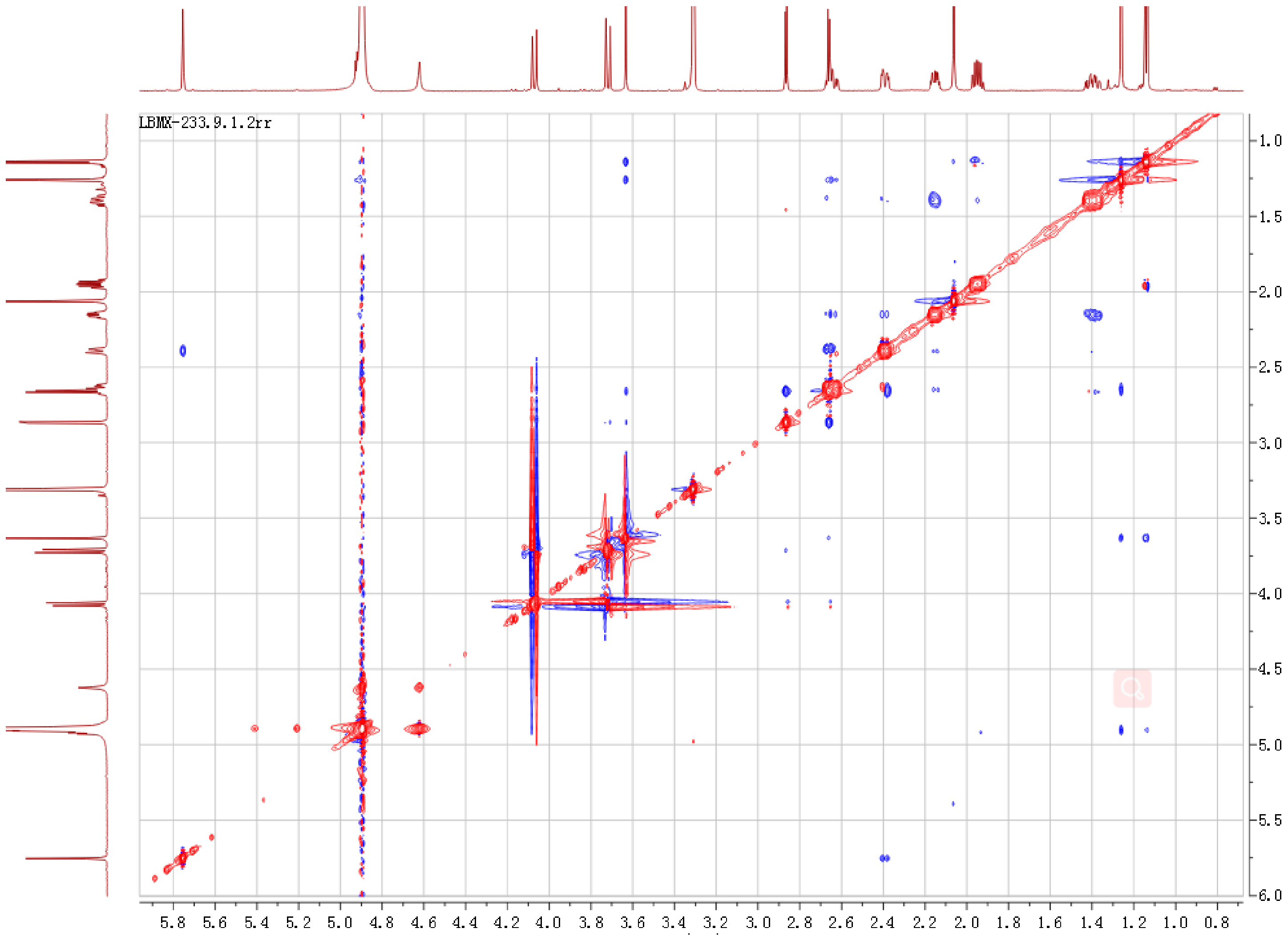

Figure S32 HRMS spectrum of **4**

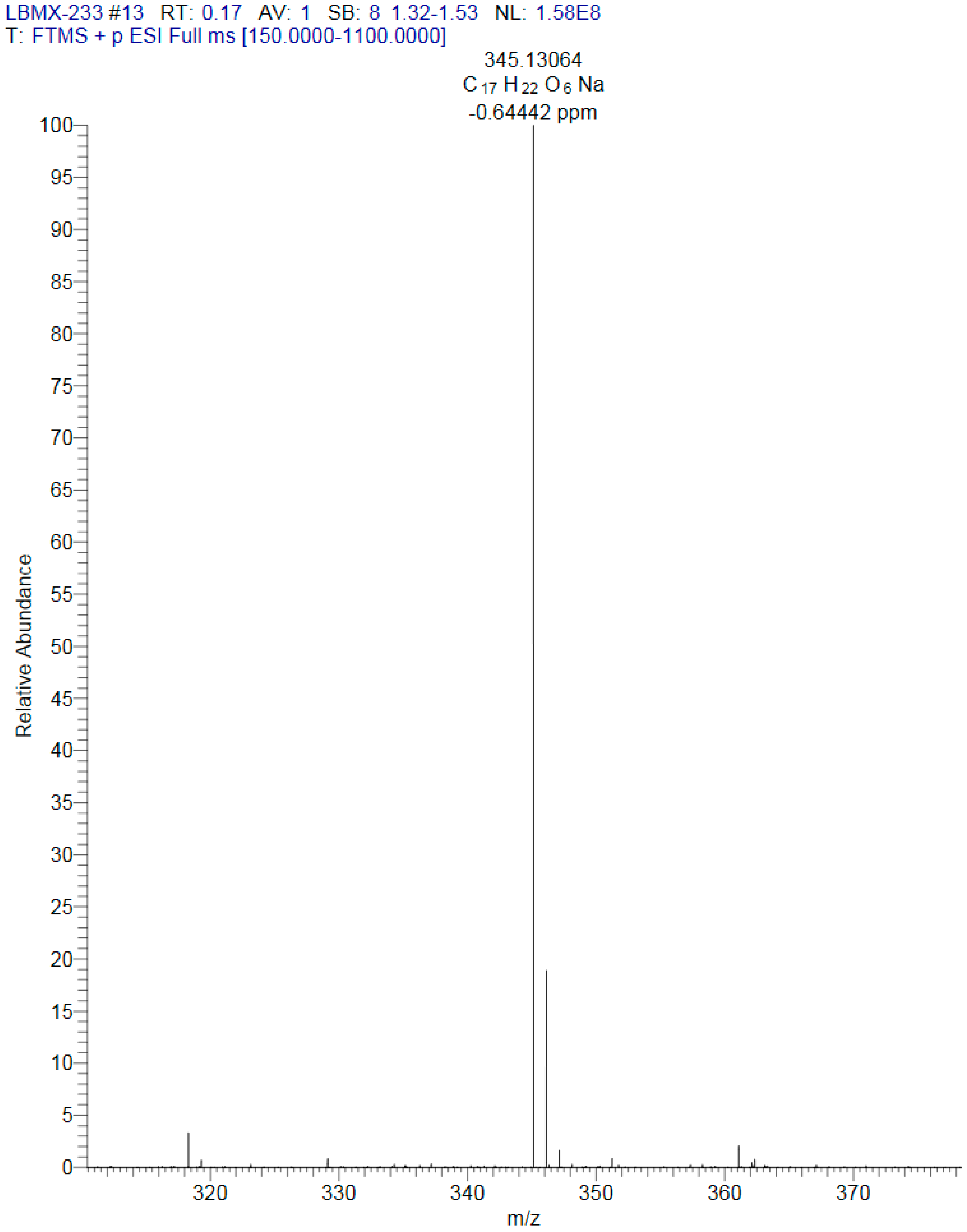

Figure S33 CD spectrum of **1-4** in MeOH

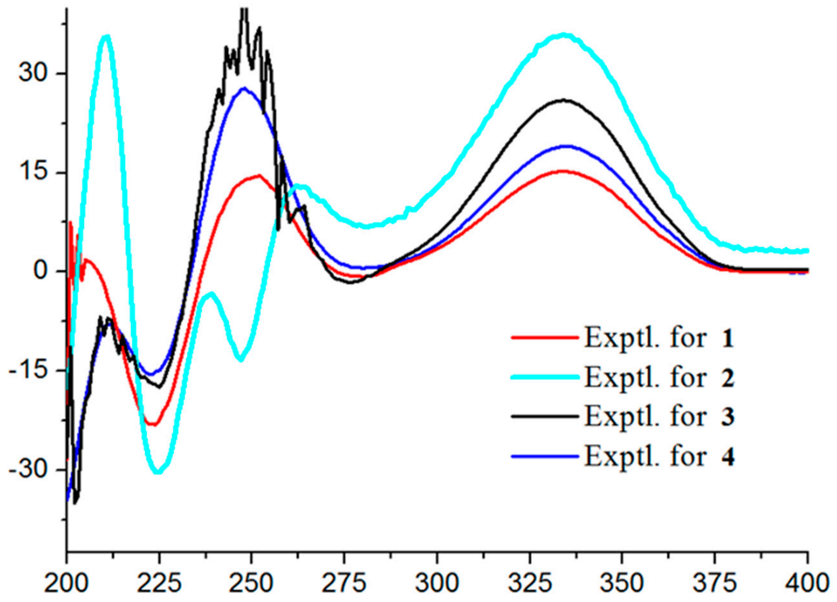

Supplement: Supplementary file 1 [file jof-08-00492-s001.zip › jof-1709647-supplementary.pdf]
